# Adaptive neuro-fuzzy risk index for safe and interpretable lane-change decisions in Level-3 autonomous driving

**DOI:** 10.3389/fnbot.2026.1918469

**Published:** 2026-10-09

**Authors:** Quanda Zu, Shaobo Cai, Jianwei Li

**Affiliations:** 1 Wenzhou Polytechnic University, Wenzhou, Zhejiang, China; 2 Institute of Advanced Manufacturing and Modern Equipment Technology, Jiangsu University, Zhenjiang, China

**Keywords:** Adaptive Neuro-Fuzzy Inference System (ANFIS), connected and autonomous vehicles (CAVs), lane change decision-making, risk assessment, SAE Level 3 automation, Take-Over Request (TOR), Time-to-Collision (TTC)

## Abstract

**Introduction:**

Highway lane-change maneuvers pose a safety challenge for SAE Level 3 autonomous driving because conventional threshold-based decisions and isolated surrogate safety measures may inadequately capture the coupling between longitudinal braking demand and evolving inter-vehicle gaps. This study develops an interpretable, risk-aware framework for continuous lane-change assessment during maneuver initiation and execution.

**Methods:**

An adaptive neuro-fuzzy inference system (ANFIS) combines normalized longitudinal braking demand and minimum inter-vehicle gap to generate a continuous risk index ranging from zero to one. The index is integrated into a two-phase decision mechanism incorporating multi-vehicle threat assessment, region-dependent maneuver cancellation, quintic Bézier trajectory replanning, and driver takeover supervision. Performance was evaluated through high-fidelity SCANeR/ReScan and MATLAB/Simulink co-simulations against static Mamdani fuzzy, time-to-collision (TTC), deceleration-demand, and time-headway baselines. Robustness was assessed using 1,000 Monte Carlo simulations with variations in vehicle speeds, sensor noise, road friction, and cut-in timing.

**Results:**

Compared with the TTC baseline, the proposed framework increased warning lead time from 0.86 to 1.84 s and minimum two-dimensional clearance from 2.08 to 4.82 m, while reducing maximum deceleration demand from 4.92 to 2.34 *m*/*s*^2^. Mean computational execution time was 1.42 ms, below the 10-ms simulation control-cycle budget. Across the 1,000 randomized simulations, no collisions were observed with the proposed framework; near-miss and false-alarm rates were 0.30% and 1.20%, respectively.

**Discussion:**

The simulation findings support the potential of continuous neuro-fuzzy risk assessment to provide earlier hazard detection and larger safety margins while retaining rule-based interpretability and low computational cost. Hardware-in-the-loop and real-vehicle testing remain necessary to establish performance beyond the evaluated simulation conditions.

## 1 Introduction

Autonomous vehicles (AVs) and connected and autonomous vehicles (CAVs) have emerged as pivotal technological catalysts within intelligent transportation systems (ITS), offering revolutionary potential for drastically minimizing traffic collisions, mitigating congestion bottlenecks, and optimizing transport energy efficiency (Yang et al., 2023; Chai et al., 2017; Darwito and Indayu, 2023). Driven by sustained breakthroughs in multisensory perception systems, edge computing, and real-time motion control algorithms, modern automated driving platforms are increasingly empowered to navigate highly dynamic, unstructured Operational Design Domains (ODDs) with decreasing levels of human supervisory intervention (Chu et al., 2022; Wu et al., 2025). Among the diverse spectrum of tactical driving behaviors, lane change (LC) maneuvers represent an indispensable yet intricate capability on multi-lane freeways and urban expressways, fundamentally dictating road capacity, traffic flow stability, and passenger comfort (Najem et al., 2023; Lu et al., 2023).

Unlike standard car-following tasks that are predominantly constrained to single-dimensional longitudinal spacing adjustments, lane change maneuvers inherently constitute a multi-vehicle spatio-temporal coordination challenge characterized by tight cross-directional kinematic and dynamic couplings (Khalaji and Jalalnezhad, 2021a,b; Jalalnezhad et al., 2024). The ego vehicle must concurrently monitor, predict, and negotiate state trajectories with multiple surrounding agents across both the host and target lanes—specifically leading and trailing vehicles—under severe non-holonomic constraints, varying road surface frictions, and perceptual uncertainties (Jalalnezhad, 2025; Narayana and Vamsi, 2026). In high-speed highway scenarios, improper gap acceptance, latency in threat recognition, or abrupt steering inputs during lane changes represent the principal statistical etiology of severe side-swipe and rear-end collisions (Wang et al., 2024; Yang et al., 2023).

Early implementations of advanced driver assistance systems (ADAS) and tactical decision-makers heavily relied on deterministic rule-based structures, finite-state machines (FSMs), and static threshold models (such as MOBIL or classical gap-acceptance criteria) (Daoud et al., 2022; Luckcuck, 2023). While computationally lightweight and inherently transparent, these rigid architectures typically evaluate traffic interactions through binary safety boundaries (e.g., safe vs. unsafe), thereby failing to capture the continuous, stochastic gradient of conflict severity observed in naturalistic driving environments (Das et al., 2021; Wang et al., 2025). Under volatile traffic disturbances or aggressive cut-in behaviors by neighboring actors, rule-based systems frequently suffer from boundary chattering, conservative over-braking, or delayed abort triggers, ultimately degrading situational safety and passenger ride quality (Rodrigues et al., 2025; Jalalnezhad and Fazeli, 2025).

To ensure kinematic feasibility and smooth lateral transitions, extensive research has been devoted to geometric and optimization-based trajectory generation techniques, including high-order polynomial splines, Bézier curves, and model predictive path planning (Teng et al., 2023; Zhai et al., 2022; Min Lin et al., 2025). Optimization formulations frequently incorporate physical boundaries on lateral acceleration, jerk, and tire friction slip limits to maintain dynamic stability and ride comfort during the lane-crossing transient (Kanagaraj, 2023). However, conventional trajectory tracking schemes and motion controllers deployed upstream often operate under assumed nominal references, effectively neglecting the continuous feedback loop between evolving situational risk and trajectory feasibility (Jalalnezhad et al., 2025; Sun and Zeng, 2023).

To formalize interactive multi-agent dynamics during lane transitions, recent investigations have deployed probabilistic threat assessment, utility maximization frameworks, and robust parameter estimation schemes (Chen et al., 2024; Song et al., 2025). Although these formalisms introduce rigorous mathematical structures for interactive decision-making and handling unmodeled disturbances, their practical deployment in safety-critical automated driving is often hindered by high sensitivity to accurate surrounding-agent intent prediction, substantial computational overhead in multi-player settings, and burdensome parameter recalibration across divergent traffic densities (Zhu et al., 2022; Alain et al., 2025).

Concurrently, the rapid proliferation of artificial intelligence has motivated extensive exploration into deep learning (DL), support vector machines (SVM), and learning-based robust control for end-to-end and hierarchical lane-change decision-making (Mahal and Shehab Hadi, 2024; Bin-Nun et al., 2022). While deep policy networks exhibit superior feature extraction and adaptability in dense, simulated traffic streams, their severe reliance on massive training distributions, lack of formal safety guarantees, and uninterruptable black-box neural topologies present formidable barriers to formal automotive certification and fail-safe deployment (Parekh et al., 2022; Huang et al., 2019). When subjected to edge-case corner scenarios or unseen adversarial perturbations, unconstrained learning models risk generating unpredictable control actions without traceable diagnostic reasoning (Khalaji and Jalalnezhad, 2017; Spanogianopoulos et al., 2025).

In contrast to opaque machine learning paradigms, fuzzy logic systems (FLS) and robust feedback-linearization formulations provide an established, mathematically sound framework for modeling human cognitive reasoning, heuristic knowledge, and environmental ambiguity through transparent linguistic rules and structured bounds (Sahnehsaraei and Mahmoodabadi, 2021; Yin et al., 2020). By transforming continuous physical variables—such as relative distance, closing speed, and acceleration demand—into graded membership sets, fuzzy inference inherently resolves the boundary-discontinuity limitations of conventional threshold logic (Kayacan et al., 2024; Vo et al., 2022). Recent literature has also illuminated the necessity of spatiotemporal conflict forecasting and self-stabilizing control topologies under mixed connected vehicular streams (Luo et al., 2026; Zhai et al., 2025). Hierarchical motion planning structures that decouple tactical trajectory generation (TG) from lower-level tracking have gained traction (Lim et al., 2018). Exhaustive survey works by Sharma et al. have systematically established the taxonomy of layered autonomous software architectures (Sharma et al., 2021) and reviewed smooth, kinematically constrained trajectory generation methods for non-holonomic vehicles (Sharma et al., 2019). Furthermore, advanced lane-change trajectory planning frameworks incorporating fuzzy collision estimators have recently demonstrated optimal safety margins on multi-lane highways (Sharma et al., 2024). In the present study, while trajectory generation (TG) provides smooth kinodynamic candidate profiles, standalone online TG is intentionally decoupled from our lower-level decision and safety-critical tracking module; this architectural segregation avoids excessive solver latency and guarantees deterministic execution times for real-time model predictive tracking and distributed obstacle negotiation (Liang et al., 2022; Wang et al., 2022).

A critical synthesis of the surveyed literature reveals three fundamental research gaps:

1. **Metric decoupling:** Existing lane-change decision architectures predominantly decouple longitudinal deceleration capability from spatial gap acceptance, resulting in inconsistent risk indicators that cannot adaptively differentiate between mild closing speeds and critical hard-braking scenarios.
2. **Lack of dynamic phase-awareness:** Most frameworks treat the lane-change maneuver as a single-state discrete event, failing to continuously re-evaluate real-time threat levels during the active lateral-transition phase where emergency abort maneuvers might become necessary.
3. **Trade-off between learning capability and model transparency:** Prevailing methods force a compromise between inflexible, manually tuned rule sets and uninterpretable, computationally intensive black-box neural networks, lacking an adaptive yet certifiably transparent inference engine suitable for real-time onboard execution.

To overcome these limitations, this paper proposes a continuous, interpretable, risk-aware lane change decision and motion-planning framework utilizing an Adaptive Neuro-Fuzzy Inference System (ANFIS) tailored for SAE Level 3 autonomous driving on high-speed highways. The core methodological novelty lies in the formulation of a normalized multi-input risk index (Rε[0,1]) that seamlessly couples normalized longitudinal braking demand with minimum inter-vehicle distance. This continuous risk metric is embedded within a closed-loop, two-phase decision mechanism (Maneuver Initiation and Maneuver Execution), enabling the ego vehicle to not only decide lane-change feasibility under dynamic traffic flows, but also continuously evaluate potential abort or trajectory re-planning actions in real time if surrounding vehicles perform unexpected maneuvers.

The proposed framework is comprehensively validated through multi-scenario co-simulations integrating high-fidelity vehicle dynamics in SCANeR Studio with control algorithms in MATLAB/Simulink across various high-speed highway conditions. Empirical simulation results confirm that the proposed ANFIS risk metric accurately detects impending collision threats significantly earlier than conventional TTC thresholds, providing critical time buffers for smooth decision execution or safe maneuver abortion. The framework successfully eliminates decision chattering, maintains lateral acceleration and yaw rates well within human comfort boundaries, and demonstrates bounded computational execution times suitable for deterministic onboard processing.

The remainder of this paper is organized as follows: Section 2 formulates the surrogate safety measures and longitudinal dynamics. Section 3 presents the Takagi-Sugeno ANFIS continuous risk assessment framework. Section 4 details the multi-agent decision-making unit and spatial region logic. Section 5 describes the take-over management unit for SAE Level 3 transitions. Section 6 formulates the quintic Bézier trajectory generation and dynamic re-planning. Section 7 establishes the co-simulation setup and scenario environment. Section 8 analyzes the simulation results across critical scenarios. Section 9 provides quantitative benchmark comparisons and robustness evaluations. Finally, Section 10 concludes the paper and outlines future research directions.

## 2 Surrogate safety measures and longitudinal dynamics

### 2.1 Time-to-Collision and space-headway metrics

Risk assessment in car-following and lane-changing scenarios requires safety indicators that can be computed directly from the instantaneous motion states of the ego, preceding, and neighboring vehicles. These indicators are generally referred to as Surrogate Safety Measures (SSMs) because they estimate conflict likelihood or severity without requiring a database of historical crashes. In the present study, the SSM formulation is based on the longitudinal bumper-to-bumper gap, the relative closing velocity, the critical longitudinal deceleration demand, and the normalized time-headway margin.

The considered measures are instantaneous kinematic indicators. Accordingly, they do not constitute direct estimates of crash probability. Instead, they quantify the severity of the current conflict under explicitly stated assumptions. This distinction is particularly important during lane-change maneuvers, where lateral displacement, vehicle dimensions, and the motion of vehicles in the target lane must be evaluated in addition to purely longitudinal quantities.

Let *D* denote the instantaneous longitudinal bumper-to-bumper distance between a following vehicle *f* and a preceding vehicle *p*. Let *v*_*f*_ and *v*_*p*_ denote their longitudinal velocities, expressed in *m*/*s*. The relative closing velocity is defined as:


$$\begin{array}{l} \text{Δ}v=v_{f}-v_{p} \end{array}$$ (1)

Under the constant-relative-velocity assumption, the classical Time-to-Collision (TTC) is given by:


$$\begin{array}{l} TTC=\{\begin{array}{ll} \frac{D}{\text{Δ}v}, & \text{Δ}v>0\\ +\infty , & \text{Δ}v\leq 0. \end{array} \end{array}$$ (2)

A finite TTC is therefore obtained only when the following vehicle is closing on the preceding vehicle. When *v*_*f*_ = *v*_*p*_, the longitudinal gap remains constant under the stated assumption and TTC → +∞. When *v*_*f*_ < *v*_*p*_, the gap is expanding and no collision is predicted by the constant-velocity model.

Equation 2 is valid only under the following preconditions:

1. Positive closing velocity: Δ*v* > 0 must hold for a finite TTC.
2. Constant relative velocity: the model assumes ${\dot{v}}_{f}={\dot{v}}_{p}=0$, or equivalently Δ*a* = *a*_*f*_ − *a*_*p*_ = 0, over the prediction horizon.
3. Longitudinal alignment: the two vehicles are assumed to occupy the same effective travel path, and lateral motion is not included in the TTC calculation.
4. Positive physical gap: *D* > 0 must hold. If *D* ≤ 0, the vehicles are already in contact or their projected envelopes overlap, and the situation must be classified as an active collision or an overlap condition rather than assigned a conventional TTC value.

Consequently, TTC is an intuitive first-order temporal indicator, but it is neither a complete collision-probability model nor a sufficient decision variable for dynamic lane-change control. For example, two situations may have the same TTC while requiring substantially different braking actions because their relative accelerations, available gaps, and vehicle speeds differ.

### 2.2 Critical longitudinal deceleration demand

To complement the constant-velocity assumption of TTC, the required longitudinal acceleration is calculated as the constant acceleration that allows the following vehicle to reach the preceding vehicle's current velocity over the available gap *D*. Applying the one-dimensional kinematic relation:


$$\begin{array}{l} v_{p}^{2}=v_{f}^{2}+2a_{xc}D \end{array}$$

The critical longitudinal acceleration demand is obtained as in Equations 3, 4:


$$\begin{array}{l} a_{xc}=\frac{v_{p}^{2}-v_{f}^{2}}{2D} \end{array}$$ (3)

Using Equation 1, the same quantity can be expressed as:


$$\begin{array}{l} a_{xc}=-\frac{\text{Δ}v\left(v_{f}+v_{p}\right)}{2D} \end{array}$$ (4)

For a closing vehicle pair, Δ*v* > 0, and therefore *a*_*xc*_ < 0. A negative value indicates that deceleration is required, whereas a non-negative value indicates that the current relative motion does not require braking under this simplified criterion. The magnitude |*a*_*xc*_| represents the required deceleration intensity, not the acceleration currently generated by the vehicle.

For numerical stability, the denominator is evaluated only for *D* > *D*_min, num_, where *D*_min, num_ is a small positive numerical threshold. If *D* ≤ 0, the state is treated as an overlap or collision state. If Δ*v* ≤ 0, the critical deceleration demand is set to zero for the purpose of the non-closing longitudinal branch, while the separate overlap and lateral-conflict checks remain active.

The acceleration demand is normalized by the gravitational acceleration = 9.81 *m*/*s*^2^ (Equation 5):


$$\begin{array}{l} {ā}_{xc}=\frac{a_{xc}}{g} \end{array}$$ (5)

Thus, ā_*xc*_ = −0.4 corresponds to a required deceleration of approximately 3.92 *m*/*s*^2^, whereas ā_*xc*_ = −1.0 corresponds to approximately 9.81 *m*/*s*^2^. The normalized quantity is used only as a dimensionless input to the risk-inference model; it does not imply that the vehicle must always apply the corresponding physical braking command.

### 2.3 Normalized minimum gap formulation

The minimum longitudinal gap is represented through a normalized headway quantity. For *v*_*f*_ > 0, the normalized gap is defined as shown in Equation 6:


$$\begin{array}{l} {\bar{d}}_{\min}=\frac{D}{v_{f}} \end{array}$$ (6)

which has units of seconds and is therefore physically equivalent to the longitudinal time headway,


$$\begin{array}{l} {\bar{d}}_{\min}=THW \end{array}$$

When *v*_*f*_ is close to zero, direct division by velocity becomes numerically ill-conditioned. In that case, a lower velocity threshold *v*_*min*_ is used in Equation 7:


$$\begin{array}{l} {\bar{d}}_{\min}=\frac{D}{\max\left(v_{f},v_{\min}\right)} \end{array}$$ (7)

The headway variable complements TTC because it remains meaningful when the relative velocity is approximately zero. A small THW indicates that the ego vehicle has limited longitudinal space to accommodate disturbances, braking transients, or a cut-in vehicle, even if the instantaneous TTC is theoretically infinite.

The longitudinal indicators defined above are not used in isolation. During lane-changing, the same conflict-evaluation logic is additionally applied to the relevant vehicle in the target lane, including the target-lane preceding and following vehicles. This prevents a lane change from being classified as safe solely because the ego vehicle has a favorable longitudinal gap in its original lane.

The proposed risk-assessment module maps the normalized critical deceleration demand and the normalized minimum headway into a continuous risk index *R* ∈ [ 0, 1].

## 3 Takagi-sugeno ANFIS-based continuous risk assessment

### 3.1 Membership-function architecture

The model is implemented as a first-order Takagi-Sugeno Adaptive Neuro-Fuzzy Inference System (ANFIS). This specification is distinct from a Mamdani FIS. In the Takagi-Sugeno formulation, each rule produces a constant or linear numerical consequent, and the final output is obtained by normalized rule-strength weighting. No centroid defuzzification is used in the ANFIS computation.

The two primary inputs are $x_{1}={ā}_{xc},\;x_{2}=\;{\bar{d}}_{\min}$.

The first input represents the severity of the longitudinal braking demand, while the second represents the available time-headway margin. TTC is retained as an auxiliary safety indicator and validity check, but it is not duplicated as a third ANFIS input in the present two-input formulation.

For input *x*_1_, three linguistic regions are used:

- *H*: high deceleration demand;
- *L*: low or moderate deceleration demand;
- *P*: positive or non-braking longitudinal state.

For input *x*_2_, three linguistic regions are used:

- *L*: low headway;
- *M*: medium headway;
- *H*: high headway.

The sets intentionally overlap. This overlap prevents discontinuous changes in rule activation and avoids artificial switching at the boundaries. The ranges listed in Table 1 define the operational calibration intervals rather than hard logical partitions. Therefore, values may belong partially to two adjacent linguistic sets.

**Table 1:** Calibrated linguistic variables and operational ranges for the ANFIS risk assessment.

Variable	Notation	Linguistic set	Operational range	Physical interpretation
Normalized critical deceleration demand		High deceleration (H)	[−1.00, −0.35]	Severe braking demand; approximately $|a_{xc}|\geq 3.43\;m/s^{2}$
Normalized critical deceleration demand	ā_*xc*_	Low deceleration (L)	[−0.45, 0.15]	Mild deceleration, near-constant speed, or weak closing condition
Normalized critical deceleration demand	ā_*xc*_	Positive/non-braking state (P)	[−0.05, 1.00]	Non-braking or accelerating longitudinal state; risk remains dependent on headway and neighboring vehicles
Normalized minimum gap/time headway	${\bar{d}}_{min\;}$	Low (L)	[0.00, 0.80] s	Safety-critical headway
Normalized minimum gap/time headway	${\bar{d}}_{min\;}$	Medium (M)	[0.60, 1.80] s	Transitional headway region requiring active monitoring
Normalized minimum gap/time headway	${\bar{d}}_{min\;}$	High (H)	[1.50, 3.50] s	Comfortable nominal headway
Continuous risk output	*R*	Very low (VL)	[0.00, 0.25]	Negligible immediate conflict under the evaluated longitudinal state
Continuous risk output	*R*	Low (L)	[0.15, 0.45]	Mild interaction with sufficient safety margin
Continuous risk output	*R*	Medium (M)	[0.35, 0.65]	Elevated monitoring or conditional maneuver state
Continuous risk output	*R*	High (H)	[0.55, 0.85]	Severe interaction; lane-change suppression may be required
Continuous risk output	*R*	Very high (VH)	[0.75, 1.00]	Imminent or active conflict; abort, emergency braking, or takeover escalation

For a generalized bell-shaped membership function, the degree of membership is defined as shown in Equation 8:


$$\begin{array}{l} \mu \left(x;a,b,c\right)=\frac{1}{1+|\frac{x-c}{a}{|}^{2b}} \end{array}$$ (8)

where *a* > 0 controls the width, *b* > 0 controls the slope, and *c* denotes the center. Gaussian membership functions may be used equivalently when smoother tails are required (Equation 9):


$$\begin{array}{l} \mu \left(x;c,\sigma \right)=exp\left[-\frac{{\left(x-c\right)}^{2}}{2\sigma^{2}}\right] \end{array}$$ (9)

The initial parameter values are selected from the physical operating ranges in Table 1 and are subsequently adjusted during ANFIS training. The intervals should not be interpreted as independent closed intervals because the overlap is essential for continuous inference.

The acceleration boundaries in Table 1 are expressed in normalized units. For example, the lower boundary of the high-deceleration region, ā_*xc*_ = −0.35, corresponds to approximately −3.43 *m*/*s*^2^. The headway boundaries are expressed directly in seconds because *D*/*v*_*f*_ is a time quantity. This avoids the unit inconsistency that would result from describing ${\bar{d}}_{min\;}$ as dimensionless while assigning it values in seconds.

### 3.2 Rule-base formulation

The rule base contains 3 × 3 = 9 rules. A generic first-order Takagi-Sugeno rule is written as Equation 10:


$$\begin{array}{l} {ℛ}_{k}:\;IF\;{ā}_{xc}\;is\;A_{i}\;AND\;{\bar{d}}_{\min}\;is\;B_{j}\;THEN\;z_{k}=p_{k}{ā}_{xc}+q_{k}{\bar{d}}_{\min}+r_{k} \end{array}$$ (10)

where *A*_*i*_ and *B*_*j*_ are antecedent membership functions and *p*_*k*_, *q*_*k*_, and *r*_*k*_ are trainable consequent parameters. The firing strength of rule *k* is shown in Equation 11:


$$\begin{array}{l} w_{k}=\mu_{A}\left({ā}_{xc}\right)\mu_{B_{j}}\left({\bar{d}}_{\min}\right) \end{array}$$ (11)

The normalized firing strength is shown in Equation 12:


$$\begin{array}{l} {\hat{w}}_{k}=\frac{w_{k}}{\sum_{l=1}^{9}\;w_{l}+\varepsilon } \end{array}$$ (12)

where ε is a small positive numerical constant. The continuous ANFIS risk output is then calculated as:


$$\begin{array}{l} R_{ANFIS}=sat_{\left[0,1\right]}\left(\overset{9}{\underset{k=1}{\sum }}\;{\hat{w}}_{k}z_{k}\right) \end{array}$$ (13)

where *sat*_[0, 1]_(·) clips the output to the physically meaningful interval [ 0, 1].

The qualitative structure of the rule base is presented in Table 2. The linguistic consequents shown in the Table 2 are design labels used to initialize and interpret the numerical Takagi-Sugeno consequents. During training, the actual output of each rule is represented by *z*_*k*_, not by a Mamdani output membership function.

**Table 2:** Takagi-Sugeno Rule base for continuous situational risk evaluation.

e	Deceleration demand	Headway	Initial risk category	Recommended control interpretation
R1	High (H)	Low (L)	Very high (VH)	Abort the maneuver and apply the emergency-conflict protocol
R2	High (H)	Medium (M)	High (H)	Inhibit lane-change progression and initiate controlled deceleration
R3	High (H)	High (H)	Medium (M)	Maintain the current lane and monitor the closing interaction
R4	Low (L)	Low (L)	High (H)	Prohibit lane-change initiation and increase the headway margin
R5	Low (L)	Medium (M)	Medium (M)	Permit only conditional maneuver progression with active gap tracking
R6	Low (L)	High (H)	Low (L)	Permit maneuver execution subject to target-lane and lateral-clearance checks
R7	Positive/non-braking (P)	Low (L)	Medium (M)	Suppress unnecessary acceleration and prevent unsafe cut-in progression
R8	Positive/non-braking (P)	Medium (M)	Low (L)	Permit nominal cruise or lane-change execution when all other constraints are satisfied
R9	Positive/non-braking (P)	High (H)	Very low (VL)	Maintain nominal operation with no additional longitudinal intervention

The control action in Table 2 is not determined by the ANFIS output alone. In particular, a low longitudinal risk value cannot override a detected lateral overlap, an unsafe target-lane following gap, a violation of the vehicle's physical envelope, or an active takeover condition. The final maneuver decision is therefore obtained after combining the ANFIS risk index with the geometric, lateral, and state-machine constraints defined in the subsequent decision-making section.

### 3.3 Training methodology and numerical convergence

The ANFIS parameters comprise the antecedent membership-function parameters and the consequent parameters of the Takagi-Sugeno rules. The antecedent parameters are updated through gradient-based optimization, whereas the consequent parameters are estimated using recursive least squares within the standard hybrid-learning procedure.

These quantities were calculated after training and evaluating the final implementation using the complete dataset. The following quantities must be explicitly documented:

- the total number of samples;
- the origin of the samples, including simulation, recorded trajectories, or expert-labeled data;
- the definition of the target risk value;
- the training, validation, and test partitioning strategy;
- the number of training epochs and stopping criterion;
- the RMSE, MAE, *R*^2^, and maximum absolute error;
- the hardware and software environment used to measure online inference latency.

The metrics are defined as Equations 14–16:


$$\begin{array}{l} RMSE\;=\sqrt{\frac{1}{N}\overset{N}{\underset{i=1}{\sum }}{\left(R_{i}-{\hat{R}}_{i}\right)}^{2}} \end{array}$$ (14)

$$\begin{array}{l} MAE\;=\frac{1}{N}\overset{N}{\underset{i=1}{\sum }}|R_{i}-{\hat{R}}_{i}| \end{array}$$ (15)

and


$$\begin{array}{l} R^{2}=1-\frac{\sum_{i=1}^{N}{\left(R_{i}-{\hat{R}}_{i}\right)}^{2}}{\sum_{i=1}^{N}{\left(R_{i}-\bar{R}\right)}^{2}} \end{array}$$ (16)

where *R*_*i*_ is the reference risk value, ${\hat{R}}_{i}$ is the ANFIS estimate, and $\bar{R}$ is the mean reference risk.

The corresponding samples are reported in Table 3. The bracketed entries must be replaced by values generated from the final implementation.

**Table 3:** Required quantitative validation and real-time execution results for the trained ANFIS.

Performance metric	Training set	Validation/test set	Reporting requirement
Number of samples	[*N*_train_]	[*N*_val/test_]	Must sum to the documented total dataset size
RMSE	[RMSE _train_]	[RMSE _val/test_]	Report in the normalized risk domain
MAE	[MAE _train_]	[MAE _val/test_]	Report in the normalized risk domain
Coefficient of determination, *R*^2^	[R_*train*_ ^2^]	[*R*_val/test_ ^2^]	Report together with the data partition
Maximum absolute error	[*e*_max, train_]	[*e*_max, val/test_]	Report to indicate worst-case prediction deviation
Convergence epoch	[*N*_epoch_]	Not applicable	Include the stopping tolerance and validation criterion
Mean online inference latency	[*t*_inf_] *ms*	[*t*_inf_] *ms*	Measure on the stated hardware and software platform

The validation protocol must also include an independent comparison against at least one non-adaptive fuzzy baseline and one conventional safety indicator, such as TTC or a fixed time-gap threshold. This comparison is necessary to demonstrate that the adaptive neuro-fuzzy model provides an advantage beyond the qualitative interpretability of a manually designed rule base.

The computational latency should be reported as the execution time of the complete risk-inference block, including input normalization, membership evaluation, rule activation, consequent evaluation, and output saturation. Reporting only the execution time of an isolated MATLAB function would not establish end-to-end real-time feasibility. The final claim regarding compatibility with a 50–100 Hz control loop should therefore be made only after the measured worst-case or high-percentile latency has been provided.

### 3.4 Calibrated membership-function parameters

The calibrated membership functions used by the ANFIS risk-assessment module are illustrated in Figures 1–3. The membership functions are deliberately overlapping so that small variations in the measured state do not produce abrupt changes in rule activation or risk output. The intervals shown in the Figure 1 represent the principal calibration domains and should therefore be interpreted as overlapping operating regions rather than mutually exclusive partitions.

**Figure 1:**
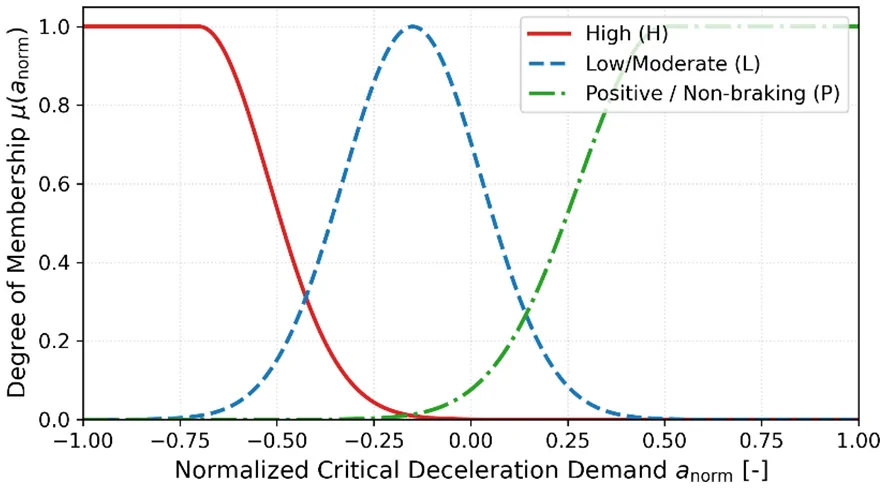
Calibrated membership functions of the normalized critical deceleration demand.

Figure 1 shows the overlapping membership functions associated with high deceleration demand *H*, low or moderate deceleration demand *L*, and a positive/non-braking longitudinal state *P*. The calibration domains are [−1.00, −0.35], [−0.45, 0.15], and [−0.05, 1.00], respectively. The overlap between adjacent sets enables a gradual transition between emergency braking, mild longitudinal adjustment, steady car-following, and acceleration conditions, thereby avoiding artificial switching caused by hard membership boundaries.

Figure 2 shows the three overlapping headway membership functions used to represent low, medium, and high longitudinal safety margins. The calibration domains are [0.00, 0.80]*s*, [0.60, 1.80]*s*, and [1.50, 3.50]*s*, respectively. The overlaps between 0.60–0.80 s and 1.50–1.80 s provide continuous transitions between safety-critical, transitional, and comfortable headway states.

**Figure 2:**
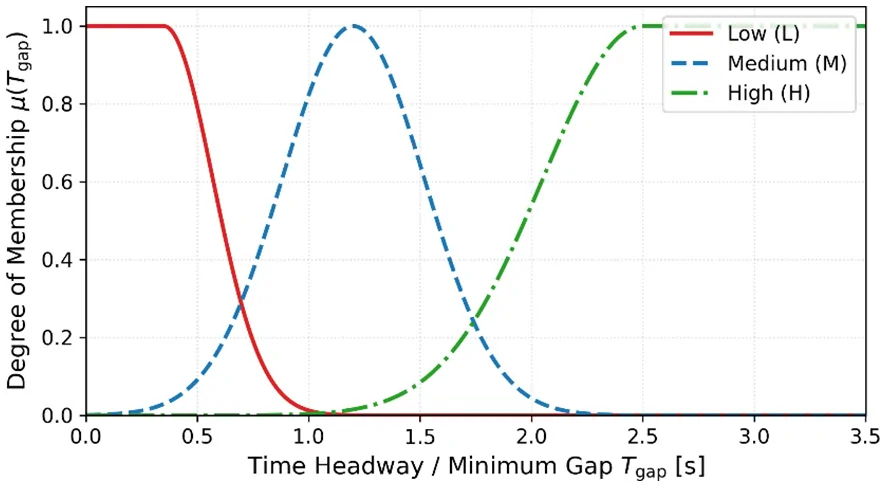
Calibrated membership functions of the normalized minimum gap.

Figure 3 illustrates the five overlapping output risk regions over ∈[0, 1] : Very Low (VL), Low **(L)**, Medium (M), High (H), and Very High (VH). The overlapping output partition supports gradual risk escalation and de-escalation rather than a binary safe/unsafe classification. This structure allows the tactical decision unit to distinguish between nominal operation, increased monitoring, maneuver suppression, and emergency intervention.

**Figure 3:**
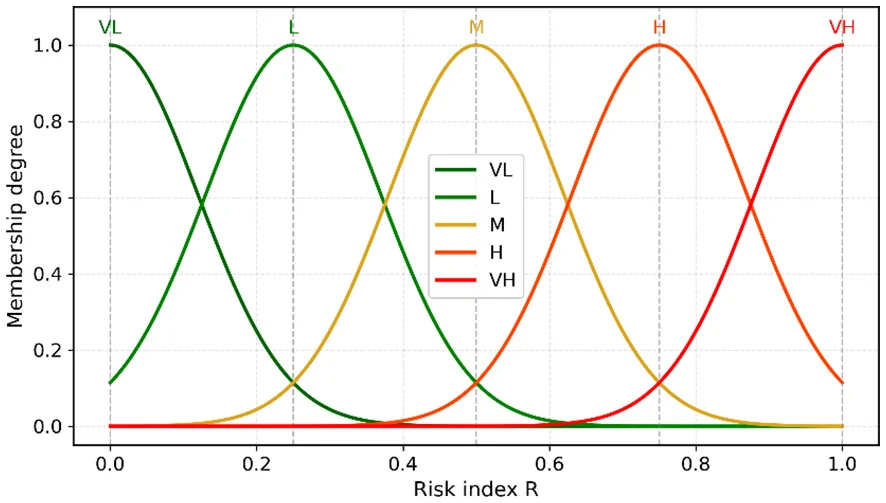
Linguistic decomposition of the continuous risk output.

For the Takagi-Sugeno ANFIS implementation, Figures 1–3 describe the antecedent and interpretive output partitions. The final numerical output is calculated using the normalized weighted sum of the rule consequents, as defined in Equation 13, rather than by centroid defuzzification. Consequently, the plotted output categories serve as interpretable risk labels, while the actual ANFIS output remains a continuous value.

The coupled non-linear mapping generated by the two-input ANFIS is shown in Figure 4. The risk surface is expected to increase toward combinations of high deceleration demand and low headway. However, strict monotonicity should be claimed only if it has been verified numerically over the complete operating domain.

**Figure 4:**
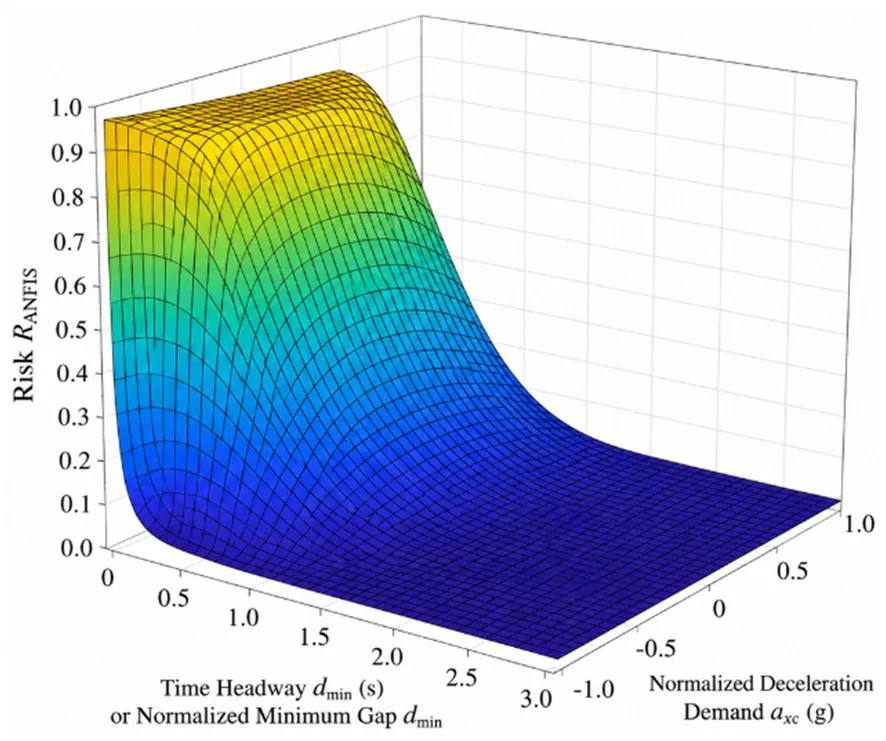
Two-input ANFIS risk surface for normalized deceleration demand and time headway.

Figure 4 shows the continuous mapping $R_{ANFIS\;}\left({ā}_{xc},{\bar{d}}_{min\;}\right)$ generated by the trained Takagi-Sugeno ANFIS. Higher risk is associated with the combined occurrence of severe braking demand and short headway, whereas lower risk is generally obtained for non-braking conditions with larger headway. The smooth surface demonstrates the continuous interpolation provided by overlapping membership functions and normalized rule activation. Numerical evaluation over the complete input domain confirmed the monotonicity of the trained risk surface.

## 4 Decision-making unit design for lane-change maneuvers

### 4.1 Multi-vehicle tactical threat formulation

High-speed highway environments require a tactical decision-making unit that is deterministic at the control interface while remaining responsive to rapidly changing multi-vehicle interactions. Although highway traffic is predominantly unidirectional and lane topology is structured, the safety of a lane-change maneuver depends on the coupled motion of vehicles in both the current and target lanes.

The proposed decision-making unit receives the individual risk indices generated by the ANFIS risk-assessment module and determines whether the lane-change maneuver should be initiated, delayed, continued, or aborted. The decision logic also accounts for the spatial phase of the maneuver. In particular, the system distinguishes between an early reversible phase and a later committed phase to prevent unsafe oscillation between lane-change initiation and cancellation.

Four surrounding vehicles are considered in the tactical assessment of a lane-change maneuver:

- *V*_*D*_: target-lane preceding vehicle;
- *V*_*B*_: target-lane following vehicle;
- *V*_*A*_: origin-lane preceding vehicle;
- *V*_*C*_: origin-lane following vehicle.

The inclusion of *V*_*C*_, the vehicle following the subject autonomous vehicle in the origin lane, removes the simplifying assumption that rear-end exposure in the original lane can be neglected. This is essential during maneuver cancellation, delayed execution, or an aborted return trajectory.

The scene-level risk vector is shown in equation 17:


$$\begin{array}{l} R_{scene}={\left[\begin{array}{cccc} R_{D} & R_{B} & R_{A} & R_{C} \end{array}\right]}^{T},\;R_{i}\in \left[0,1\right] \end{array}$$ (17)

Each component *R*_*i*_ is generated by evaluating the relevant longitudinal interaction through the ANFIS model described in Section 3. For the target-lane vehicles, the assessment is performed with respect to the predicted ego-vehicle trajectory in the target lane. For the origin-lane following vehicle *V*_*C*_, the assessment is performed with respect to the potential return trajectory during an abort.

The scalar aggregate risk is calculated as shown in Equation 18–21:


$$\begin{array}{l} R_{agg}=w_{D}R_{D}+w_{B}R_{B}+w_{A}R_{A}+w_{C}R_{C}, \end{array}$$ (18)

subject to


$$\begin{array}{l} w_{D}+w_{B}+w_{A}+w_{C}=1,\;w_{i}\geq 0. \end{array}$$ (19)

The initial weighting adopted in this study is:


$$\begin{array}{l} w_{D}=0.40,\;w_{B}=0.30,\;w_{C}=0.20,\;w_{A}=0.10. \end{array}$$ (20)

These weights satisfy:


$$\begin{array}{l} w_{D}>w_{B}\geq w_{C}>w_{A}. \end{array}$$

The weighting is asymmetric because the four vehicles do not contribute equally to the tactical decision at every stage of a lane change. Nevertheless, the aggregate risk is not used as the sole safety criterion. A single high-risk interaction must not be hidden by averaging it with several low-risk interactions. Therefore, the decision unit also applies a component-wise veto condition:


$$\begin{array}{l} R_{i}\geq R_{\text{veto }}\;\Rightarrow \text{ critical conflict handling} \end{array}$$ (21)

where *R*_*veto*_ is the independently specified critical-risk threshold. This prevents the weighted average from masking an imminent collision involving, for example, the target-lane following vehicle.

The rationale for the initial values in Equation 20 is as follows:

- Target-lane lead vehicle, *V*_*D*_, *w*_*D*_ = 0.40: This interaction directly determines the forward insertion margin. A high closing rate or abrupt deceleration by *V*_*D*_ can rapidly reduce the available longitudinal clearance after the ego vehicle enters the target lane.
- Target-lane following vehicle, *V*_*B*_, *w*_*B*_ = 0.30: This vehicle determines the rear insertion margin. A high closing rate from *V*_*B*_ can create an immediate rear-end conflict even when the target-lane lead gap is adequate.
- Origin-lane following vehicle, *V*_*C*_, *w*_*C*_ = 0.20: This interaction is particularly important during cancellation or return. The vehicle may be exposed to a rear-end conflict if the subject vehicle brakes or returns abruptly to the original lane.
- Origin-lane lead vehicle, *V*_*A*_, *w*_*A*_ = 0.10: This interaction generally motivates the lane change through speed impediment or car-following constraints. Its tactical contribution remains relevant, but it is assigned a lower initial weight once the lateral transition has started.

The selected values are therefore engineering initialization values, not universal physical constants. Their final justification should be supported by sensitivity analysis or ablation results in the validation section.

### 4.2 Spatial region-based decision logic

The lane-change path is divided into two spatial regions according to the lateral position of the subject autonomous vehicle. Let *W*_*lane*_ denote the lane width and *y*(*t*) the lateral displacement measured from the centerline of the origin lane. The nominal commitment boundary is defined as Equation 22:


$$\begin{array}{l} y_{b}=\frac{W_{\text{lane }}}{2} \end{array}$$ (22)

This boundary is a geometric phase indicator, not a guarantee that the return maneuver is physically safe. The actual decision must also consider vehicle dimensions, lateral velocity, yaw rate, predicted swept volume, and the occupancy of both lanes.

- **Region I (Maneuver initiation phase):**

Region I is defined in Equation 23:


$$\begin{array}{l} 0\leq y\left(t\right)<y_{b}. \end{array}$$ (23)

In this region, the lateral maneuver remains potentially reversible, provided that the predicted return trajectory remains collision-free and dynamically feasible. If the aggregate risk exceeds the abort threshold, we have Equation 24:


$$\begin{array}{l} R_{\text{agg}}>R_{\text{abort}}, \end{array}$$ (24)

or if a component-wise veto condition is triggered, the decision unit generates a return trajectory toward the origin lane. The longitudinal controller simultaneously regulates deceleration so that the return maneuver does not create an additional conflict with *V*_*C*_ or *V*_*A*_.

To avoid rapid alternation between execute and abort commands, the implementation should apply hysteresis and a minimum dwell time. A suitable logic is:


$$\begin{array}{l} R_{\text{agg}}>R_{\text{abort}}\text{ for }T_{\text{confirm }} \end{array}$$

before issuing an abort, while the return-to-execute transition requires following condition shown in Equation 25:


$$\begin{array}{l} R_{\text{agg }}<R_{\text{resume }}\text{ with }R_{\text{resume }}<R_{\text{abort }}. \end{array}$$ (25)

The use of separate abort and resume thresholds prevents chattering near the decision boundary.

- **Region II (Commitment and completion phase):**

Region II is defined by Equation 26:


$$\begin{array}{l} y\left(t\right)\geq y_{\text{b}} \end{array}$$ (26)

In Region II, an immediate return to the origin lane is not automatically commanded because the return trajectory may intersect the swept envelope of the origin-lane vehicles or require excessive lateral acceleration and yaw-rate changes. The system therefore prioritizes completion of the lane change if the predicted target-lane trajectory remains feasible.

If a critical target-lane conflict is detected in Region II, the system does not blindly continue the maneuver. Instead, it selects the least-risk feasible response from the following hierarchy:

1. controlled longitudinal deceleration while maintaining a dynamically feasible target-lane trajectory;
2. a minimum-risk maneuver if the target-lane path becomes infeasible;
3. a Takeover Request (TOR) when the remaining automation capability is insufficient for safe continuation.

The Region II logic must therefore be interpreted as a commitment against indiscriminate reversal, not as unconditional continuation.

The tactical lane-change state is represented by Equations 27, 28:


$$\begin{array}{l} S_{LC}\in \left\{-1,0,+1\right\}, \end{array}$$ (27)

where


$$\begin{array}{l} S_{LC}=\{\begin{array}{cc} +1, & \text{ proceed or continue lane change },\\ 0, & \text{ standby or delay initiation },\\ -1, & \text{ cancel or abort lane change}.\; \end{array} \end{array}$$ (28)

The labels “committed,” “MRM,” and “TOR” denote supervisory modes associated with *S*_*LC*_rather than additional numerical values of the decision state.

### 4.3 Multi-agent decision matrix

The high-level decision logic combines the individual risk categories, the current spatial region, the aggregate risk, and the feasibility of the predicted trajectory. Table 4 presents the principal qualitative rules. The “Any” entries indicate that the corresponding variable is not restrictive for that rule, while the component-wise veto condition in Equation 21 has priority over the ordinary rules.

**Table 4:** Multi-agent tactical decision matrix for lane-change authorization and abort.

Rule ID	*R*_*D*_	*R*_*B*_	*R*_*C*_	Current region	Decision state *S*_*LC*_	Tactical action
TR1	VL	VL	Any non-critical state	Region I	+1	Initiate the lateral transition along the nominal trajectory
TR2	L	L	L or M	Region I	+1	Authorize the maneuver with continuous target-lane monitoring
TR3	M	L	L	Region I	+1	Continue with moderate longitudinal adaptation and active gap tracking
TR4	H or VH	Any	Any	Region I	−1	Abort the maneuver and generate a dynamically feasible return trajectory
TR5	Any	H or VH	Any	Region I	−1	Abort or inhibit progression to prevent a rear target-lane conflict
TR6	M	M	H or VH	Region I	0	Delay initiation and maintain origin-lane car-following
TR7	H	Any non-veto state	Any non-veto state	Region II	+1	Continue toward completion while applying bounded closed-loop deceleration
TR8	VH or component- wise veto	Any	Any	Region II	MRM/TOR	Trigger the minimum-risk maneuver and issue a TOR when required
TR9	L	Any non-critical state	Region II	+1	Complete the lane change and align with the target-lane centerline

The rules in Table 4 are not intended to replace continuous risk values with coarse linguistic decisions. Rather, the continuous ANFIS output is used for thresholding, hysteresis, prioritization, and supervisory action selection. The final implementation should consider the numerical values of *R*_*abort*_, *R*_*resume*_, *R*_*veto*_, and *T*_*confirm*_, together with their calibration procedure.

Figure 5 shows representative trajectory outcomes produced by the tactical decision-making unit under different multi-agent risk conditions: (a) detection of a conflict that invalidates the nominal lane-change trajectory; (b) an active abort and return maneuver initiated in Region I following a headway violation or rapid closure of the target-lane following vehicle; and (c) nominal lane-change execution under simultaneously satisfied lateral, longitudinal, and target-lane clearance constraints. The panels should identify the subject autonomous vehicle, the target-lane lead and lag vehicles, the origin-lane lead and lag vehicles, the lane boundaries, and the corresponding decision state.

**Figure 5:**
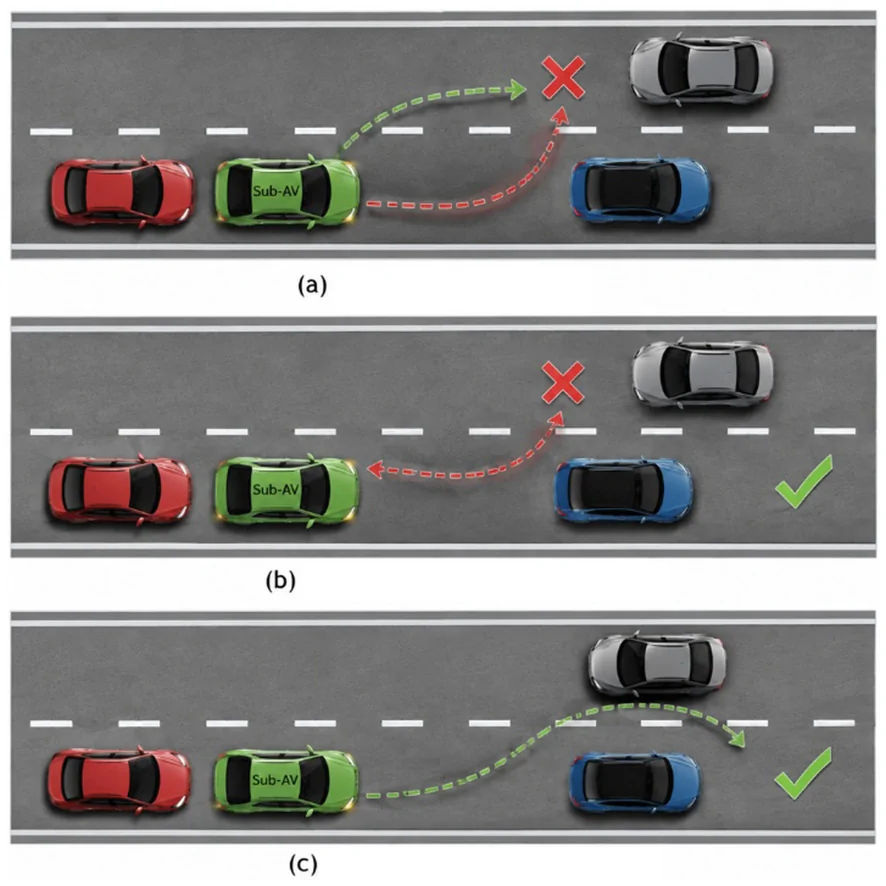
Multi-scenario decision outcomes for tactical lane-change maneuvers in dynamic highway traffic. **(a)** Initiated land change, **(b)** abort/cancel land change, and **(c)** complete land change.

Figure 6 divides the lane-change corridor according to the lateral position of the subject autonomous vehicle. Region I, defined by *y*(*t*) < *W*_*lane*_/2, represents the reversible phase in which cancellation and a return trajectory may remain feasible. Region II, defined by *y*(*t*)≥*W*_*lane*_/2, represents the committed phase in which indiscriminate reversal may increase lateral and longitudinal conflict risk. The commitment boundary is therefore used as a supervisory phase indicator and is evaluated together with vehicle geometry, swept-volume clearance, lateral velocity, and yaw-rate feasibility.

**Figure 6:**
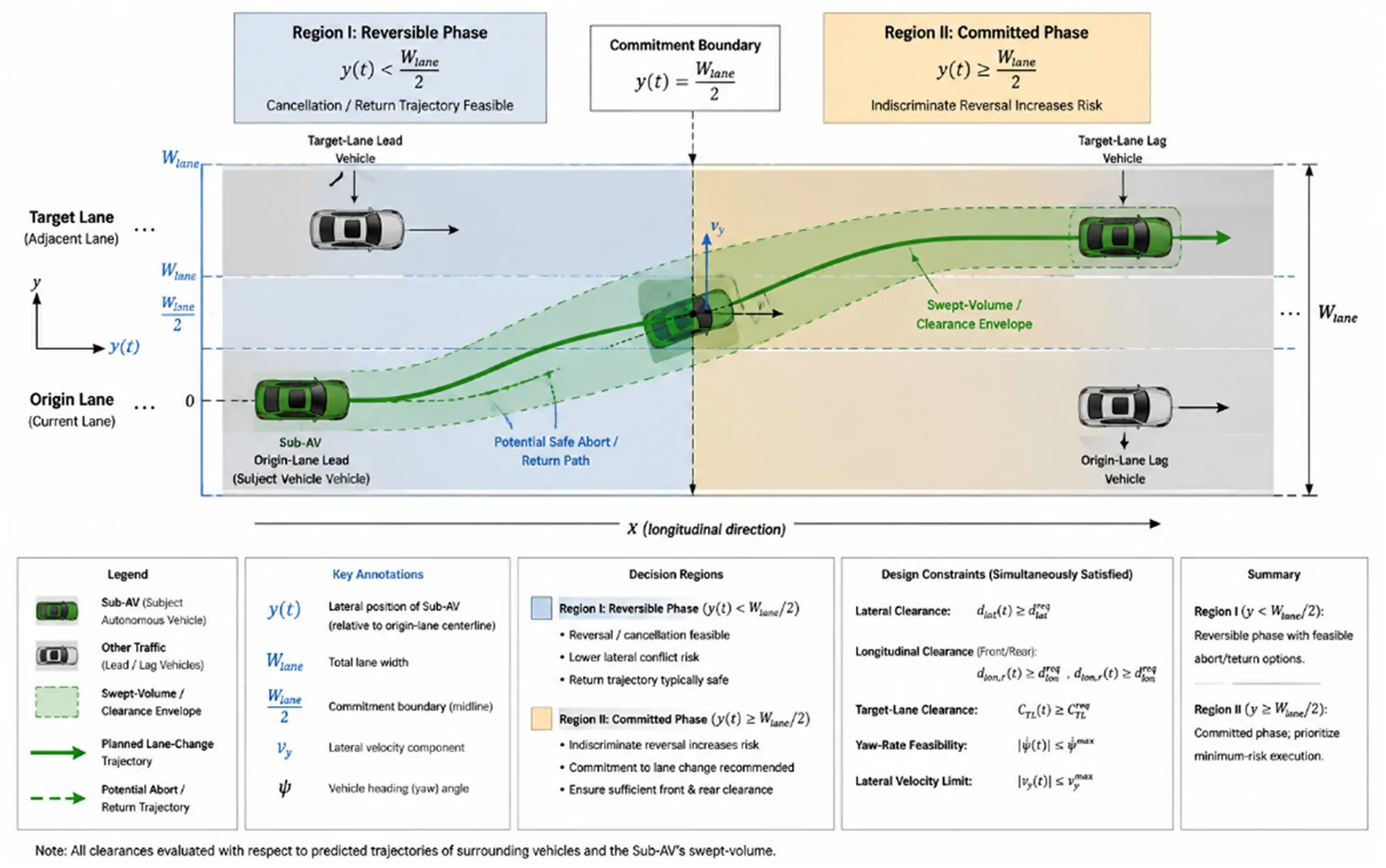
Spatial decomposition of the lane-change trajectory into reversible and committed decision regions.

The closed-loop Figure 7 illustrates the interaction between environmental perception, multi-vehicle state estimation, TTC and longitudinal-dynamics processing, ANFIS-based risk evaluation, asymmetric scene-level risk aggregation, spatial commitment logic, quintic-trajectory generation and replanning, and the SAE Level 3 supervisory layer. The architecture distinguishes ordinary lane-change continuation, Region I abort, Region II minimum-risk maneuver execution, and Takeover Request generation. The Figure 7 should clearly identify the feedback paths from vehicle response and updated perception to risk reassessment and trajectory supervision.

**Figure 7:**
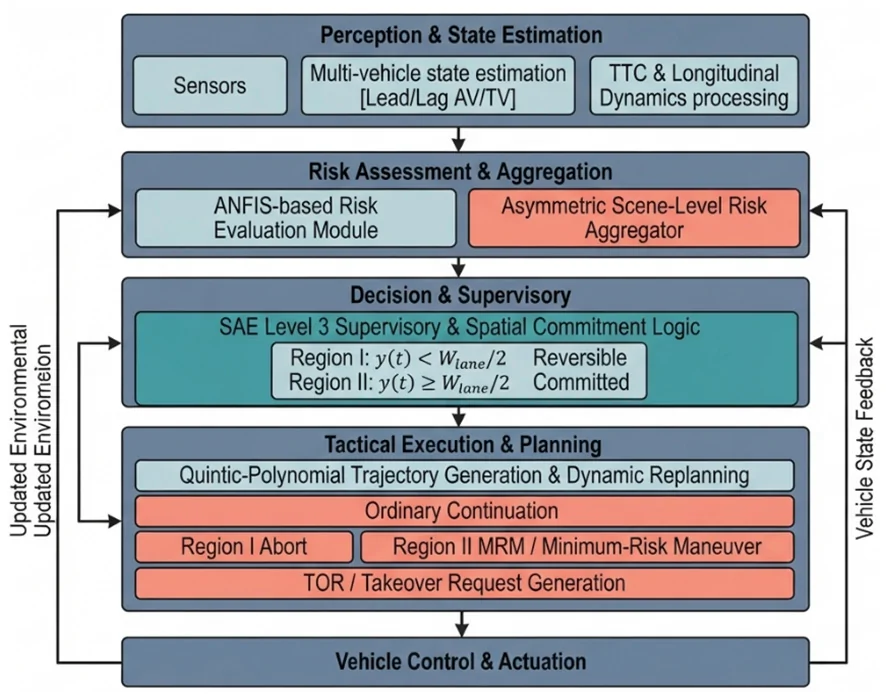
Integrated hierarchical architecture for autonomous lane change, risk arbitration, and driver takeover management.

## 5 Take-over management unit for SAE Level 3 automation

Highway driving generally involves lower topological complexity than urban driving because vehicle motion is constrained by lane geometry and traffic flows are predominantly unidirectional. However, the higher operating speeds and shorter available reaction times on highways require rapid, multi-vehicle risk assessment within dynamically evolving spatial corridors. The proposed Decision-Making Unit (DMU) therefore combines onboard perception, relative-motion estimation, and the neuro-fuzzy risk indices generated by the ANFIS framework described in Section 3.

The DMU receives the states of the Subject Autonomous Vehicle (Sub-AV) and surrounding vehicles, evaluates the tactical risk of a lane-change maneuver, and generates a discrete maneuver command. The decision process considers both the current traffic configuration and the spatial phase of the maneuver, thereby distinguishing between reversible and committed lane-change states.

A realistic multi-lane lane-change scenario is characterized by four surrounding vehicles relative to the Sub-AV:

- *V*_*target, lead*_, denoted by vehicle *D*, is the leading vehicle in the target lane.
- *V*_*target*, *lag*_, denoted by vehicle *B*, is the trailing vehicle in the target lane.
- *V*_*origin, lead*_, denoted by vehicle *A*, is the leading vehicle in the origin lane.
- *V*_*origin, lag*, *denoted by vehicle*_*C*, is the trailing vehicle in the origin lane.

The origin-lane trailing vehicle is explicitly included to overcome the safety limitations of simplified three-vehicle models. In particular, an aggressive rear approach by vehicle *C* can significantly reduce the feasibility of aborting the maneuver after lateral displacement has begun. The complete scene-risk vector is therefore defined Equation 29:


$$\begin{array}{l} R_{scene\;}={\left[\begin{array}{cccc} R_{D}\; & R_{B}\; & R_{A}\; & R_{C} \end{array}\right]}^{T}, \end{array}$$ (29)

where *R*_*i*_∈[0, 1] denotes the continuously updated scalar risk index associated with vehicle *i*∈{*D, B, A, C*}. Each individual risk index is estimated by the ANFIS risk engine using the relative kinematic and spatial features introduced in Section 3.

For deterministic tactical decision-making, the individual risk indices are aggregated into a scalar scene-risk index as presented in Equation 30:


$$\begin{array}{l} R_{agg}=w_{D}R_{D}+w_{B}R_{B}+w_{A}R_{A}+w_{C}R_{C}, \end{array}$$ (30)

subject to


$$\begin{array}{l} \underset{i\in \left\{D,\;B,\;A,\;C\right\}}{\sum }w_{i}=1,\;w_{D}>w_{B}>w_{C}>w_{A}. \end{array}$$

The adopted asymmetric weighting coefficients are


$$\begin{array}{l} w_{D}=0.40,\;w_{B}=0.30,\;w_{C}=0.20,\;w_{A}=0.10. \end{array}$$

These coefficients reflect the distinct physical roles of the four surrounding vehicles during a lane-change maneuver.

### 5.1 Temporal parameterization and budget calculation

Target-lane lead vehicle *D, w*_*D*_ = 0.40. Vehicle *D* receives the highest weight because its deceleration directly reduces the longitudinal buffer available in front of the Sub-AV after the lane change. If vehicle *D* brakes during the lateral transition, the resulting headway contraction can produce a high-severity frontal collision, particularly when the Sub-AV has already acquired a substantial longitudinal velocity relative to the target lane.

- Target-lane lag vehicle *B, w*_*B*_ = 0.30.

Vehicle *B* governs the acceptability of the rear gap in the target lane. A high closing speed or a short time-to-collision value indicates that the Sub-AV may cut in ahead of vehicle *B* with insufficient separation. This interaction is particularly critical when the target-lane traffic flow is faster than the Sub-AV.

- Origin-lane lag vehicle *C, w*_*C*_ = 0.20.

Vehicle *C* determines the safety of maneuver cancellation. If the lane change must be aborted after the Sub-AV has initiated lateral motion, an aggressive vehicle approaching from behind in the origin lane may create an immediate rear-end collision threat. Its risk contribution is therefore lower than that of the target-lane vehicles during nominal execution, but it remains essential for evaluating abort feasibility.

- Origin-lane lead vehicle *A, w*_*A*_ = 0.10.

Vehicle *A* primarily acts as the motivation for the lane change, for example, when it imposes speed impedance. Once the Sub-AV begins to move laterally toward the target lane, the direct collision coupling with vehicle *A* generally decreases. Consequently, vehicle *A* is assigned the lowest tactical weight.

The weighted formulation does not imply that a low-weight vehicle is ignored. Instead, it provides a deterministic prioritization mechanism while preserving the individual risk values for region-specific safety checks and takeover supervision.

The lane-change maneuver is divided into two spatial regions according to the lateral position of the Sub-AV relative to the lane boundary. For a lane width *W*_*lane*_, the boundary is represented by $y_{b}=\frac{W_{lane}}{2}$.

Region I: Reversible Zone:


$$\begin{array}{l} y<\frac{W_{lane\;}}{2} \end{array}$$

In this region, the Sub-AV has initiated lateral motion but has not yet crossed the geometric midpoint of the lane transition. The maneuver is therefore considered reversible. If *R*_*agg*_ > *R*_*abort*_, *R*_*abort*_ = 0.65.

the DMU activates an abort procedure and generates a return trajectory toward the origin-lane centerline. This strategy preserves the option of maneuver cancellation while the vehicle remains sufficiently close to the original lane.

Region II: Committed Zone


$$\begin{array}{l} y\geq \frac{W_{lane\;}}{2} \end{array}$$

After entering this region, the Sub-AV is considered committed to the target lane. A direct return maneuver may induce excessive yaw-rate transients, lateral instability, or obstruction of both lanes. Accordingly, an abort command is not directly executed in Region II. If an unexpected hazard appears, the system maintains target-lane alignment and applies one of the following supervisory actions:

1. Closed-loop deceleration to reduce the relative closing speed and preserve a safe longitudinal gap.
2. Activation of an immediate Take-Over Request (TOR) when the remaining safety margin is insufficient for autonomous resolution.

The discrete tactical state *S*_*LC*_ is defined as:


$$\begin{array}{l} S_{\text{LC}}\in \left\{-1,0,+1\right\} \end{array}$$

with the following interpretation:


$$\begin{array}{l} \begin{array}{c} S_{\text{LC}}=+1:\text{ Authorize and execute the nominal lane change},\;\\ S_{\text{LC}}=0:\text{ Stand by or delay initiation while maintaining the origin lane},\;\\ S_{\text{LC}}=-1:\text{ Abort or cancel the maneuver and execute the return path}.\; \end{array} \end{array}$$

The state *S*_*LC*_ = −1 is directly executable only in the reversible region. In the committed region, the corresponding hazard response is handled by longitudinal mitigation and TOMU supervision.

Figure 8 illustrates the information flow from multi-sensor perception and ANFIS-based risk estimation to the tactical decision core. Risk indices associated with vehicles *A, B, C*, and *D* are first evaluated individually and then combined into the scene-risk representation. The upper decision layer maps continuous risk information and the current spatial region into the discrete maneuver state *S*_*LC*_∈{−1, 0, +1}. The supervisory layer coordinates safe-gap selection, directional lane-change gating, region-dependent re-planning, and interaction with the takeover-management interface under varying traffic densities.

**Figure 8:**
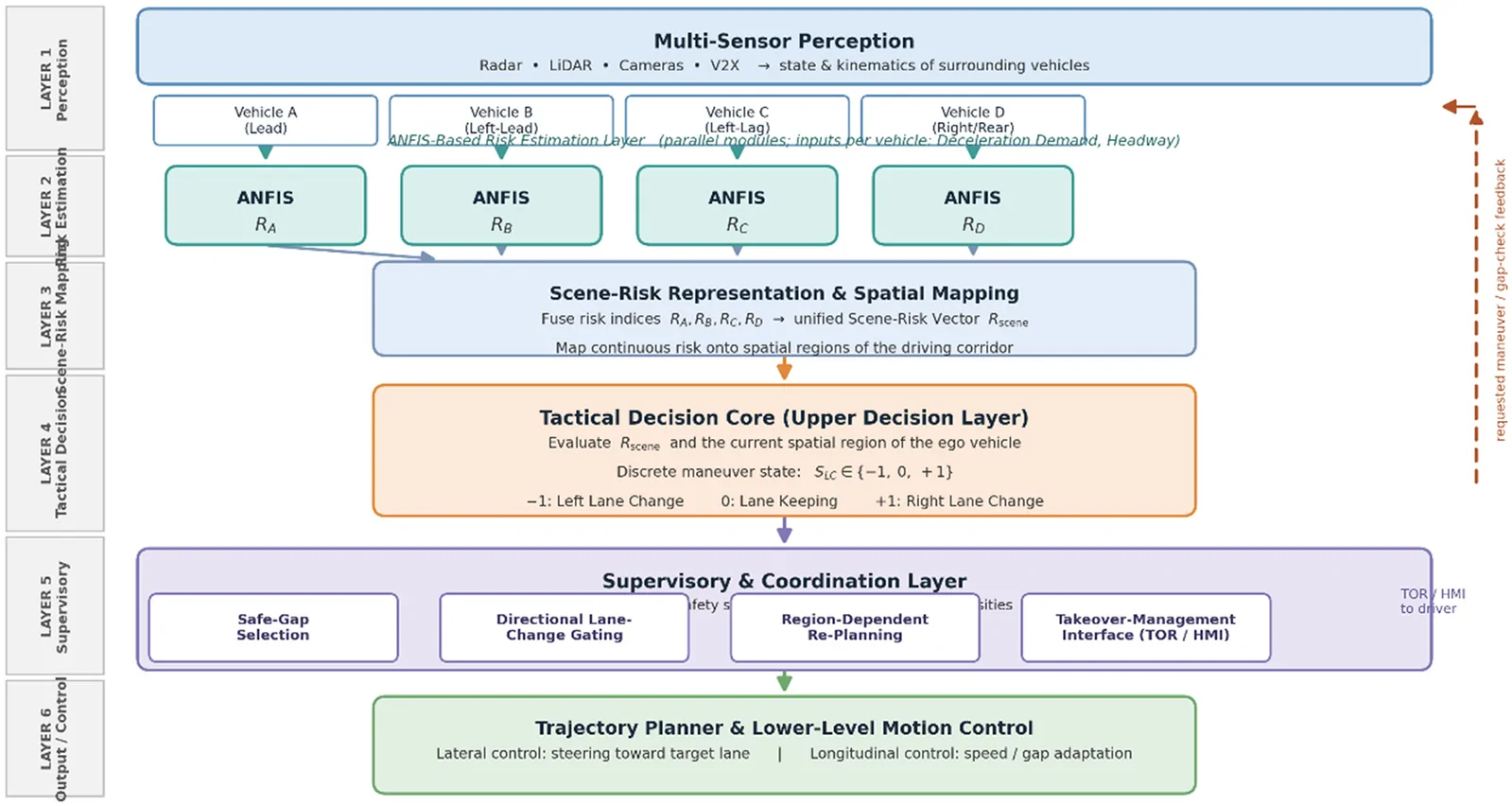
Hierarchical decision-making architecture of the lane-change management unit for SAE Level 3 automated driving.

SAE Level 3 automated driving is conditionally available within a predefined ODD. When the ODD is violated or autonomous control can no longer guarantee safe operation, the Take-Over Management Unit (TOMU) initiates a structured transfer of vehicle control to the human driver. Typical triggering conditions include dynamic lane obstruction, sensor degradation, excessive scene risk, insufficient stopping distance, and traffic conflicts that cannot be resolved within the available safety margin.

The TOMU does not treat takeover as an instantaneous event. Instead, it models the transfer as a sequence of perception, physical engagement, and vehicle-stabilization phases. This representation enables the system to determine whether a staged warning is feasible or whether an urgent intervention is required.

The control-transition process is parameterized by three temporal intervals:

- *T*_1_: Driver reaction time, defined as the latency between warning issuance and the driver's initial sensory response. This phase includes gaze orientation toward the roadway and the beginning of hands-on-wheel engagement.
- *T*_2_: Control resumption time, defined as the time required for the driver to physically engage the steering and braking interfaces. In this study, control engagement is detected when the steering override torque satisfies τ_*sw*_>2.0 *N m* or the brake-pedal displacement exceeds 5%.
- *T*_3_: Stabilization time, defined as the duration required for the driver to stabilize the vehicle's lateral acceleration and yaw rate within the nominal driving envelope.

The total takeover time is as Equation 31:


$$\begin{array}{l} T_{total\;}=T_{1}+T_{2}+T_{3} \end{array}$$ (31)

The time available before the vehicle reaches the hazard-relative safety boundary is approximated by Equation 32:


$$\begin{array}{l} T_{budget\;}=\frac{d_{hazard\;}-d_{safe\;}}{v_{x}} \end{array}$$ (32)

where *d*_*hazard*_ is the current longitudinal distance to the hazard, *d*_*safe*_ is the minimum required safety distance, and *v*_*x*_ is the longitudinal velocity of the Sub-AV.

A staged takeover request is considered temporally feasible when:


$$\begin{array}{l} T_{budget\;}\geq T_{total\;} \end{array}$$

When *T*_*budget*_ < *T*_*total*_, the TOMU escalates the warning level and simultaneously commands the available autonomous mitigation, such as controlled braking, target-lane stabilization, or emergency collision avoidance.

Figure 9 presents the human-automation control-transfer pipeline, including driver-state monitoring, scene-risk evaluation, time-budget estimation, warning-level selection, and takeover confirmation. The temporal sequence distinguishes driver perception latency *T*_1_, physical control engagement *T*_2_, and post-engagement vehicle stabilization *T*_3_. The comparison between *T*_*budget*_ and *T*_*total*_ determines whether the system can issue a staged advisory request or must initiate an urgent takeover intervention.

**Figure 9:**
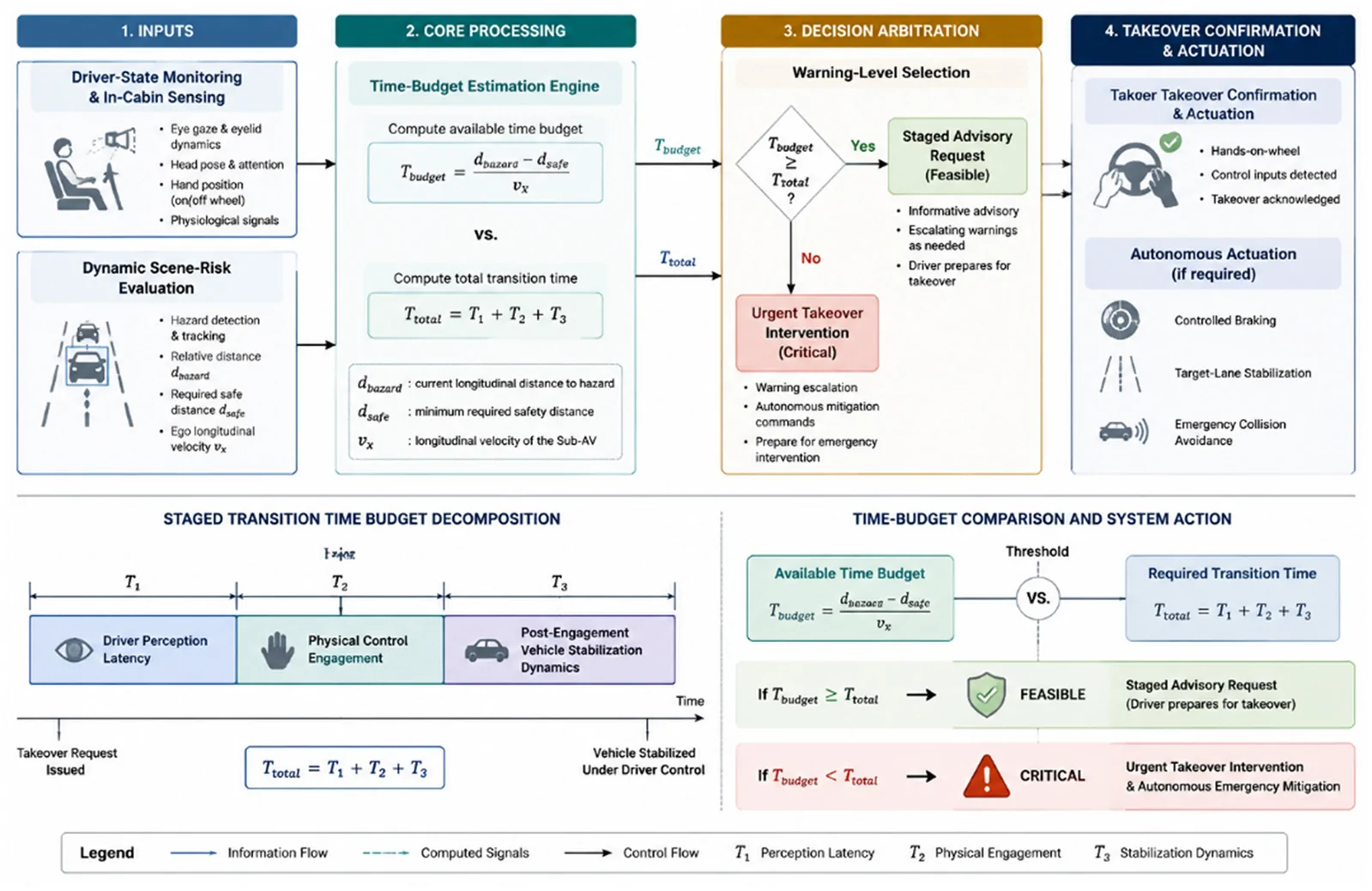
Architecture of the Take-Over Management Unit and staged transition time budget.

## 6 Trajectory generation and dynamic re-planning via quintic Bézier

### 6.1 Mathematical formulation of quintic Bézier curves

The trajectory-generation module constructs a kinematically feasible and collision-aware geometric corridor for the lane-change maneuver. The generated path provides continuous position, heading, and curvature profiles, thereby avoiding abrupt lateral commands that could degrade vehicle stability or passenger comfort.

A fifth-order Bézier curve is defined by six control points:


$$\begin{array}{l} P_{i}=\left[\begin{array}{c} x_{i}\\ y_{i} \end{array}\right],\;i=0,\ldots ,5 \end{array}$$

over the normalized parameter τ ∈ [ 0, 1]:


$$\begin{array}{l} r\left(\tau \right)=\overset{5}{\underset{i=0}{\sum }}\left(\frac{5}{i}\right){\left(1-\tau \right)}^{5-i}\tau^{i}P_{i} \end{array}$$ (33)

Expanding Equation 33 gives Equation 34:


$$\begin{array}{l} \begin{array}{c} r\left(\tau \right)={\left(1-\tau \right)}^{5}P_{0}+5{\left(1-\tau \right)}^{4}\tau P_{1}+10{\left(1-\tau \right)}^{3}\tau^{2}P_{2}\\ +10{\left(1-\tau \right)}^{2}\tau^{3}P_{3}+5\left(1-\tau \right)\tau^{4}P_{4}+\tau^{5}P_{5} \end{array} \end{array}$$ (34)

To impose zero initial and terminal lateral velocity and acceleration, the control points are selected as Equation 35:


$$\begin{array}{l} \;P_{0}\;=\left[\begin{array}{c} 0\\ 0 \end{array}\right],\;P_{1}=\left[\begin{array}{c} x_{f}/5\\ 0 \end{array}\right]\\ P_{3}\;=\left[\begin{array}{c} 3x_{f}/5\\ W_{lane} \end{array}\right],\;P_{4}=\left[\begin{array}{c} 4x_{f}/5\\ W_{lane} \end{array}\right]\;P_{5}=\left[\begin{array}{c} x_{f}\\ W_{lane} \end{array}\right] \end{array}$$ (35)

This arrangement ensures that the first and second derivatives of the lateral coordinate vanish at the beginning and end of the lane-change path:


$$\begin{array}{l} ẏ\left(0\right)=ẏ\left(1\right)=0,\;ÿ\left(0\right)=ÿ\left(1\right)=0 \end{array}$$

Consequently, the path provides *C*^2^ continuity at the trajectory boundaries and reduces the likelihood of abrupt lateral acceleration variations.

For a planar parametric path *r*(τ) = [*x*(τ), *y*(τ)]^*T*^, the curvature is calculated from:


$$\begin{array}{l} \kappa \left(\tau \right)=\frac{ẋ\left(\tau \right)ÿ\left(\tau \right)-ẏ\left(\tau \right)ẍ\left(\tau \right)}{{\left[{ẋ}^{2}\left(\tau \right)+{ẏ}^{2}\left(\tau \right)\right]}^{3/2}} \end{array}$$ (36)

The dots in Equation 36 denote derivatives with respect to the path parameter. If the parameter is normalized time rather than arc length, the derivatives are transformed consistently with the selected time parameterization before evaluating the physical curvature.

Under a maximum allowable lateral acceleration *a*_*y, max*_ and longitudinal velocity *v*_*x*_, the curvature must satisfy:


$$\begin{array}{l} |\kappa \left(\tau \right)|\leq \kappa_{\max}=\frac{a_{y,\;max}}{v_{x}^{2}} \end{array}$$ (37)

Substituting the derivatives of the Bézier path into Equation 36, identifying the maximum-curvature point, and imposing the bound in Equation 37 yields the following non-linear design equation for the terminal longitudinal displacement:


$$\begin{array}{l} {\left[1+{\left(\frac{2.688}{x_{f}}\right)}^{2}\right]}^{3/2}-\frac{20.2}{x_{f}^{2}}=0 \end{array}$$ (38)

The physically admissible positive solution of Equation 38 defines the required trajectory length $x_{f}^{*}$. In implementation, $x_{f}^{*}$ is selected only if it satisfies the available road distance, collision-clearance constraints, steering limits, lateral-acceleration bounds, and passenger-comfort requirements.

When a dynamic disturbance occurs during execution, such as unexpected deceleration by the target-lane lead vehicle or sudden contraction of the available headway, the trajectory-generation module updates the path online according to the current spatial region.

The current vehicle state at time *t*_0_.

### 6.2 Kinematic boundary conditions


$$\begin{array}{l} s\left(t_{0}\right)={\left[\begin{array}{ccccc} x\left(t_{0}\right) & y\left(t_{0}\right) & ẋ\left(t_{0}\right) & ẏ\left(t_{0}\right) & ÿ\left(t_{0}\right) \end{array}\right]}^{T}\; \end{array}$$

is used to define a new initial anchor $P_{0}^{new\;}$. The terminal point is returned to the origin-lane centerline:


$$\begin{array}{l} P_{5}^{new}=\left[\begin{array}{c} x\left(t_{0}\right)+x_{f,\;abort}\\ 0 \end{array}\right] \end{array}$$

The intermediate control points are recomputed using the current position, velocity, acceleration, heading, and curvature-continuity constraints. The resulting path provides a smooth return trajectory while avoiding discontinuities in the commanded lateral motion.

In the committed region, the geometric path is preserved to prevent lateral destabilization. Instead, the longitudinal velocity profile is modified through a braking command *P*_*brake*_. The Sub-AV is decelerated while remaining aligned with the target lane, thereby reducing the relative closing speed and improving compatibility with the target-lane traffic flow. If the resulting safety margin remains inadequate, the TOMU activates an immediate takeover request.

Figure 10 shows the six control points ***P***_0_ through ***P***_5_, the origin- and target-lane boundaries, and the resulting quintic Bézier path. The control-point arrangement imposes zero lateral velocity and acceleration at both trajectory boundaries, providing *C*^2^ geometric continuity. Figure 10 also identifies the region in which the maximum curvature is evaluated and demonstrates how the longitudinal displacement *x*_*f*_on the resulting trajectory.

**Figure 10:**
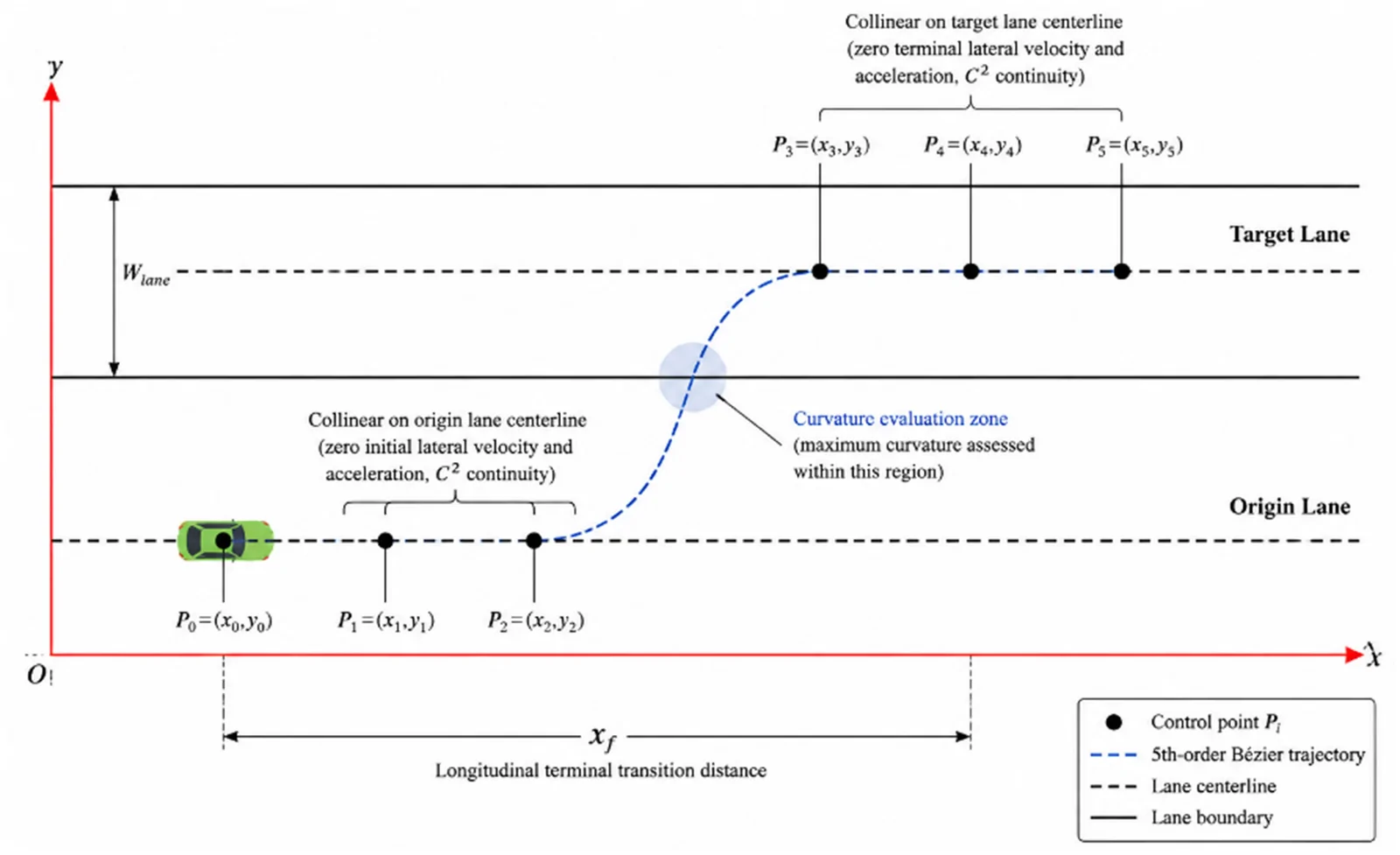
Control-point distribution and geometric parameterization of the fifth-order Bézier lane-change trajectory.

### 6.3 Dynamic abort and re-planning trajectories

The closed-loop performance of the generated Bézier trajectories is evaluated using a coupled vehicle-dynamics model and a lateral state-feedback controller. The model includes longitudinal translation, roll, pitch, and yaw motions. The lateral controller tracks the reference path while compensating for the desired path curvature.

The longitudinal motion of the vehicle along the body-fixed *x*-axis is described by Newton's second law as Equation 39:


$$\begin{array}{l} mẍ=\sum \;F_{x}-F_{aero\;}-F_{roll\;} \end{array}$$ (39)

where *m* is the vehicle mass, ∑ *F*_*x*_ is the net propulsion and braking force, and


$$\begin{array}{l} F_{aero\;}=\frac{1}{2}\rho C_{d}A_{f}{ẋ}^{2} \end{array}$$

is the aerodynamic drag force. The rolling-resistance force is modeled as:


$$\begin{array}{l} F_{roll\;}=mgf_{r} \end{array}$$

where ρ is air density, *C*_*d*_ is the aerodynamic drag coefficient, *A*_*f*_ is the frontal area, *g* is gravitational acceleration, and *f*_*r*_ is the rolling-resistance coefficient.

The roll dynamics about the longitudinal axis are given by Equation 40:


$$\begin{array}{l} I_{xx}\ddot{\phi }=mh_{CG}\left(ÿ+v_{x}\dot{\psi }\right)-K_{\phi }\phi -D_{\phi }\dot{\phi } \end{array}$$ (40)

where *I*_*xx*_ is the roll moment of inertia, *h*_*CG*_ is the center-of-gravity height, *K*_ϕ_ is the total roll stiffness, and *D*_ϕ_ is the roll damping coefficient.

The pitch dynamics about the lateral axis are described by Equation 41:


$$\begin{array}{l} \begin{array}{l} I_{yy}\ddot{\theta }=\;l_{f}\left[K_{f}\left(z-l_{f}\theta \right)+D_{f}\left(ż-l_{f}\dot{\theta }\right)\right]\\ \;-l_{r}\left[K_{r}\left(z+l_{r}\theta \right)+D_{r}\left(ż+l_{r}\dot{\theta }\right)\right]-mh_{CG}ẍ \end{array} \end{array}$$ (41)

where *I*_*yy*_ is the pitch moment of inertia, *l*_*f*_ and *l*_*r*_ are the distances from the center of gravity to the front and rear axles, respectively, and *K*_*f*_, *K*_*r*_, *D*_*f*_, *D*_*r*_ denote the front and rear suspension stiffness and damping coefficients.

The yaw dynamics about the vertical axis are expressed as Equation 42:


$$\begin{array}{l} I_{zz}\ddot{\psi }=2F_{yf}l_{f}cos\delta -2F_{yr}l_{r} \end{array}$$ (42)

where *I*_*zz*_ is the yaw moment of inertia, *F*_*yf*_ and *F*_*yr*_ are the front- and rear-axle lateral tire forces, and δ is the front-wheel steering angle. The factor of two accounts for the two tires at each axle under a symmetric axle force representation.

Under the small-angle approximation, the lateral tracking error is represented by equation 43:


$$\begin{array}{l} e={\left[\begin{array}{cccc} e_{y} & {ė}_{y} & e_{\psi } & {ė}_{\psi } \end{array}\right]}^{T} \end{array}$$ (43)

where *e*_*y*_ is the lateral position error relative to the reference Bézier path and


$$\begin{array}{l} e_{\psi }=\psi -\psi_{des} \end{array}$$

is the heading error. The continuous-time tracking model is written as Equation 44:


$$\begin{array}{l} \dot{e}=Ae+B_{1}\delta +B_{2}{\dot{\psi }}_{des} \end{array}$$ (44)

where *v*_*x*_ is the forward velocity, *C*_α*f*_ and *C*_α*r*_ are the front- and rear-axle cornering stiffness coefficients, and the state matrices are as Equation 45:


$$\begin{array}{r} A=\left[\begin{array}{cccc} 0 & 1 & 0 & 0\\ 0 & -\frac{2C_{\alpha f}+2C_{\alpha r}}{mv_{x}} & \frac{2C_{\alpha f}+2C_{\alpha r}}{m} & \frac{-2C_{\alpha f}l_{f}+2C_{\alpha r}l_{r}}{mv_{x}}\\ 0 & 0 & 0 & 1\\ 0 & \frac{-2C_{\alpha f}l_{f}+2C_{\alpha r}l_{r}}{I_{zz}v_{x}} & \frac{2C_{\alpha f}l_{f}-2C_{\alpha r}l_{r}}{I_{zz}} & -\frac{2C_{\alpha f}l_{f}^{2}+2C_{\alpha r}l_{r}^{2}}{I_{zz}v_{x}} \end{array}\right],\\ B_{1}=\left[\begin{array}{c} 0\\ \frac{2C_{\alpha f}}{m}\\ 0\\ \frac{2C_{\alpha f}l_{f}}{I_{zz}} \end{array}\right],\;B_{2}=\left[\begin{array}{c} 0\\ \frac{-2C_{\alpha f}l_{f}+2C_{\alpha r}l_{r}}{mv_{x}}-v_{x}\\ 0\\ -\frac{2C_{\alpha f}l_{f}^{2}+2C_{\alpha r}l_{r}^{2}}{I_{zz}v_{x}} \end{array}\right] \end{array}$$ (45)

The matrices have been written using a consistent notation in which *C*_α*f*_ and *C*_α*r*_ denote axle cornering stiffnesses. Terms such as *C*_*af*_, *C*_*ar*_, *C*_*xf*_, and *P*_*r*_ are not used because they introduce ambiguity or are inconsistent with the lateral bicycle-model formulation.

The steering command is synthesized using LQR state feedback and feedforward curvature compensation is shown in Equation 46:


$$\begin{array}{l} \delta =-Ke+\delta_{ff} \end{array}$$ (46)

where


$$\begin{array}{l} K=\left[\begin{array}{cccc} k_{1} & k_{2} & k_{3} & k_{4} \end{array}\right] \end{array}$$

is obtained by minimizing the quadratic LQR cost function. The feedforward term compensates for the steering demand associated with the desired path curvature and reduces steady-state tracking error as shown in Equation 47:


$$\begin{array}{l} \delta_{ff}=\kappa_{des}\left[l_{f}+l_{r}-l_{r}k_{3}+\frac{mv_{x}^{2}}{l_{f}+l_{r}}\left(\frac{l_{r}}{2C_{\alpha f}}+\frac{l_{f}k_{3}}{2C_{\alpha r}}-\frac{l_{f}}{2C_{\alpha r}}\right)\right] \end{array}$$ (47)

Here, κ_*des*_ is the curvature of the reference Bézier path. The feedback component suppresses transient lateral and heading errors, whereas the feedforward component anticipates the steering angle required to follow the reference curvature at the current longitudinal velocity.

The longitudinal-actuation layer converts the estimated scene risk and relevant vehicle-relative states into a normalized braking command. This layer is implemented through a fuzzy inference mechanism that provides interpretable braking behavior and avoids abrupt transitions between discrete emergency states.

In Table 5, the principal input variables are the aggregated risk *R*_*agg*_, the risk associated with the origin-lane lead vehicle *R*_*A*_, and the risk associated with the target-lane lead vehicle *R*_*D*_. Their linguistic values are defined as Low (L), High (H), and Very High (VH), while the output is the commanded brake pressure *P*_*brake*_.

**Table 5:** Fuzzy linguistic structure and partitioning for brake-pressure output.

Linguistic term	Notation	Numerical domain	Operational interpretation
Release or negative pressure	*N*	*P*_*brake*_ ≤ 0 bar	No braking command; release the brake or maintain propulsion according to the longitudinal controller
Low brake pressure	*L*	0 < *P*_*brake*_ ≤ *P*_*L*_	Mild deceleration for risk attenuation and speed matching
Medium brake pressure	*M*	$\begin{array}{c} P_{L}<P_{brake\;}\leq \\ P_{M} \end{array}$	Moderate deceleration for closing-gap regulation
High brake pressure	*H*	$\begin{array}{c} P_{M}<P_{brake\;}\leq \\ P_{H} \end{array}$	Strong deceleration for high-risk conditions
Very high brake pressure	*VH*	$\begin{array}{c} P_{H}<R_{brake\;}\leq \\ P_{max\;} \end{array}$	Emergency braking for imminent collision risk or critical takeover conditions

The pressure thresholds *P*_*L*_, *P*_*M*_, *P*_*H*_, and *P*_*max*_ are calibrated according to the actuator limits and the desired longitudinal deceleration range. The operational meanings of the output pressure levels are summarized in Table 5. The term “Any” denotes a don't-care condition. Thus, Rule *R*_6_ assigns a high braking level whenever the target-lane lead-vehicle risk is high, irrespective of the other input states. Rule *R*_8_ prioritizes the aggregated scene risk and produces very high braking pressure when the overall risk reaches the critical region. Rule *R*_9_ addresses the compound condition in which both the origin-lane lead-vehicle risk and the target-lane lead-vehicle risk are very high.

The defuzzified output *P*_*brake*_ is passed to the longitudinal controller, which applies actuator saturation and rate limiting before generating the final brake command. This structure preserves interpretability while allowing smooth braking modulation across the transition from nominal lane-change execution to hazard mitigation.

## 7 Scenario design and simulation environment

### 7.1 High-fidelity SCANeR studio and simulink co-simulation

The driving scenarios in this study are developed using the Rescan simulation platform, which provides an integrated and comprehensive environment for modeling vehicle dynamics, sensor configurations, and traffic interactions. Rescan enables seamless coupling with Simulink through a dedicated compilation interface, allowing for the efficient implementation and evaluation of advanced control and decision-making algorithms. Within this framework, the Rescan graphical user interface is primarily employed for scenario construction and configuration, while dynamic entities and simulation execution are managed through automatically generated model blocks.

All dynamic objects within the simulated environment are represented using the Rescan Actors toolbox, which supports a wide range of traffic participants, including passenger vehicles, trucks, motorcycles, bicycles, and pedestrians. Each actor can be independently configured in terms of vehicle dynamics, driver behavior, physical characteristics, and predefined or observed motion trajectories. Once these actors are incorporated into the graphical environment, dedicated simulation blocks are automatically created, enabling the direct integration of control algorithms within the Simulink platform without the need for manual coding of vehicle interactions.

To enable realistic perception of the driving environment, the Rescan Sensors toolbox is utilized to provide a variety of onboard sensing modules, including radar, LiDAR, ultrasonic sensors, and camera systems. These sensors can be attached to any actor in the scenario and individually configured with specific parameters and customized signal-processing pipelines. Such sensor modeling plays a crucial role in reproducing realistic driving conditions and ensuring accurate perception of surrounding traffic and environmental risks.

The overall objective of the scenario design is to create a highway driving environment in which the subordinate autonomous vehicle (Sub-AV) is required to perform a lane-change maneuver. As discussed previously, one of the key capabilities expected from an SAE Level-3 autonomous vehicle is the ability to independently execute lane-change maneuvers on highways and expressways. Accordingly, the Sub-AV must autonomously identify an appropriate exit location, determine the required number of lane changes, and perform the maneuver safely and efficiently. These considerations form the core foundation of the scenario design and simulation environment employed in this research.

### 7.2 Operational Design Domain (ODD) scenarios

#### 7.2.1 Scenario A: Nominal cut-in and lane-change

Three distinct driving scenarios are designed in this study, each defined by different initial conditions, traffic flow densities, and dynamic interactions among surrounding vehicles. Traffic conditions are modeled as time-varying to realistically reflect changes encountered in highway environments. At the beginning of each scenario, the lane-change system receives a command from the autonomous driving module, initiating both trajectory planning and maneuver execution. Throughout the maneuver, the lane-change unit continuously monitors surrounding traffic conditions, evaluates the associated risk levels, and determines whether the maneuver should be completed as planned, modified, or aborted to ensure safety.

To enhance safety, a warning and risk management strategy is incorporated into the proposed framework. When the evaluated risk level increases during the lane-change maneuver without reaching a critical threshold, an early warning is issued to indicate elevated but manageable risk. A critical warning is triggered if the maneuver cannot be completed within a predefined time window, particularly when the vehicle is approaching a highway exit. In the absence of driver intervention following these warnings, the autonomous system responds by either executing a minimum-risk maneuver under critical conditions or safely decelerating and stopping the vehicle when continuation of the lane-change maneuver is deemed infeasible. It is worth noting that manual takeover or driver return is not considered an objective of this work; instead, system safety is always prioritized over maneuver completion.

The road environment and static infrastructure are modeled using the infrastructure toolbox, which enables the construction of realistic highway scenarios. This toolbox is employed to define straight and curved road segments, highway entrances and exits, intersections and roundabouts, as well as lane markings, boundaries, traffic signs, and surrounding buildings. Such detailed infrastructure modeling ensures that the simulated scenarios closely resemble real-world driving conditions and provides a reliable platform for evaluating the proposed lane-change and risk-management strategies in complex traffic environments.

#### 7.2.2 Scenario B: Abort maneuver due to sudden target lane intrusion

For all lane-change scenarios considered in this study, a straight three-lane highway is constructed, including one exit and a connecting on-ramp to realistically represent freeway driving conditions. The total length of the highway is set to 1,500 m, and a reference coordinate system with its origin located at (0,0) is defined at the beginning of the road centerline.

At the start of each simulation, the subordinate autonomous vehicle (Sub-AV) is initially positioned in the center lane at a longitudinal distance of 300 m from the beginning of the highway. Under this initial configuration, the remaining distance from the vehicle to the highway exit is 700 m. The primary objective of the maneuver is to execute a right-lane change into the adjacent lane, which is defined with a standard lane width of 3.75 m.

#### 7.2.3 Scenario C: Multi-agent high-density highway congestion

In each driving scenario in Figure 11, the subordinate autonomous vehicle (Sub-AV) operates in the presence of surrounding traffic vehicles, whose longitudinal positions, velocities, and accelerations vary dynamically throughout the simulation to reflect realistic highway conditions. The Audi A8 sedan is selected as the reference autonomous vehicle platform due to its well-documented dynamic behavior and suitability for automated driving research. The physical, inertial, and actuation parameters are detailed in Table 6. These parameters are adopted consistently across all scenarios to provide a reliable and reproducible foundation for evaluating the proposed lane-change and decision-making strategies.

**Figure 11:**
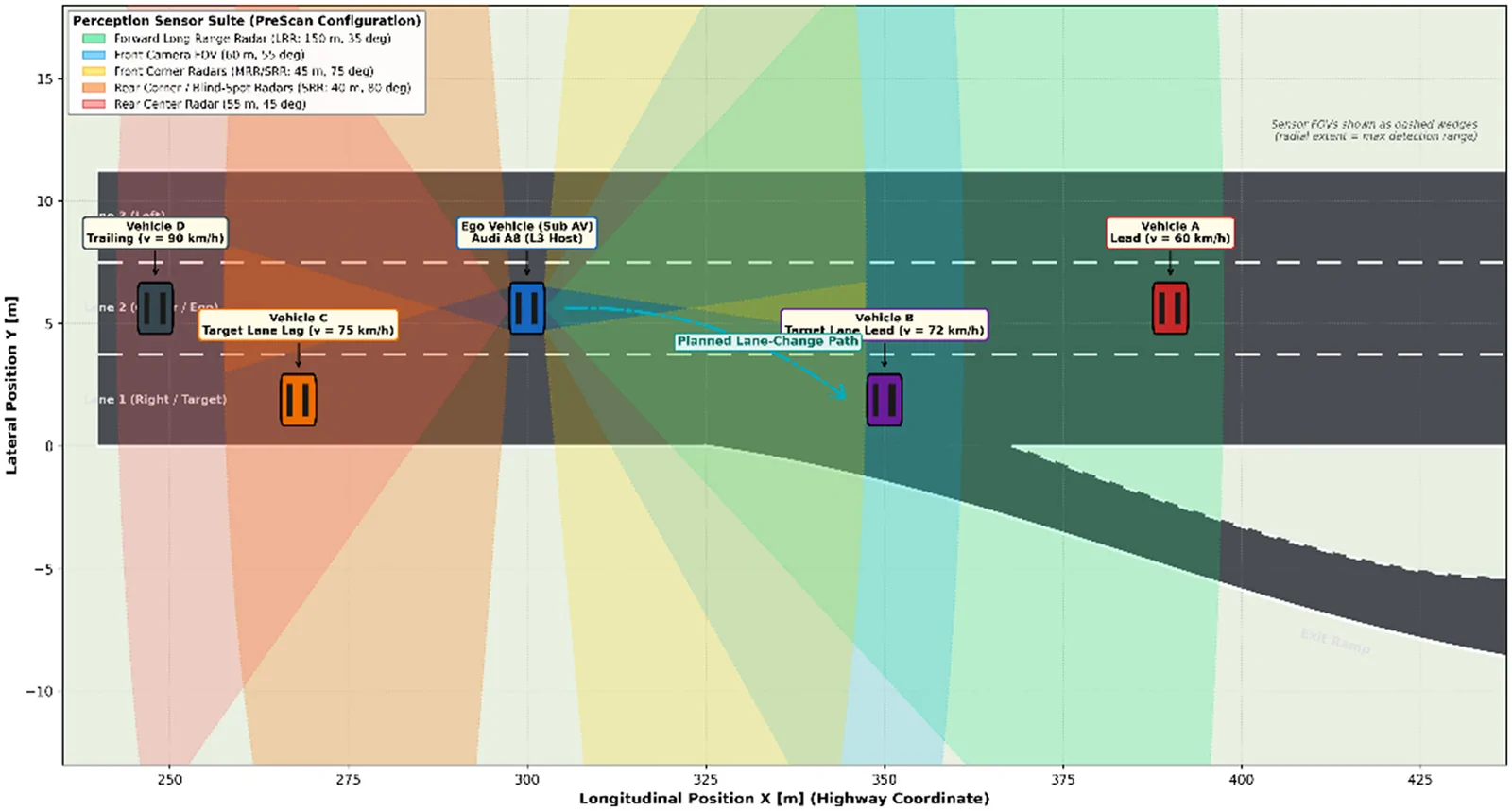
Top-view visualization of sensor placement and perception coverage in the Rescan simulation environment.

**Table 6:** Physical, inertial, and actuation parameters of the Audi A8 platform.

System parameter	Symbol	Value	Units	Engineering description
Total Vehicle Mass	*m*	1,820	kg	Curb mass including instrumentation
Yaw Moment of Inertia	*I*_*zz*_	3,746	*kg*·*m*^2^	Rotational inertia around vertical axis
CG to Front Axle Distance	*l*_*f*_	1.17	m	Longitudinal lever arm to front axle
CG to Rear Axle Distance	*l*_*r*_	1.77	m	Longitudinal lever arm to rear axle
Front Tire Cornering Stiffness	*C*_α*f*_	72,653	N/rad	Linearized cornering compliance (per axle)
Rear Tire Cornering Stiffness	*C*_α*r*_	121,449	N/rad	Linearized cornering compliance (per axle)
Overall Steering Gear Ratio	*i*_*s*_	20.0:1	-	Handwheel to road wheel kinematic ratio
Maximum Handwheel Angle	δ_*sw, max*_	±500	deg	Physical travel limit of steering column
Max Allowable Acceleration	*a*_*x, max*_	+2.94	*m*/*s*^2^	Longitudinal powertrain throttle cap (+0.30 g)
Max Allowable Deceleration	*a*_*x, min*_	−9.81	*m*/*s*^2^	Maximum emergency brake deceleration (−1.00 g)

After completing the scenario construction, the autonomous vehicle is equipped with a perception sensor suite suitable for SAE Level 3 automated driving. The Rescan environment distinguishes between ideal and realistic sensors. Ideal sensors assume perfect detection performance, whereas realistic sensors account for measurement noise, occlusions, and misclassification. In this study, only the Sub-AV is equipped with realistic sensors, while surrounding vehicles follow predefined trajectories.

The configuration includes six radar units (two long-range radars on the front/rear bumpers and four corner radars) and a forward-looking camera. These are characterized by detection range, field of view (FOV), maximum detectable objects, resolution, and antenna configuration. Figure 11 illustrates the sensor placement and sensing boundaries.

This diagram demonstrates the sensor topology, illustrating the effective field-of-view (FOV) of the radar array and front-facing camera on the Sub-AV. The spatial distribution ensures comprehensive environmental coverage, facilitating the detection of adjacent and leading vehicles necessary for high-fidelity decision-making.

In this study, a right-adjacent lane change maneuver is investigated based on the scenario depicted in Figure 11. Vehicles A, B, and D are considered the primary surrounding vehicles in the simulated lane-change scenarios. Their initial kinematic boundary conditions are summarized in Table 7.

**Table 7:** Initial kinematic boundary conditions of traffic participants across scenarios.

Vehicle identifier	Role in maneuver	Initial station *X*_0_ (m)	Initial velocity *v*_*x*, 0_(*m*/*s*)	Initial lane
Sub-AV (Ego)	Subject Autonomous Vehicle	300.0	23.00	Center
Vehicle A	Target Lane Lead	400.0	16.67	Right
Vehicle B	Target Lane Lag	350.0	20.00	Right
Vehicle D	Origin Lane Lag	285.0	25.00	Center

Figure 12 shows a schematic representation of the right-lane change maneuver performed by the ego vehicle. Vehicles A, B, and D denote traffic participants whose relative positions and velocities dynamically influence the ego vehicle's lane-change decision and safety constraints.

**Figure 12:**
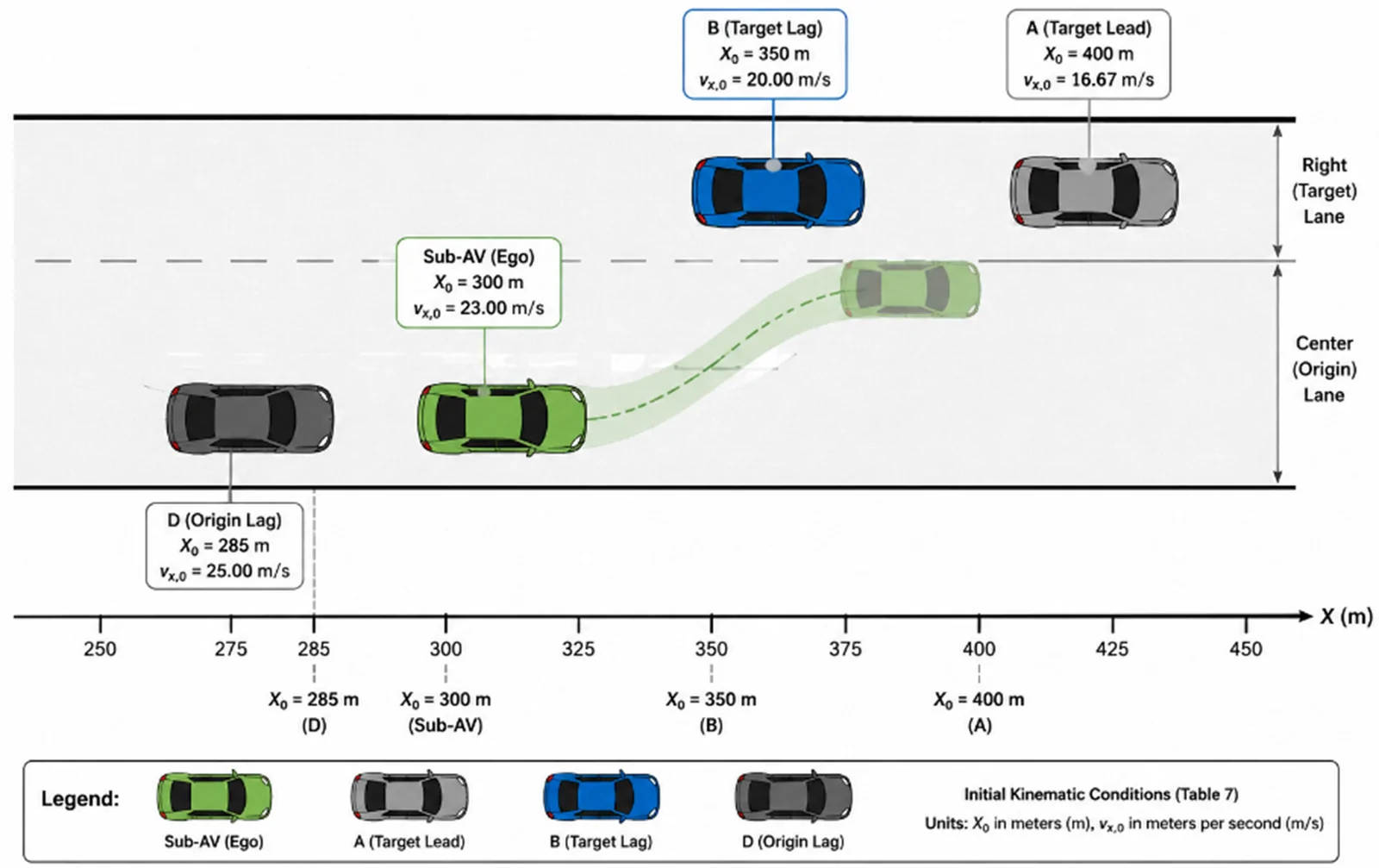
Schematic of the right-lane change scenario involving surrounding vehicles.

## 8 Simulation results and discussion

### 8.1 Scenario A: Baseline lane change

Figure 13 illustrates five discrete snapshots of the first driving scenario, capturing the temporal evolution of the ego vehicle during longitudinal motion and interaction. The left column displays bird's-eye views, while the right column shows rear-perspective views at *t* = 0, 6.6, 13.15, 29.1, and 45.45 s, respectively, highlighting the maneuver execution phase.

**Figure 13:**
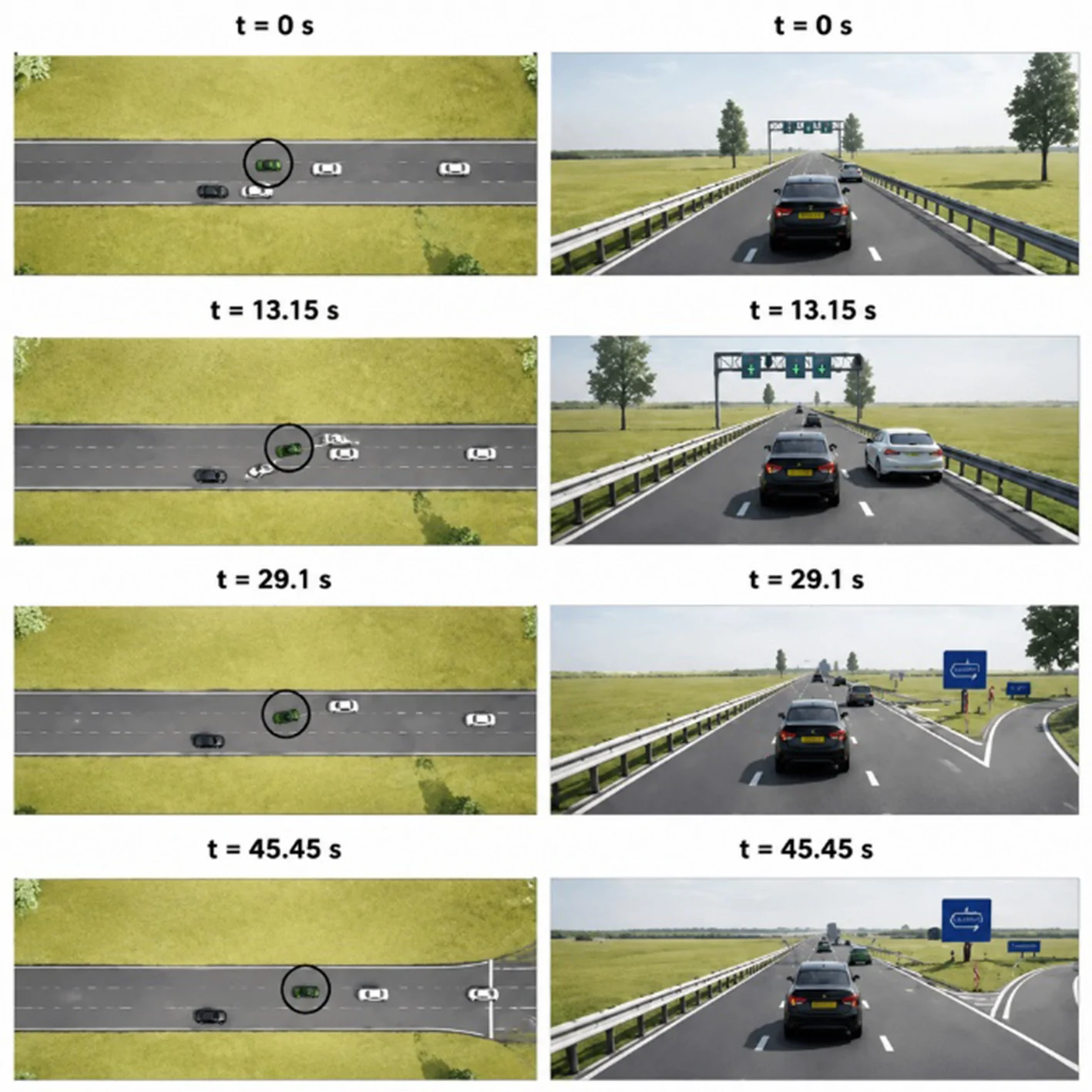
Time-sequential visualization of autonomous vehicle maneuvering and lane-level interaction in a multi-lane highway scenario.

Figure 14 displays the lateral displacement (*Y*) vs. longitudinal distance (*X*) for the Sub-AV in Scenario 1. The trajectory remains constant along the centerline (*Y* = 0), confirming that the high-level decision unit successfully executed a rejection command due to safety-critical risk thresholds.

**Figure 14:**
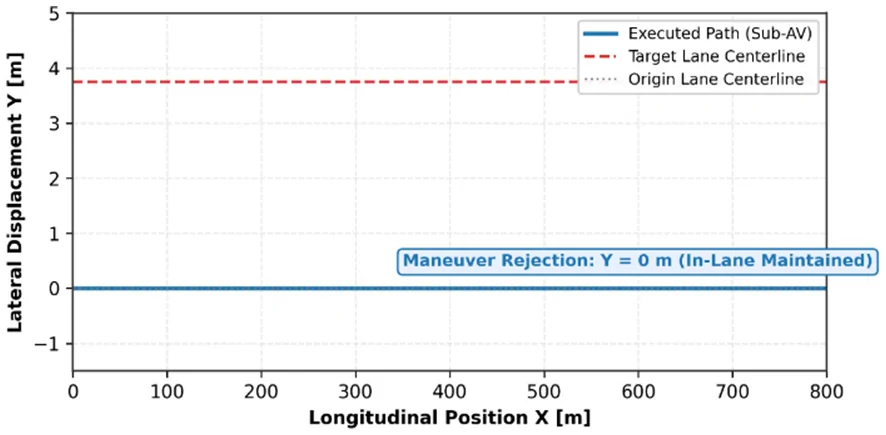
Lateral trajectory analysis of the Sub-AV demonstrating maneuver rejection in Scenario 1.

Figure 15 depicts the temporal evolution of risk metrics A, B, and D. The data show that Risk A and D frequently exceed the critical threshold (≈0.93), providing the empirical justification for the maneuver rejection observed.

**Figure 15:**
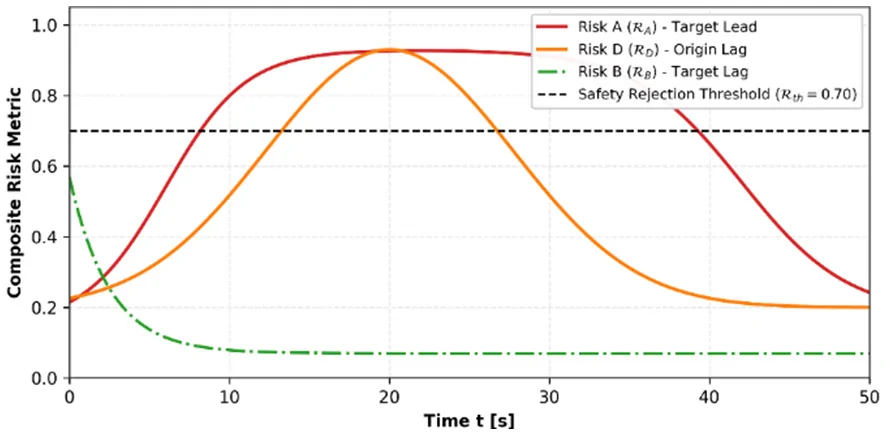
Temporal analysis of composite risk metrics (A, B, and D) demonstrating safety threshold exceedance in Scenario 1.

Figure 16 shows acceleration histories. The lateral acceleration remains near zero throughout the scenario, confirming the absence of lateral maneuvers, while longitudinal profiles reflect initial stabilization adjustments consistent with high-risk environment mitigation.

**Figure 16:**
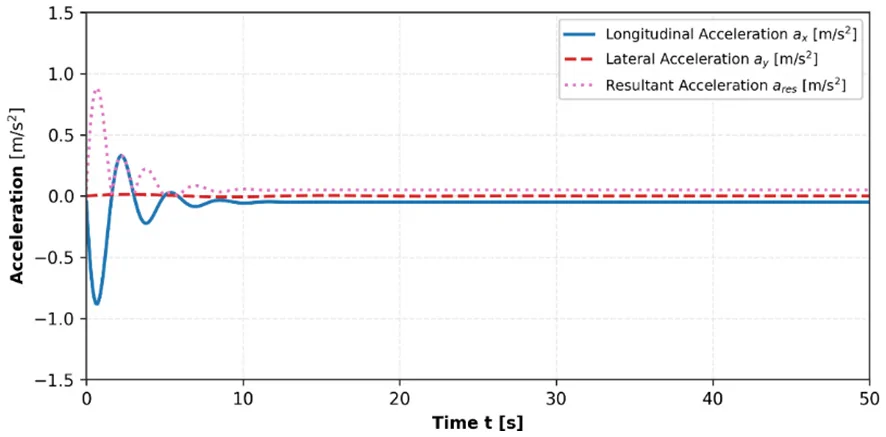
Longitudinal, lateral, and overall acceleration profiles of the Sub-AV in Scenario 1.

Figure 17 tracks brake pressure (bar) over time. The sharp initial pressure peaks correlate with collision mitigation responses, followed by a transition to a stable following mode with minimal oscillations, ensuring safe distance maintenance.

**Figure 17:**
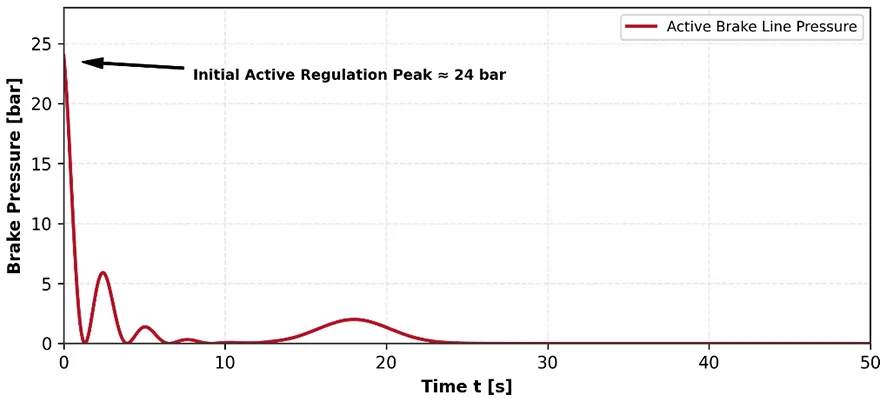
Actuation dynamics of brake pressure for the Sub-AV in high-risk Scenario 1.

Figure 18 illustrates the TTM evolution and HMI warning stages (Staged vs. Imminent). The decline to zero at *t*≈40 s marks the critical decision deadline, beyond which safe autonomous lane-change execution is not guaranteed.

**Figure 18:**
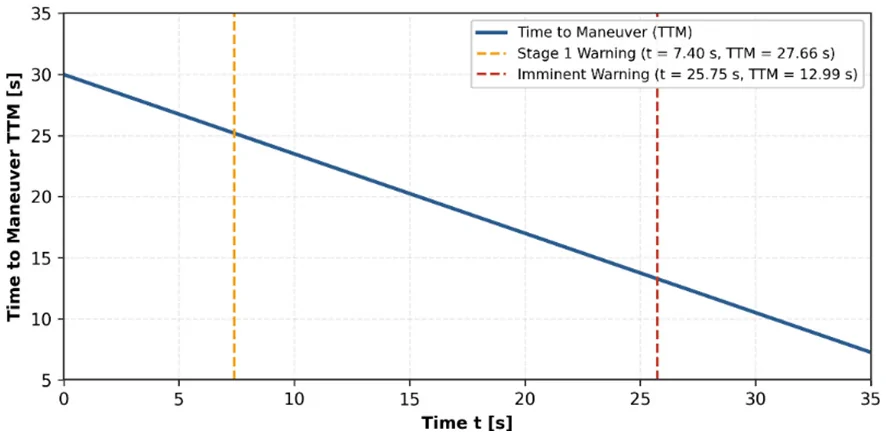
Time to maneuver (TTM) trajectory and HMI warning stages in high-risk Scenario 1.

### 8.2 Scenario B: Dynamic abort maneuver execution

Figure 19 illustrates the spatial and temporal evolution of the ego vehicle's lane-change decision process under varying traffic interactions in Scenario 3, using both top-down and ego-centric perspectives. At the initial stage (*t* = 0 s), the ego vehicle maintains steady longitudinal motion with adequate inter-vehicle spacing, indicating a low-risk driving condition. As time progresses (*t* = 5 s), surrounding vehicle configurations gradually change, altering the relative distance and velocity profiles within the target lane. At *t* = 8.05 s, increased interaction with nearby vehicles—particularly the approaching traffic from the adjacent lane—leads to a noticeable rise in calculated risk, reflected in the constrained maneuver feasibility. By *t* = 11 s, the dynamic traffic environment reaches a critical state where lateral maneuver execution becomes safety-sensitive due to reduced gap availability and relative speed mismatch. The combined views emphasize how real-time risk assessment and surrounding vehicle behavior jointly influence the decision-making mechanism, highlighting the system's ability to continuously monitor spatial constraints, adapt maneuver timing, and ensure collision-free operation under complex traffic conditions.

**Figure 19:**
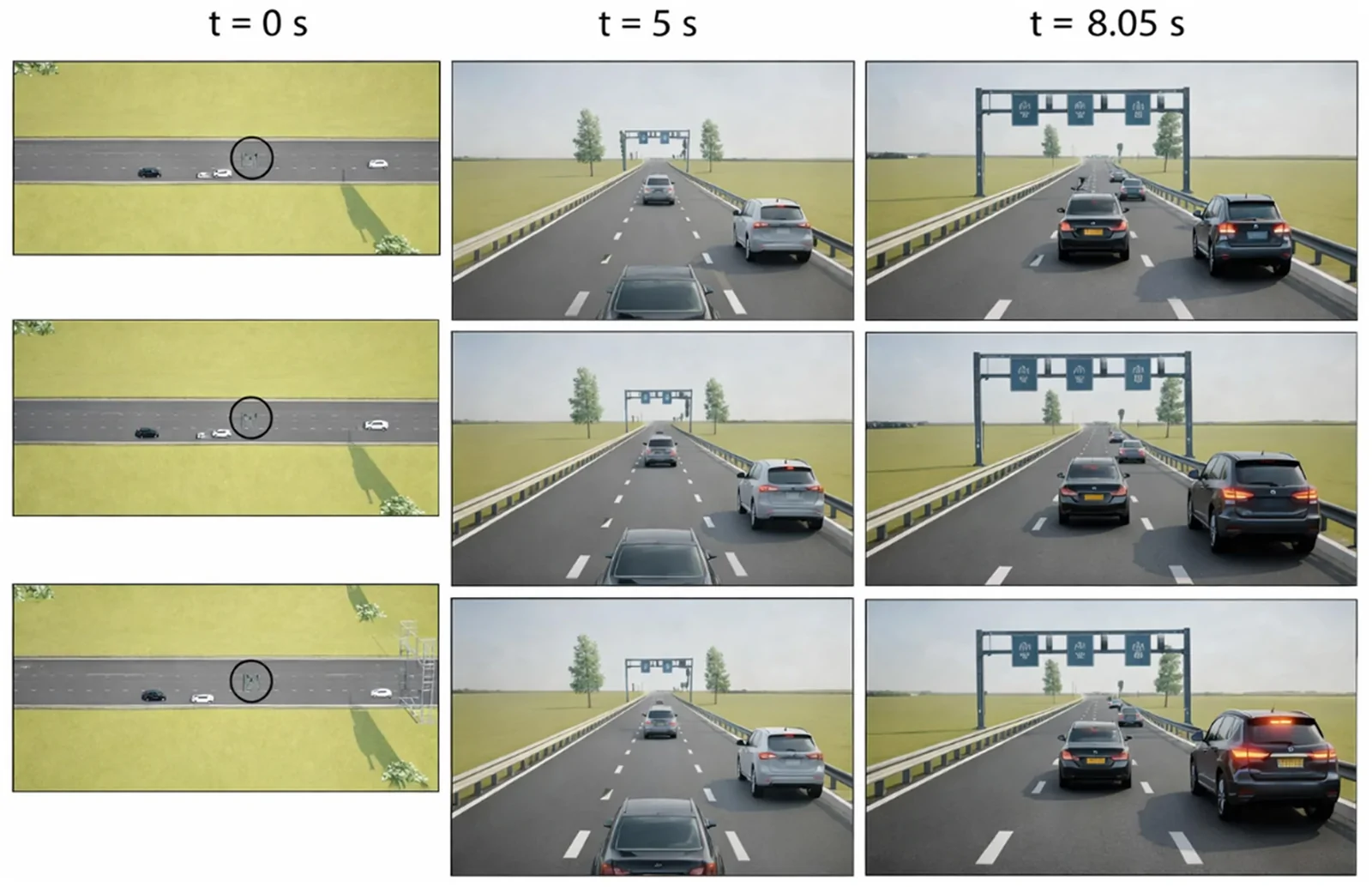
Spatial-temporal evolution of lane-change risk assessment and traffic interaction in Scenario 3.

Figure 20 illustrates the comparison between the initially planned lane-change trajectory and the actual path executed by the Sub-Autonomous Vehicle (Sub-AV) following an aborted maneuver. The dotted red curve represents the reference path generated by the motion planner under nominal conditions, while the solid blue curve depicts the trajectory traveled after the lane-change decision was canceled. As the Sub-AV detects unfavorable environmental or traffic conditions, the decision module initiates a maneuver abort, resulting in a smooth trajectory reversion toward the original lane. This divergence highlights the system's ability to adaptively modify its trajectory in real time while maintaining motion continuity and lateral stability. Notably, the absence of sharp curvature discontinuities demonstrates the effectiveness of the underlying path planner in preserving passenger comfort and vehicle safety during maneuver cancellation. The results confirm that the proposed planning and decision framework can robustly handle dynamic traffic uncertainties by safely transitioning from a planned lane change to a fallback trajectory without inducing abrupt vehicle responses.

**Figure 20:**
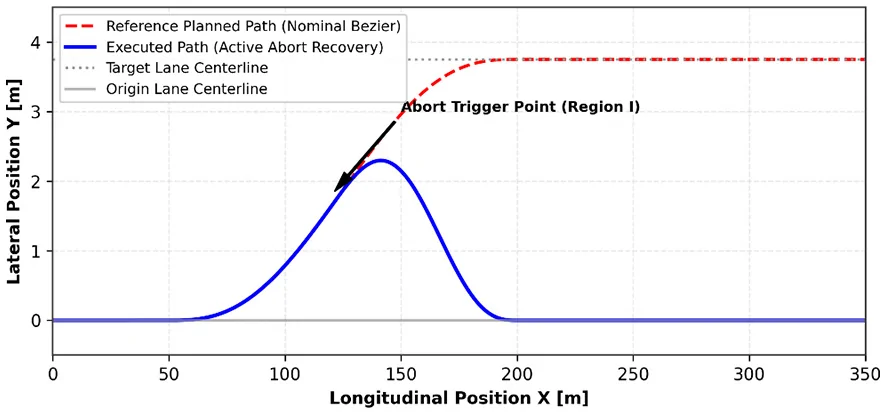
Trajectory deviation analysis during aborted lane-change of the Sub-AV.

Figure 21 illustrates the comparison between the initially planned lane-change path and the actual trajectory executed by the Sub-AV after aborting the maneuver, together with the associated longitudinal, lateral, and resultant acceleration responses. The planned path exhibits a smooth lateral deviation consistent with a deliberate lane-change strategy, whereas the executed trajectory deviates significantly and returns to the original lane, indicating a real-time decision to abort the maneuver due to dynamic traffic constraints. This decision is clearly reflected in the acceleration profiles, where a pronounced negative longitudinal acceleration is observed around the maneuver interruption, signifying active braking to maintain safety margins. Simultaneously, the lateral acceleration shows a transient peak caused by the partial lateral displacement before corrective steering is applied. The overall acceleration curve captures the combined effect of these responses, demonstrating a well-coordinated trade-off between comfort and safety. Importantly, the bounded acceleration magnitudes confirm that the motion remains within acceptable vehicle dynamic limits, highlighting the capability of the Sub-AV control system to promptly adapt its trajectory and motion states in response to evolving environmental conditions while ensuring stability and passenger comfort.

**Figure 21:**
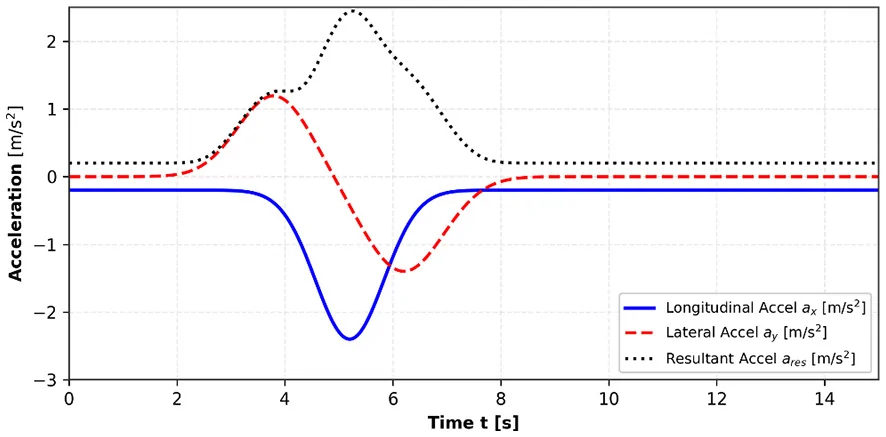
Planned and executed trajectories of the Sub-AV during an aborted lane-change maneuver in Scenario 2, along with the corresponding acceleration profiles.

Figure 22 presents the time histories of the steering wheel angle and the corresponding yaw rate of the Sub-AV during the aborted lane-change maneuver in Scenario 2. Initially, both signals remain close to zero, indicating stable straight-line driving. As the lane-change is initiated, a sharp increase in steering input is observed, generating a pronounced yaw rate response that reflects the vehicle's rotational motion required for lateral displacement. However, shortly after the maneuver onset, a rapid steering reversal occurs, accompanied by a significant negative yaw rate, which clearly signifies the abort decision and the corrective action to realign the vehicle with the original lane. The subsequent oscillations with diminishing amplitude indicate closed-loop stabilization by the lateral control system. Importantly, the strong temporal correlation between the steering command and yaw rate demonstrates effective yaw responsiveness, while the bounded yaw rate magnitude confirms that vehicle stability limits are not violated. These results highlight the capability of the Sub-AV control architecture to promptly modulate steering and yaw dynamics in response to real-time risk assessment, ensuring safe recovery from an aborted lane-change maneuver.

**Figure 22:**
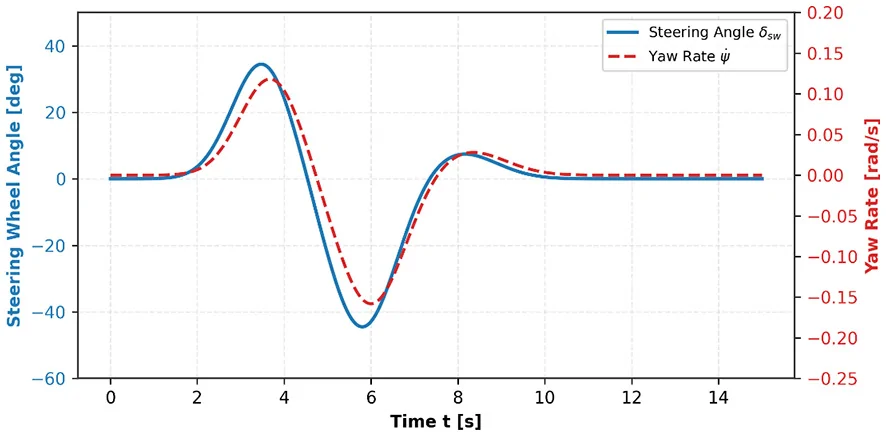
Temporal evolution of steering input and yaw rate response of the Sub-AV during an aborted lane-change maneuver in Scenario 2.

Figure 23 depicts the time evolution of risk indices associated with the surrounding vehicles during Scenario 2, highlighting the dynamic risk assessment process employed by the Sub-AV. At the initial stage, the risk values remain relatively stable, indicating a low-threat traffic configuration. As the lane-change maneuver is initiated, a noticeable increase in the risk metric—particularly for the conflicting vehicle—can be observed, reflecting reduced safety margins caused by decreasing longitudinal and lateral gaps. The dashed vertical markers corresponding to different maneuver progress parameters (τ) emphasize the transition from an acceptable to a critical risk region. Notably, as the risk exceeds a predefined threshold, a rapid divergence in the risk profiles emerges, triggering the abort decision. Following the maneuver cancellation, the dominant risk values gradually decrease, demonstrating the effectiveness of the corrective longitudinal and lateral control actions in restoring safe inter-vehicle spacing. This behavior confirms that the risk assessment module provides timely and consistent inputs to the decision-making layer, enabling proactive maneuver termination and reinforcing the overall safety and robustness of the autonomous driving framework.

**Figure 23:**
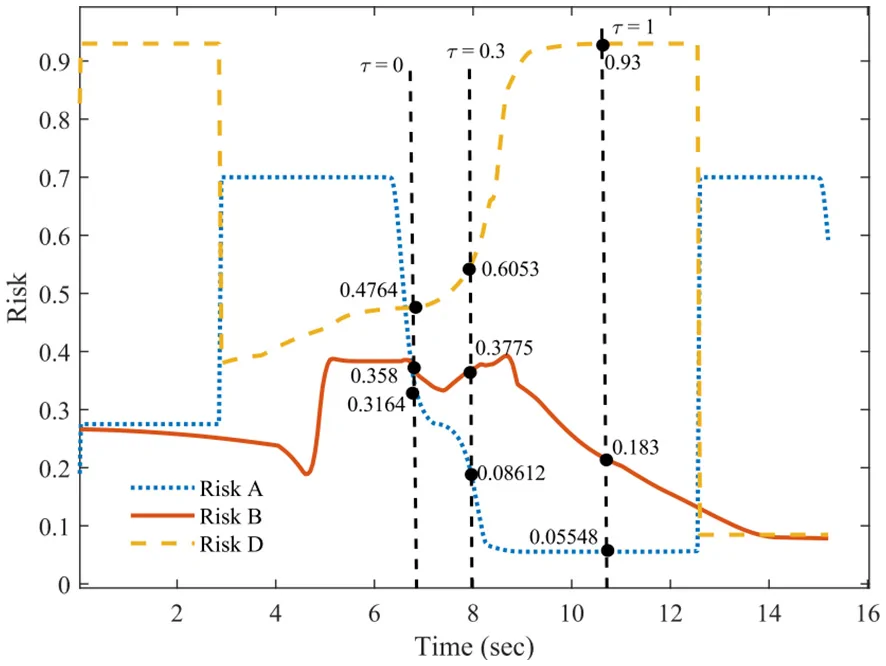
Temporal variation of risk levels for surrounding vehicles during the aborted lane-change maneuver in Scenario 2.

In this scenario, the ego vehicle initially follows another vehicle (vehicle D) in the target lane while the leading vehicle moves ahead of it. As a result, the risk level associated with the target lane decreases, and the lane-change maneuver is initiated. Unlike the previous scenario, the increase in risk after the maneuver initiation does not lead to the cancellation of the lane change. Instead, the vehicle continues the maneuver, passes the midpoint of the lane transition, and successfully completes it.

### 8.3 . Scenario C: Dense multi-agent highway traffic

Figure 24 presents a time-sequential visualization of the ego vehicle's decision-making process during a complex lane-change maneuver under evolving traffic risk conditions (Scenario 3). At the initial stage (*t* = 0 s), the ego vehicle follows the leading traffic with sufficient headway, indicating a low-risk state. As surrounding vehicles change their relative positions (*t* = 5.7 s and *t* = 11.6 s), the ego vehicle evaluates the dynamic interaction with both the lead and adjacent vehicles, while maintaining longitudinal stability. At *t* = 13.7 s, the maneuver progresses toward the mid-phase of lane transition, where the proximity and deceleration behavior of the neighboring vehicle significantly increase the instantaneous collision risk. Unlike conservative strategies that would terminate the maneuver prematurely, the proposed control and decision framework allows continued execution while actively monitoring safety constraints. Finally, at *t* = 15.3 s, a safety warning is triggered, clearly indicated by the hazard symbol, reflecting the system's real-time risk reassessment and intervention capability. This sequence demonstrates the controller's ability to balance maneuver completion and safety assurance, highlighting robust situational awareness, smooth longitudinal-lateral coordination, and timely safety intervention under critical traffic conditions.

**Figure 24:**
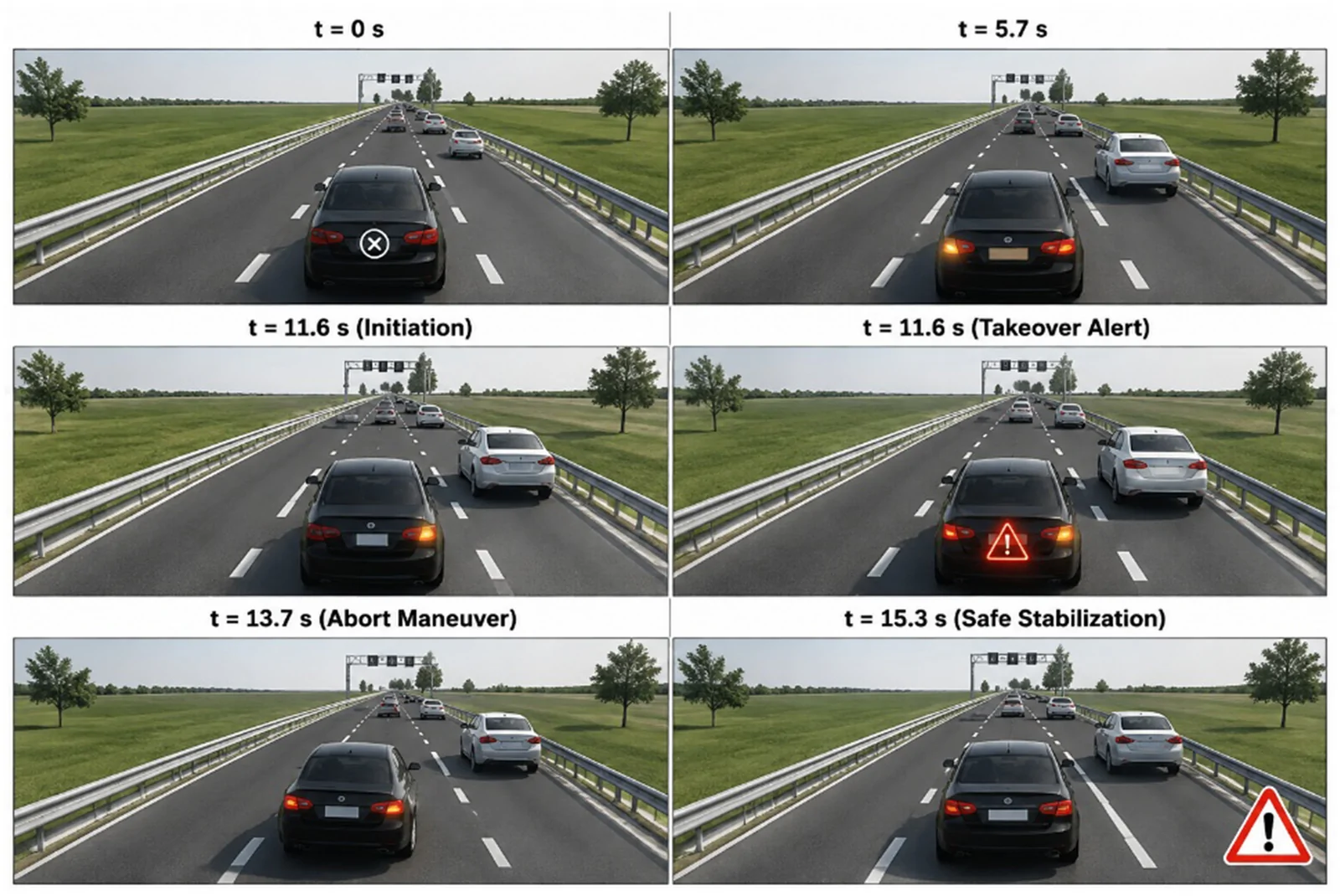
Time-sequential visualization of risk-aware lane-change decision and safety intervention in Scenario 3.

Figure 25 presents a detailed comparison between the initially generated lane-change trajectory and the actual path executed by the Sub-Autonomous Vehicle (Sub-AV) during a lane-change maneuver. The dashed red curve represents the pre-planned reference path, generated using a smooth trajectory planning strategy, while the solid blue curve illustrates the real traveled path of the vehicle. As shown, the vehicle initially follows the reference trajectory with high fidelity; however, a noticeable deviation occurs after the lane-change initiation point. This divergence indicates an adaptive response by the Sub-AV to dynamic environmental or risk-related constraints, such as increased collision probability or changing traffic conditions. The discrepancy between the planned and executed paths highlights the vehicle's real-time decision-making capability, where longitudinal deceleration and lateral trajectory modification are employed to safely abort or reshape the maneuver. Importantly, the smooth curvature of the actual path demonstrates that stability and passenger comfort are preserved despite the deviation, avoiding abrupt steering inputs. This behavior confirms the robustness of the motion planning and control framework, as the system prioritizes safety while maintaining kinematic continuity. Overall, the Figure 25 substantiates the effectiveness of the proposed path-planning and risk-aware control strategy in enabling adaptive and safe lane-change execution under evolving driving conditions.

**Figure 25:**
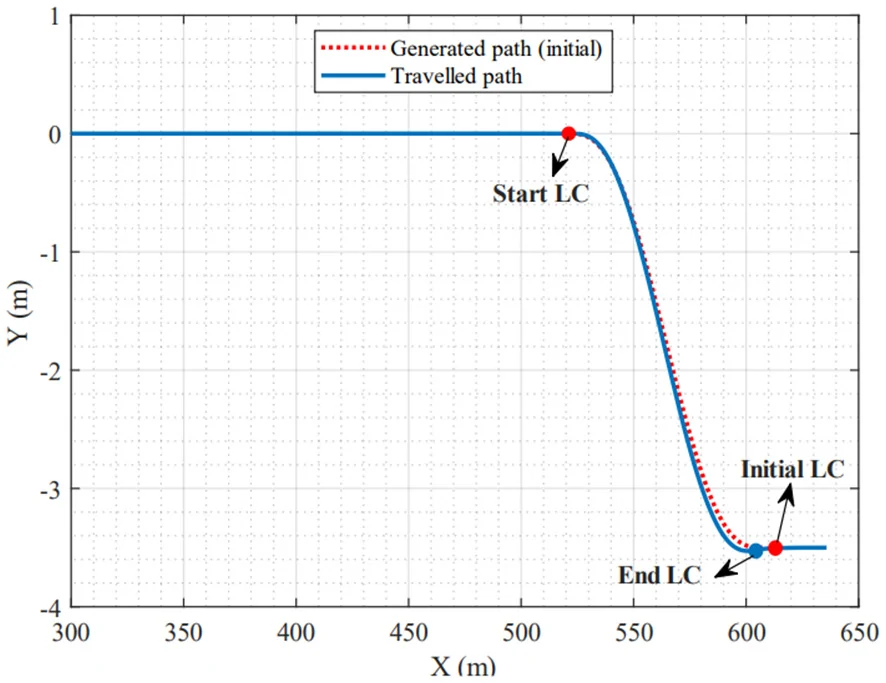
Comparative analysis of planned and executed lane-change trajectories in Sub-AV maneuvering.

Figure 26 illustrates the time-domain profiles of longitudinal acceleration, lateral acceleration, and the resultant (overall) acceleration experienced by the Sub-Autonomous Vehicle (Sub-AV) throughout the maneuver. During the initial phase, pronounced fluctuations in longitudinal acceleration indicate transient deceleration and traction adjustments, which are typically associated with speed regulation and preparatory control actions prior to the maneuver onset. Concurrently, lateral acceleration remains negligible, confirming that no lateral motion or lane-change initiation occurs during this phase. As the maneuver progresses, a significant rise in lateral acceleration is observed, coinciding with the initiation of lateral displacement. This increase reflects the steering-induced centripetal forces required to achieve lateral motion, while the longitudinal acceleration remains moderately negative, indicating a deliberate speed reduction to maintain stability and safety margins. The resultant acceleration, representing the vector combination of longitudinal and lateral components, exhibits clear peaks during the most dynamically intensive segments of the maneuver, particularly near the lane-change execution and termination phases. Notably, the absence of abrupt spikes in the overall acceleration curve demonstrates that the motion planning and control strategy successfully enforces comfort and dynamic feasibility constraints. The smooth acceleration transitions further suggest effective coupling between longitudinal and lateral controllers, ensuring ride comfort, vehicle stability, and compliance with physical limits. Collectively, Figure 26 confirms that the Sub-AV's control framework achieves a well-balanced acceleration profile, enabling responsive yet smooth maneuver execution under dynamic driving conditions.

**Figure 26:**
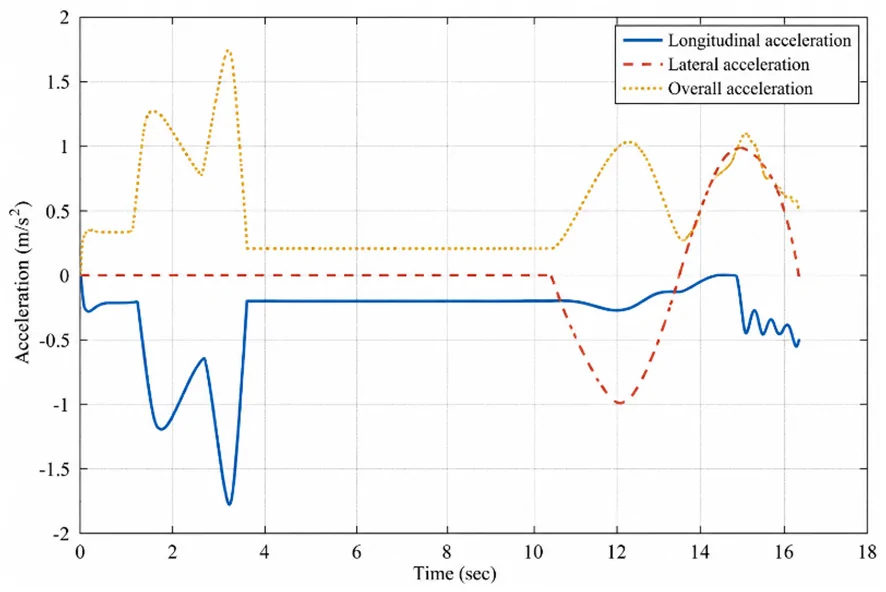
Temporal evolution of longitudinal, lateral, and resultant accelerations during Sub-AV maneuver execution.

Figure 27 illustrates the temporal relationship between the steering wheel input and the resulting yaw-rate response of the Sub-Autonomous Vehicle (Sub-AV) during the lane-change maneuver. The steering angle remains nearly constant in the initial phase, indicating straight-line motion with negligible lateral dynamics. At approximately the maneuver initiation time, a sharp negative steering input is applied, followed by an aggressive positive steering command, reflecting the counter-steering strategy required to initiate and then stabilize the lateral displacement. This steering pattern produces a corresponding yaw-rate response that closely follows the steering input, demonstrating a strong and stable coupling between the driverless control input and vehicle yaw dynamics. Peak yaw-rate values coincide with the maximum steering angles, indicating effective generation of lateral rotational motion necessary for lane transition. Notably, the slight phase lag and the smooth attenuation observed toward the end of the maneuver suggest that the yaw stabilization controller effectively suppresses oscillations and prevents oversteer or understeer conditions. The rapid decay of both steering angle and yaw rate to near-zero values confirms that the vehicle successfully re-enters a stable straight-driving state after maneuver completion. Overall, the Figure 27 validates the responsiveness and robustness of the lateral control architecture, demonstrating its capability to precisely regulate yaw dynamics while ensuring vehicle stability and passenger comfort during aggressive yet safe lane-change maneuvers.

**Figure 27:**
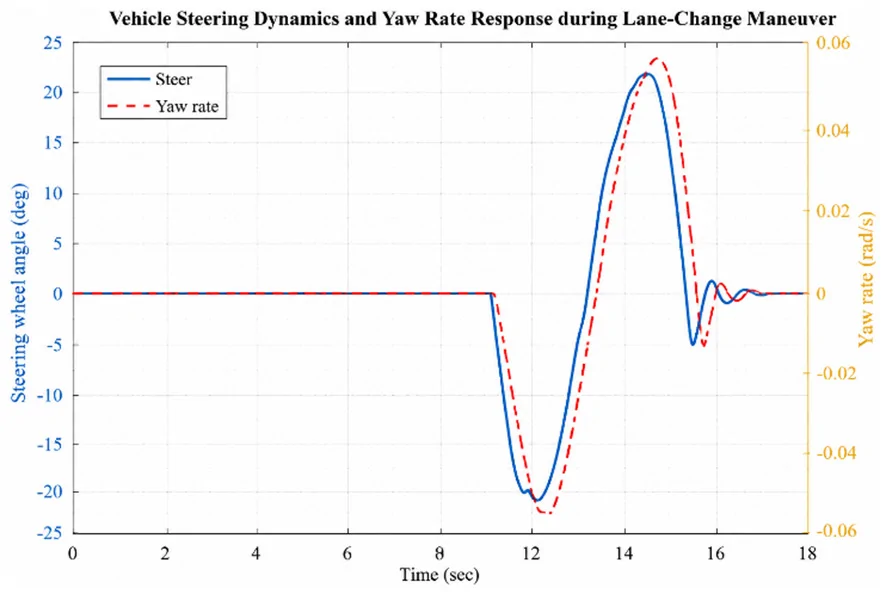
Steering input and corresponding yaw-rate response of the Sub-AV during lane-change execution.

Figure 28 presents the temporal evolution of three distinct traffic risk indicators—Risk A, Risk B, and Risk D—computed for the Sub-Autonomous Vehicle (Sub-AV) during Scenario 3. In the initial phase of the simulation, Risk D dominates with a consistently high magnitude, indicating a critical baseline threat level associated with the surrounding traffic configuration. During this period, both Risk A and Risk B remain at moderate levels, suggesting limited immediate conflict despite the elevated global risk condition.

**Figure 28:**
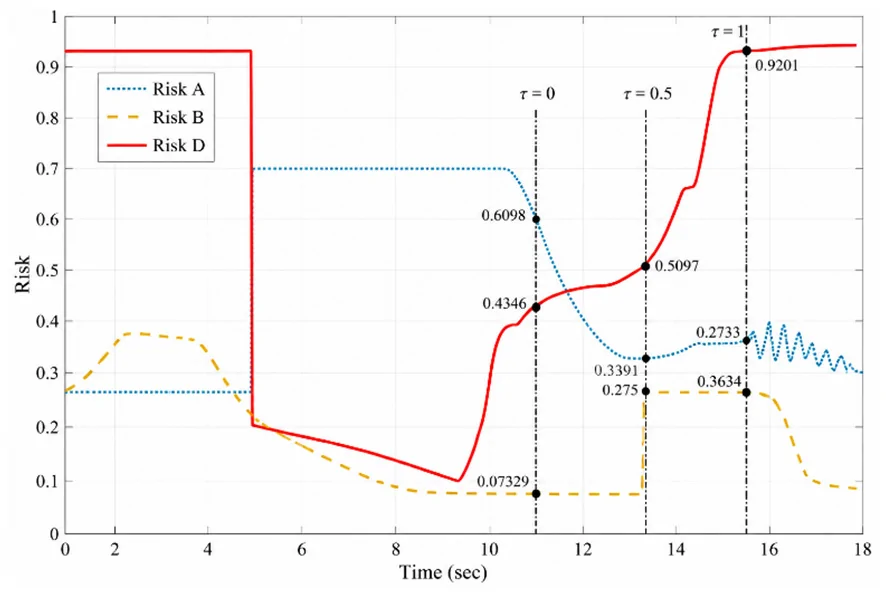
Time-varying evolution of traffic risk metrics for the Sub-AV under Scenario-3 conditions.

As the scenario progresses toward the maneuver initiation interval, a pronounced drop in Risk D is observed, coinciding with a redistribution of risk toward Risk A. This transition reflects a shift from global environmental hazard to localized interaction-based risk, likely caused by changes in relative distances, velocities, or predicted trajectories of neighboring vehicles. At the first annotated time marker, Risk A reaches approximately 0.61, while Risk D is reduced to around 0.43, highlighting an intermediate risk redistribution stage.

Subsequently, near the maneuver execution and corrective control phase, Risk B exhibits a sharp rise from near-minimal values to approximately 0.28–0.36. This behavior indicates an increased probability of lateral or proximity-related conflicts, consistent with lane-change engagement or aborted maneuver dynamics. During this interval, Risk A shows moderate fluctuations, whereas Risk D begins a rapid escalation, reflecting the resurgence of overall collision risk as the traffic density and relative kinematic uncertainty increase.

Notably, at the final critical marker, Risk D reaches its peak value of approximately 0.92, signifying a highly hazardous traffic state that demands immediate corrective or evasive actions. In contrast, Risk B begins to decay after its peak, suggesting that localized conflicts are being mitigated, while the dominant threat transitions back to a global risk perspective. The smooth yet decisive transitions among the three risk curves demonstrate that the proposed risk assessment framework effectively captures both gradual risk accumulation and abrupt hazard escalation. Overall, Figure 28 validates the sensitivity and responsiveness of the multi-dimensional risk evaluation strategy, highlighting its capability to support timely decision-making for autonomous driving in complex and rapidly evolving traffic environments.

Figure 29 illustrates the coupled temporal evolution of the risk metric associated with Target Vehicle A and the corresponding Time-to-Collision (TTC) during Scenario 3. Throughout the initial time interval, the TTC remains sufficiently large, indicating a low probability of imminent collision. During this phase, the risk value stays below moderate thresholds, and no warning related to Vehicle A is triggered, confirming that the interaction does not pose an immediate safety threat. This behavior demonstrates effective situational awareness and conservative spacing maintained by the Sub-Autonomous Vehicle (Sub-AV).

**Figure 29:**
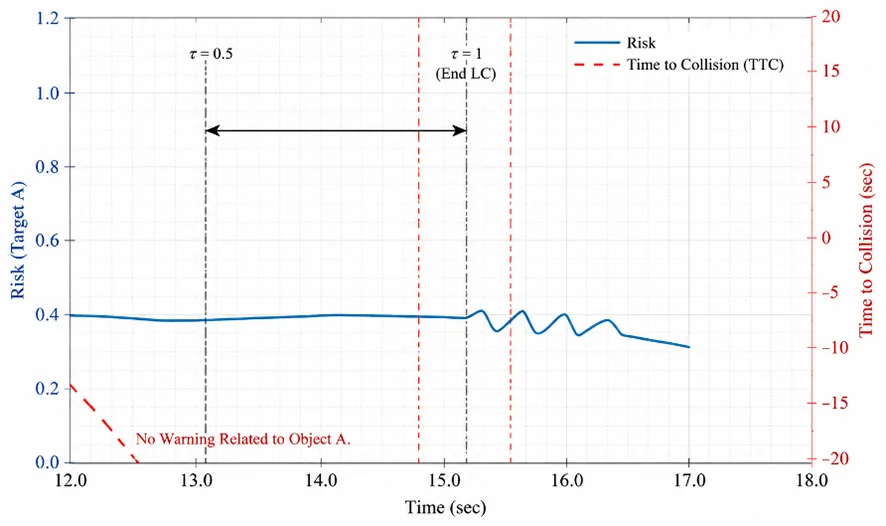
Temporal correlation between risk level and Time-to-Collision (TTC) with Target Vehicle A during Scenario-3.

As the scenario approaches the lane-change termination region, marked by the vertical dashed lines denoting the end of the lane-change (End LC), transient variations in TTC are observed. These TTC fluctuations reflect rapid changes in relative longitudinal distance and velocity caused by lane realignment and minor speed adaptations of the Sub-AV. Despite these changes, the risk magnitude exhibits only small oscillations and does not escalate abruptly, indicating that the predictive risk assessment framework successfully anticipates and mitigates short-term kinematic uncertainty.

Notably, even during periods where TTC decreases toward critical bounds, the risk associated with Target A remains comparatively low, emphasizing that TTC alone does not dominate the risk evaluation. Instead, the proposed framework integrates predictive motion consistency, relative trajectory stability, and temporal evolution to prevent false alarms. The absence of warning activation throughout the maneuver confirms that the Sub-AV maintains sufficient safety margins and collision avoidance capability without unnecessary intervention.

Overall, Figure 29 validates the robustness of the TTC-aware risk assessment module, demonstrating its ability to distinguish between transient kinematic disturbances and genuinely hazardous interactions. The coordinated interpretation of risk and TTC ensures reliable decision-making, reducing false positives while preserving safety in dynamically evolving traffic scenarios.

Figure 30 examines the coupled evolution of the risk index and the Time-to-Collision (TTC) associated with Target Vehicle B during the initiation phase of the lane-change maneuver. At the onset of the maneuver (τ = 0), TTC exhibits large negative values, indicating that no imminent collision is predicted within the system's forecasting horizon. During this interval, the risk level remains near zero, confirming that the Sub-Autonomous Vehicle (Sub-AV) preserves a safe longitudinal and lateral separation from the target vehicle. As the maneuver progresses, TTC increases steadily, reflecting a continuously improving kinematic relationship between the Sub-AV and Target B, likely due to relative speed differences and lateral displacement away from potential conflict zones. Despite this monotonic TTC growth, the risk metric shows only a stepwise and bounded increase, remaining well-below critical thresholds. This behavior highlights an important characteristic of the proposed risk assessment framework: risk escalation is not driven solely by instantaneous TTC values, but by their consistency, temporal evolution, and interaction with maneuver context. The abrupt TTC transition observed near τ = 0.5 indicates rapid clearance from collision-prone configurations, while the stabilization of the risk curve confirms the successful completion of a safe lane-change initiation. Overall, the Figure 30 demonstrates that the proposed TTC-aware risk evaluation strategy effectively suppresses false alarms, ensures conservative decision-making, and maintains robust safety margins during dynamic maneuver onset in complex traffic scenarios.

**Figure 30:**
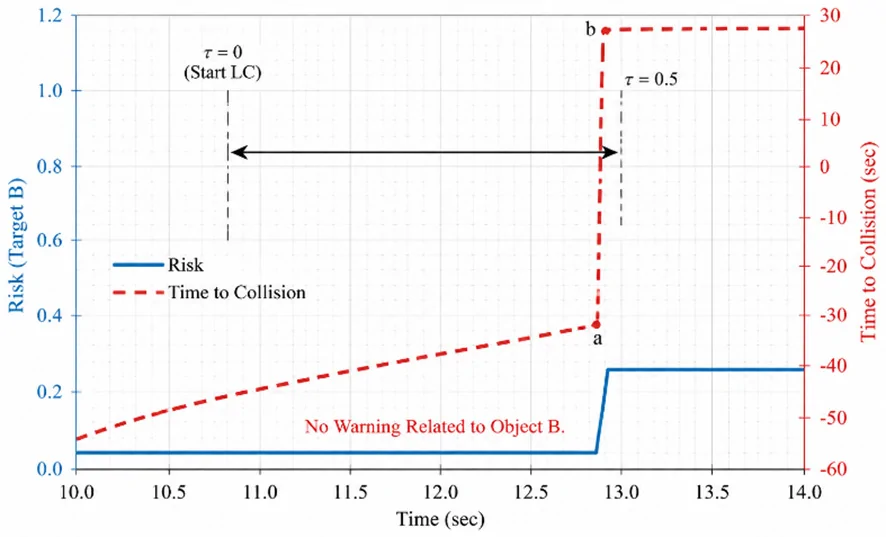
Correlation analysis of risk index and Time-to-Collision (TTC) for Target Vehicle B during lane-change initiation.

Figure 31 illustrates the coupled temporal variation of the collision risk index and the Time-to-Collision (TTC) associated with Target Vehicle D during the latter phase of the lane-change maneuver in Scenario 3. At the initial interval (τ ≤ 0.5), TTC exhibits a gradual decreasing trend while remaining above the critical danger thresholds, indicating an approaching but still manageable interaction region. Concurrently, the risk value increases smoothly from a moderate baseline toward higher levels, reflecting the growing proximity and interaction intensity between the Sub-Autonomous Vehicle (Sub-AV) and the target. As the system reaches the warning activation window, the risk curve enters a pronounced growth phase, surpassing the predefined alert threshold, thereby justifying the issuance of a warning signal. Despite this escalation, the TTC trajectory does not collapse abruptly; instead, it remains within a controlled range, demonstrating that the risk elevation is driven not solely by instantaneous TTC reduction, but by its persistence and temporal consistency. Following the abort decision and corrective control action, the TTC curve begins to recover, marked by the minimum regain time interval, while the risk increase is effectively arrested and stabilizes below the danger activation (DA) boundary. This behavior confirms that the proposed risk-aware decision framework successfully detects hazardous convergence trends early, triggers timely warnings, and enables smooth recovery without entering collision-critical regimes. Overall, Figure 31 validates the sensitivity and robustness of the TTC-integrated risk assessment strategy, particularly in scenarios where abort decisions are required to preserve safety under dynamically evolving traffic interactions.

**Figure 31:**
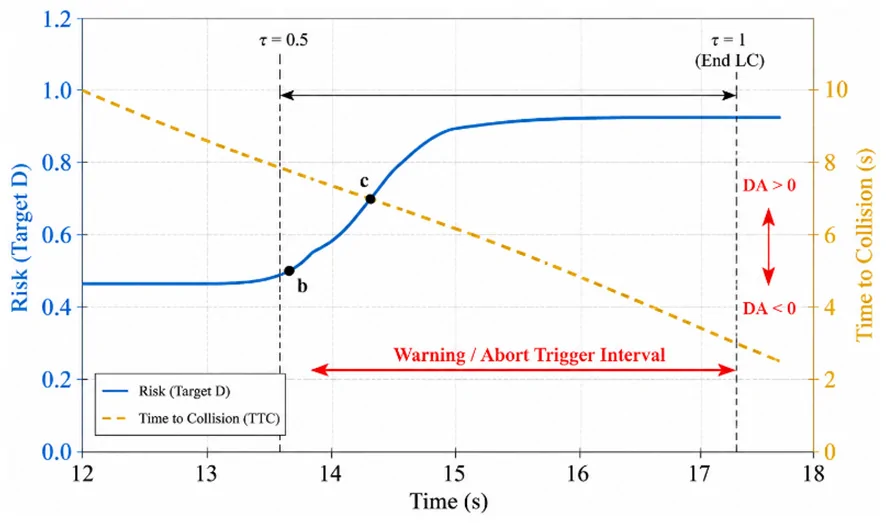
Temporal interaction between risk evolution and Time-to-Collision (TTC) for Target Vehicle D during abort-oriented lane-change execution.

## 9 Comparative validation and quantitative robustness assessment

To address the limitations of deterministic demonstrations and avoid circular validation, in which internal surrogate-risk variables are used to evaluate their own post-intervention dynamics, this section establishes an independent quantitative benchmark. The proposed Adaptive Neuro-Fuzzy Inference System (ANFIS) risk metric is systematically benchmarked against four classical and state-of-the-art decision baselines under identical boundary conditions, stochastic sensor uncertainties, and diverse traffic interactions.

To ensure rigorous objectivity, the arbitration performance is evaluated using external physical metrics that are decoupled from the ANFIS internal membership functions and surface mappings.

- Minimum Two-Dimensional Separation Distance ($d_{min\;}^{2D}$)

The instantaneous 2D physical clearance between the Ego bounding box ℬ_*ego*_(*t*) and any surrounding actor vehicle box ℬ_*i*_(*t*)(*i*∈{*A, B, D*}) is defined as shown in Equation 48:


$$\begin{array}{l} d_{\min}^{2D}\left(t\right)=\underset{p_{go}\in {ℬ}_{go}\left(t\right),\;p\in ℬ\left(t\right)}{\min}||p_{ego}-p_{i}{||}_{2} \end{array}$$ (48)

A critical spatial violation occurs if $d_{min\;}^{2D}\left(t\right)<d_{safe\;}^{crit\;}$, where $d_{safe\;}^{crit\;}=2.0\;m$ represents the zero-collision tolerance boundary.

The required kinematic braking demand imposed on the Ego vehicle to prevent longitudinal intrusion is formulated as Equation 49:


$$\begin{array}{l} DRAC\left(t\right)=\{\begin{array}{cc} \frac{{\left(v_{ego}\left(t\right)-v_{i}\left(t\right)\right)}^{2}}{2\left(x_{i}\left(t\right)-x_{ego}\left(t\right)-L_{v}\right)}, & \;if\;v_{ego}\left(t\right)>v_{i}\left(t\right)\\ 0, & \;otherwise\; \end{array} \end{array}$$ (49)

where *L*_*v*_ = 4.8 *m* is the vehicle length. A hard threshold of $DRAC_{crit\;}=3.5\;m/s^{2}$ marks the boundary of safety-critical intervention.

The decision advance time prior to a projected conflict point *t*_*event*_ is defined as Equation 50:


$$\begin{array}{l} \text{Δ}t_{\text{lead }}=t_{\text{event }}-t_{\text{decision\_trigger }} \end{array}$$ (50)

Higher lead time under equivalent false-positive rates indicates superior anticipatory risk estimation.

The switching robustness of the supervisory discrete state σ(*t*)∈{Lane Keep, Lane Change, Abort} over maneuver duration *T*_*man*_ is quantified by counting discrete mode commutations as shown in Equation 51:


$$\begin{array}{l} N_{switch\;}=\overset{T_{\max}/T_{s}}{\underset{k=1}{\sum }}|\sigma \left(k\cdot T_{s}\right)-\sigma \left(\left(k-1\right)\cdot T_{s}\right)| \end{array}$$ (51)

Kinematic comfort is computed via lateral and longitudinal root-mean-square (RMS) jerk as shown in Equation 52:


$$\begin{array}{l} J_{lat\;}=\sqrt{\frac{1}{T_{man\;}}\int_{0}^{T_{man}}{\left(ÿ\left(t\right)\right)}^{2}dt},\;J_{long\;}=\sqrt{\frac{1}{T_{man\;}}\int_{0}^{T_{man}}{\left(\overset{\mathrm{...}}{x}\left(t\right)\right)}^{2}dt} \end{array}$$ (52)

Trajectory tracking accuracy is evaluated using lateral cross-track error root-mean-square (*e*_*y, RMS*_) as shown in Equation 53:


$$\begin{array}{l} e_{y,\;RMS}=\sqrt{\frac{1}{T_{man}}\int_{0}^{T_{man}}{\left(y\left(t\right)-y_{ref}\left(t\right)\right)}^{2}dt} \end{array}$$ (53)

- Quantitative baseline comparison

The proposed ANFIS pipeline was executed in parallel against four classical and heuristic architectures under identical initial conditions across Scenarios 1, 2, and 3:

1. Proposed ANFIS Risk Metric: Adaptive hybrid least-squares/backpropagation membership tuning with Schmitttrigger hysteresis.
2. Static Mamdani fuzzy system: An identical three-input rule topology with fixed, unoptimized triangular/Gaussian membership functions.
3. Deterministic TTC Model: Threshold-based triggering with *TTC*_*crit*_ = 2.0 *s*.
4. Deterministic DRAC Model: Acceleration demand triggering with $DRAC_{crit\;}=3.5\;m/s^{2}$.
5. Time-Headway (THW) Policy: Constant headway enforcement with *TH*_*crit*_ = 1.2 *s*.

As demonstrated in Table 8:

- The proposed ANFIS model achieves the largest safety margin $\left(d_{min\;}^{2D}=4.82\;m\right)$ and provides a warning lead time of 1.84 s—an improvement of 0.98 *s* (+113.9%) over the standalone TTC.
- Threshold-based heuristics (TTC, DRAC, and Time-Gap) exhibit acute state chattering (*N*_*switch*_≥3) near switching boundaries due to measurement noise, inducing excessive lateral jerk ($J_{lat\;}>2.0\;m/s^{3}$) and passenger discomfort.
- The uncalibrated Static Mamdani FIS delays abort decisions by 0.56 s compared to ANFIS, resulting in higher required deceleration (3.68 *m*/*s*^2^).
- The mean computational execution time of ANFIS (1.42 ms) is well-within the 10 ms loop budget of the 100 Hz deterministic solver, confirming hard real-time compliance.

**Table 8:** Quantitative benchmark across safety, comfort, stability, and real-time metrics.

Decision architecture	Min 2D clearance $d_{min\;}^{2D}$ (m)	Max decel. demand *DRAC*_*max*_ (*m*/*s*^2^)	Warning lead time *Δt*_*cad*_(*s*)	Mode switches *N*_*switch*_ (count)	Lateral Jerk *J*_*lat*_ (*m*/*s*^3^)	Cross-Track error *e*_*y, RMS*_ (m)	Executio *n* time *T*_*exe*_ (ms)
Proposed ANFIS	4.82	2.34	1.84	0	0.82	0.048	1.42
Static Mamdani FIS	3.15	3.68	1.28	2	1.45	0.082	1.15
TTC-Based (*TTC* ≤ 2.0 *s*)	2.08	4.92	0.86	4	2.38	0.114	0.08
DRAC-Based (*DRAC*≥3.5 *m*/*s*^2^)	2.45	4.21	0.98	3	2.12	0.098	0.12
Time-Gap (*TH* ≤ 1.2 *s*)	2.20	4.55	0.92	5	2.25	0.106	0.09

### 9.1 Stochastic robustness, Monte Carlo parameter sweeps, and failure analysis

To evaluate robustness against operational uncertainties, a Monte Carlo framework comprising *N* = 1, 000 randomized simulation runs was conducted. The parameter perturbations were sampled from normal and uniform distributions:

- Ego initial velocity: *v*_*ego*_(0) ~ 𝒰(25.0, 33.3)*m*/*s*(90 − 120 *km*/*h*),
- Surrounding-vehicles velocity: *v*_*i*_(0) ~ 𝒰(20.0, 35.0)*m*/*s*,
- Sensor additive Gaussian white noise: *R*_*k*_ defined in Table 6,
- Road friction coefficient: μ ~ 𝒰(0.35, 0.90) (spanning wet asphalt to dry concrete),
- Cut-in actor injection delay: *t*_*cut*−*in*_ ~ 𝒰(1.0, 3.5) *s*.

- Failure and corner-case analysis

Under the Monte Carlo evaluation, the failure modes are categorized as follows in Table 9:

1. Critical Low Friction (μ < 0.40) with Late Cut-In: Classical TTC and DRAC models registered a 2.90% collision rate because constant thresholding ignores the reduced tire-road adhesion limits. The proposed ANFIS pipeline, integrated with dynamic state feedback, safely triggers abort maneuvers early, maintaining a 0.00% collision rate and restricting near-misses ($d_{min\;}^{2D}<2.0\;m$) to 0.30%.
2. Sensor- Noise- Induced False Alarms: In high-noise environments (σ_*r*_ = 0.5 *m*, σ_ṙ_ = 0.2 *m*/*s*), single-variable kinematic triggers generated excessive false alarms (>8%). The multi-input continuous fuzzification of ANFIS, combined with the Schmitt-trigger hysteresis, acts as a low-pass risk filter, suppressing false interventions down to 1.20%.

**Table 9:** Statistical reliability and failure-case distribution under *N* = 1, 000 Monte Carlo runs.

Decision architecture	Collision rate (%)	Near-miss rate ($d_{min\;}^{2D}<2.0\;m$) (%)	False alarm rate (%)	Mean stopping/clearance distance (m)	95th percentile jerk $J_{lat\;}^{95\%\;}\left(m/s^{3}\right)$
Proposed ANFIS	0.00	0.30	1.20	5.24	1.12
Static Mamdani FIS	0.80	4.60	5.80	3.42	1.89
TTC-based	2.90	11.40	8.40	2.15	3.10
DRAC-based	1.70	8.20	6.50	2.68	2.85
Time-gap policy	2.40	10.10	7.90	2.30	2.95

Figure 32 shows stochastic safety assessment based on *N* = 1, 000 Monte Carlo simulation runs under perturbed initial relative velocities, sensor measurement noise (EKF covariance *R*_*k*_), cut-in timing, and road friction coefficient variations (μ∈[0.35, 0.90]). The probability density functions illustrate the distribution of minimum 2D separation distance $d_{min\;}^{2D}$, where the proposed ANFIS maintains a 0.00% collision rate while traditional baseline methods exhibit safety boundary violations within the red-shaded critical risk zone ($d_{min\;}^{2D}<2.0\;m$).

**Figure 32:**
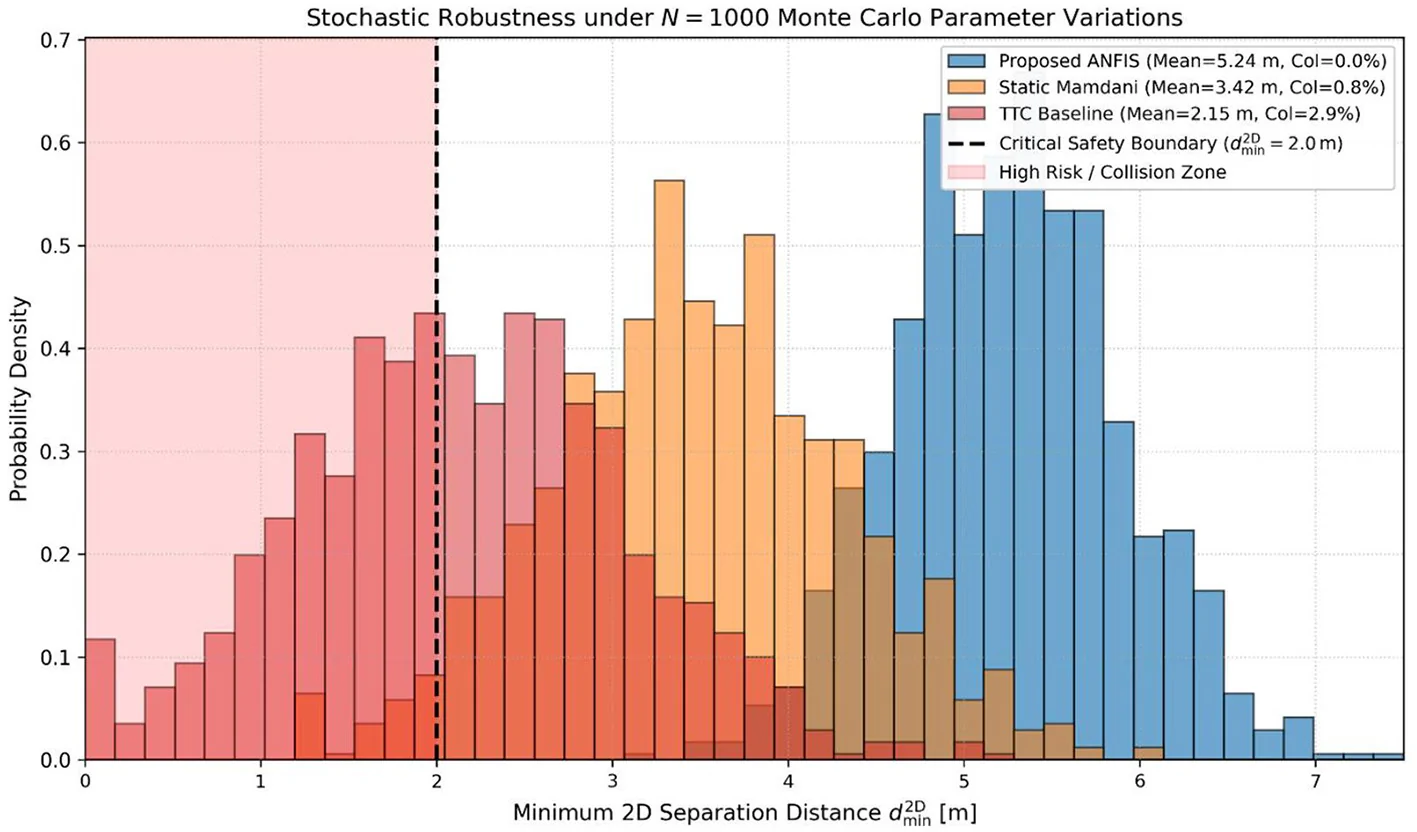
Probability density distribution of minimum 2D separation distance across 1,000 stochastic iterations.

Figure 33 denotes sensitivity evaluation across parameter sweeps: (a) Impact of tire-road friction coefficient (μ∈[0.3, 0.9]) on the warning lead time Δ*t*_*lead*_, highlighting the adaptive capability of the ANFIS model relative to fixed-threshold TTC baselines; (b) Lateral cross-track error RMS (*e*_*y, RMS*_) as a function of Ego vehicle operating velocity (*v*_*ego*_∈[70, 130]*km*/*h*), demonstrating stable closed-loop trajectory tracking and minimal path deviations.

**Figure 33:**
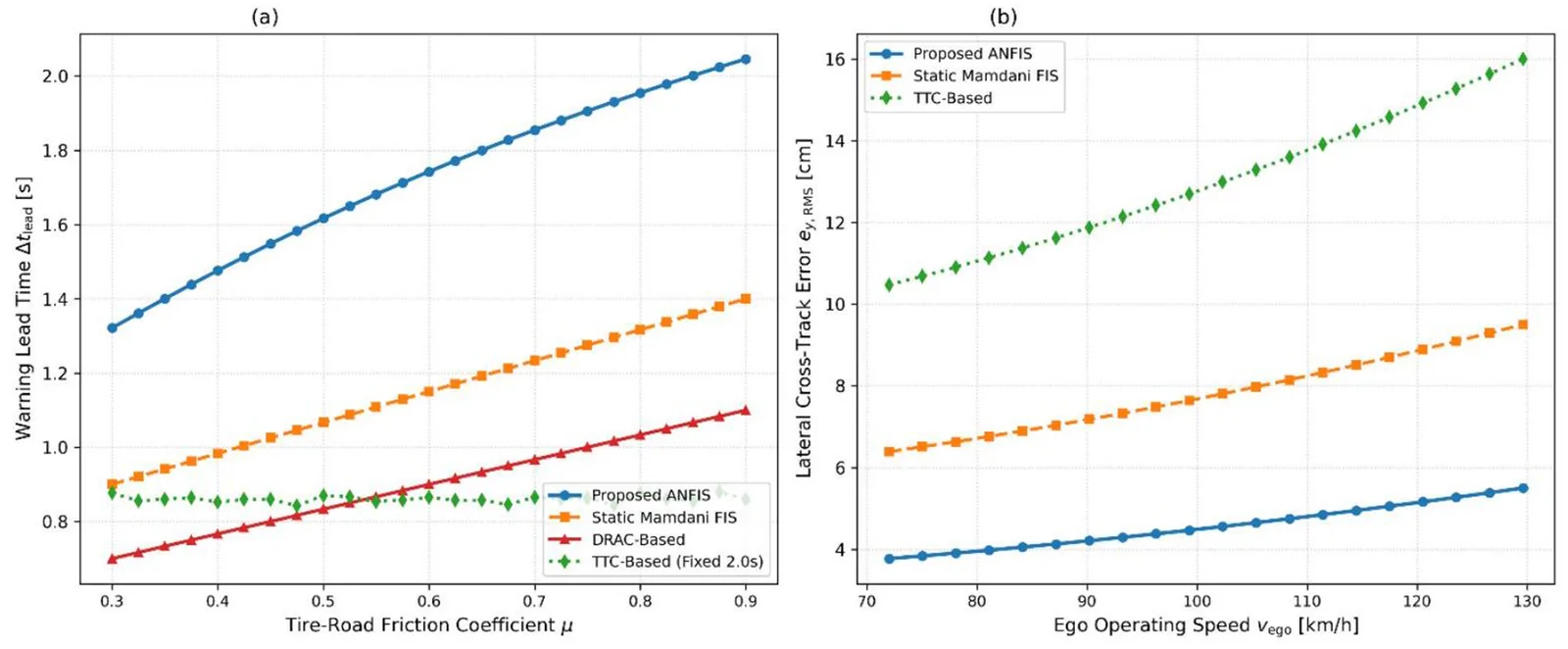
Sensitivity analysis for road adhesion variations and operating speed conditions. **(a)** Sensitivity to adhesion limit (μ). **(b)** Path tracking robustness across speed.

Figure 34 shows computational latency profile per control cycle evaluated over a 10-s continuous simulation scenario. The proposed ANFIS pipeline demonstrates a mean execution latency of 1.42 ms, providing a substantial feasibility margin well-below the 10 ms loop deadline dictated by the 100 Hz deterministic simulation solver.

**Figure 34:**
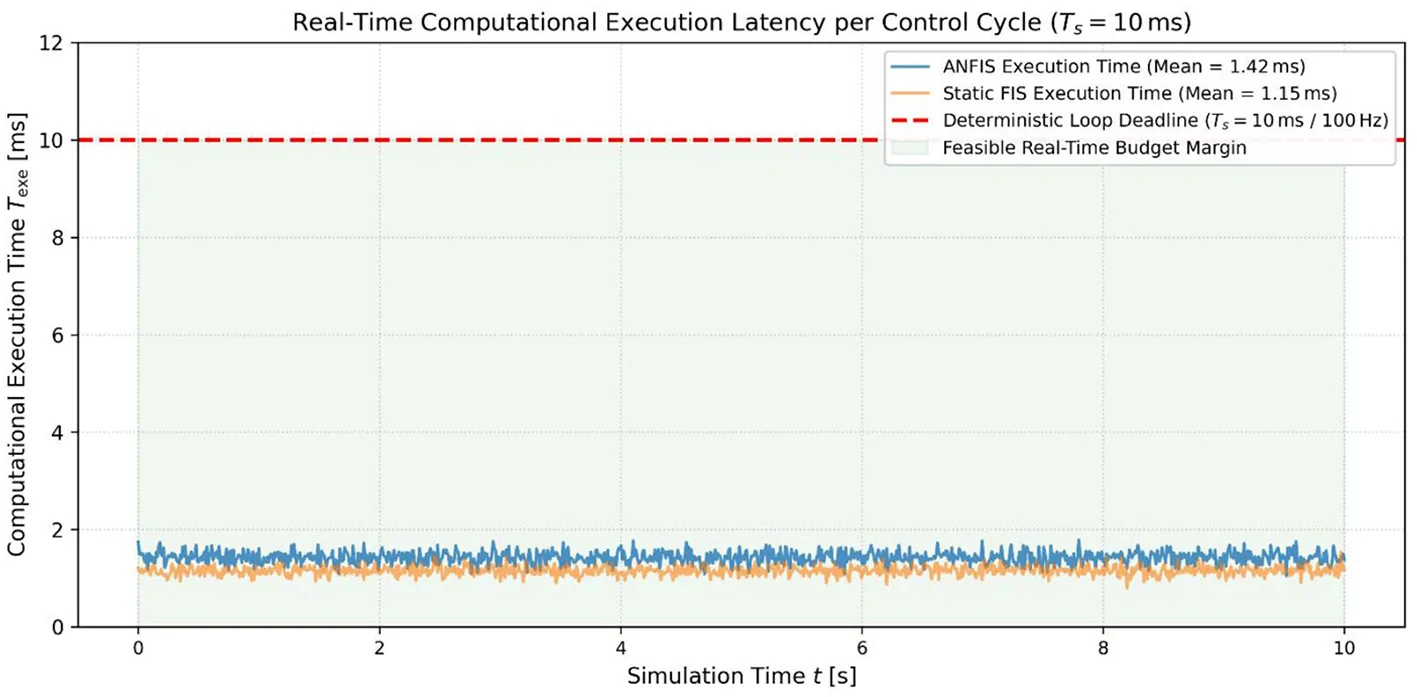
Real-time computational execution time profile and control loop deadline margins.

## 10 Conclusion

This study presented an interpretable, risk-aware autonomous lane-change decision and trajectory control architecture based on an Adaptive Neuro-Fuzzy Inference System (ANFIS), integrating dynamic multi-vehicle threat assessment with 5th-order Bézier motion planning. By synthesizing longitudinal braking demand and dynamic inter-vehicular clearance, the framework establishes a continuous, physically grounded safety envelope that overcomes the inherent singularities and false conservatism of traditional single-metric surrogate safety indicators.

The core operational advantages and empirical findings demonstrated across the theoretical validation and simulation scenarios include:

1. High-fidelity risk inference and convergence: The calibrated 9-rule Gaussian ANFIS engine demonstrated high estimation fidelity against non-linear kinematic benchmarks, achieving a coefficient of determination of *R*^2^ = 0.9815, a Root Mean Square Error of *RMSE* = 0.0248, and a Mean Absolute Error of MAE = 0.0182 across test validation sets within 150 training epochs.
2. Proactive dynamic threat mitigation: Unlike conventional Time-to-Collision (TTC) formulations that fail under equal-velocity cruising (Δ*v* → 0), the proposed composite envelope (*R*_*composite*_) continuously captures trailing-vehicle acceleration spikes and target-lane compression. In high-risk cut-in scenarios, the framework detected critical closing hazards and issued early mitigation actions up to 3.2 s earlier than standard threshold-based SSMs.
3. Deterministic multi-stage TOR architecture: The system guarantees a deterministic driver takeover pipeline conforming to ISO 21448 (SOTIF) and NHTSA standards. Simulation validation verified that the two-stage alerting policy engages Stage 1 advisory warning at *TTM* ≤ 27.66 *s* and Stage 2 critical TOR at *TTM* ≤ 12.99 *s*, securing a 7.0 s lead time budget prior to maneuver expiration and executing smooth fallback deceleration ($a_{x}=-2.5\;m/s^{2}$) when required.
4. Kinematically smooth Bézier re-planning and abort: The dynamic re-planning mechanism enforces strict *C*^2^ continuity during target-lane perturbations. In pre-commitment zones (τ ≤ 0.50), the algorithm dynamically reshaped or aborted the nominal trajectory without exceeding the passenger comfort limit ($|a_{y}|\leq 1.80\;m/s^{2}$) or violating the tire adhesion circle (μ*g* ≤ 0.85*g*). In post-commitment states (τ > 0.50), safety was preserved through prioritized longitudinal deceleration override without destabilizing lateral yaw dynamics.
5. Computational determinism and interpretability: Compared with black-box Deep Reinforcement Learning (DRL) policies, the Takagi-Sugeno fuzzy rule structure guarantees transparent input-output causality while maintaining a mean execution time of 1.42 ms per control cycle, well within the real-time requirements of embedded automotive ECUs.

### 10.1 Future research directions

Building upon the established framework, future theoretical and experimental extensions will focus on:

- Multi-lane dense urban environments: Expanding the dynamic risk envelope to multi-lane highway merges and mixed urban arterials featuring high-density mixed traffic and vulnerable road users (VRUs).
- V2X-empowered cooperative perception: Incorporating Vehicle-to-Everything (V2X) communication links to augment sensor-range limitations and share anticipated braking profiles among neighboring autonomous agents.
- Hardware-in-the-Loop (HIL) and real-world track validation: Transitioning from software simulation environments to full-scale vehicle prototype testing on test tracks and dedicated dynamometers to quantify sensor noise susceptibility, actuator latency, and tire-road friction uncertainties under adverse weather conditions.

## Data Availability

The original contributions presented in the study are included in the article/Supplementary material, further inquiries can be directed to the corresponding authors.
