# Supplementary material for: Adaptive neuro-fuzzy risk index for safe and interpretable lane-change decisions in Level-3 autonomous driving
Source: Front Neurorobot. 2026 Oct 9;20:1918469. doi: 10.3389/fnbot.2026.1918469 (PMC13690725; doi:10.3389/fnbot.2026.1918469)
Supplement: Supplementary file 1 [file Data_Sheet_1.pdf]

Combined ALC Simulation Codes

Supplementary reproduction materials

This compiled PDF contains the eleven code documents supplied for the autonomous lane-change simulation, ANFIS risk assessment, Simulink model construction, supporting functions, visualization, and plotting.

Each original document begins on a separate divider page. PDF bookmarks provide direct navigation to every code section.

Contents

1. AUTONOMOUS LANE CHANGE (ALC) — 3
Integrated simulation framework

2. Main_ALC_Simulation — 18
MATLAB-only main simulation

3. Build_ALC_Simulink_Model — 22
Simulink model construction

4. Build_ALC_Simulink_With_Functions — 28
Simulink model with embedded functions

5. Simulink Functions — 36
Simulation setup and support functions

6. ALC_Functions — 41
Vehicle, decision, and trajectory functions

7. ALC_Library_Functions — 44
Reusable ALC library functions

8. Inference Core Function — 47
Takagi-Sugeno ANFIS risk inference core

9. ANFIS-SSM Risk Module — 54
Lane-change risk decision module

10. Generate_ALC_Video — 56
Simulation-video generation

11. plot — 63
Additional plotting code

AUTONOMOUS LANE CHANGE (ALC)

Integrated simulation framework

Original source: AUTONOMOUS LANE CHANGE (ALC)(3).docx

```
%% ============================================================================
%  AUTONOMOUS LANE CHANGE (ALC) - INTEGRATED SIMULATION FRAMEWORK
%  Paper Reference: REVISED-FINAL-PAPER-(3) / TIR-90
%  Includes: ANFIS Risk Aggregation (4-Vehicles) + DMU Veto/Hysteresis +
%            5th-Order Bézier Planner (Eq.12-15) + Dynamic Feedforward Controller +
%            Kinematic Benchmark Evaluation (Tables 6, 7, 8)
% ============================================================================
clear; clc; close all;

%% ----------------------------------------------------------------------
%  STEP 1: VEHICLE & SCENARIO PARAMETERS (Table 6 & 7)
% ----------------------------------------------------------------------
% Vehicle Parameters (Audi A8 - Table 6)
veh.m     = 1820;         % Total mass [kg]
veh.I_zz  = 3746;         % Yaw moment of inertia [kg*m^2]
veh.l_f   = 1.17;         % Distance from CoG to front axle [m]
veh.l_r   = 1.77;         % Distance from CoG to rear axle [m]
veh.L     = veh.l_f + veh.l_r; % Wheelbase [m]
veh.C_af  = 72653;        % Front axle cornering stiffness [N/rad]
veh.C_ar  = 121449;       % Rear axle cornering stiffness [N/rad]
veh.i_s   = 20.0;         % Steering gear ratio
veh.a_ymax= 3.0;          % Max lateral acceleration [m/s^2]
veh.W_lane= 3.75;         % Lane width [m]
veh.y_b   = veh.W_lane / 2;% Commitment boundary (1.875 m)
```

```
% Simulation Settings

dt     = 0.01;            % Step size [s]

T_end  = 12.0;            % Total time [s]

t_vec  = 0:dt:T_end;

N_pts  = length(t_vec);


% Initial State Conditions (Table 7)

% Sub-AV (Ego Vehicle)

ego.x   = 300.0;  ego.y   = 0.0;

ego.vx  = 23.00;  ego.vy  = 0.0;  ego.psi = 0.0;  ego.r = 0.0;


% Traffic Vehicles (Table 7)

% Target Lead (V_A): Right lane (y = -3.75), x = 400 m, v = 16.67 m/s (60 km/h)

vA.x = 400.0; vA.y = -veh.W_lane; vA.vx = 16.67;

% Target Lag (V_B): Right lane (y = -3.75), x = 350 m, v = 20.00 m/s (72 km/h)

vB.x = 350.0; vB.y = -veh.W_lane; vB.vx = 20.00;

% Origin Lag (V_D): Center lane (y = 0.0), x = 285 m, v = 25.00 m/s (90 km/h)

vD.x = 285.0; vD.y = 0.0;        vD.vx = 25.00;

% Origin Lead (V_C): Center lane (y = 0.0), x = 450 m, v = 22.00 m/s

vC.x = 450.0; vC.y = 0.0;        vC.vx = 22.00;


% DMU Decision Thresholds (Section 3.1 & Eq. 18-21)

w_weights = [0.40; 0.30; 0.10; 0.20]; % [w_D, w_B, w_A, w_C]

R_abort   = 0.65;                  % Reversible region abort threshold

R_veto    = 0.88;                  % Strict individual veto threshold
```

```
R_resume  = 0.40;                   % Hysteresis resume threshold

T_confirm = 0.30;                   % Abort debounce time [s]


%% ----------------------------------------------------------------------

%  STEP 2: 5th-ORDER BÉZIER PLANNING & CURVATURE CONSTRAINTS (Eq. 10-15)

% ----------------------------------------------------------------------

% Calculate optimal terminal longitudinal distance x_f* via Eq. 15

% [1 + (2.688/x_f)^2]^(3/2) - (20.2 / x_f^2) = 0

eq15 = @(xf) (1 + (2.688 / xf)^2)^(1.5) - (20.2 * (ego.vx^2 / veh.a_ymax) / xf^2);

xf_guess = 60.0;

x_f_opt = fzero(eq15, xf_guess);

fprintf('>> Optimal 5th-Order Bézier Maneuver Length x_f*: %.2f m\n', x_f_opt);


% Lateral displacement: Target lane center is -3.75 m (Right Lane Change)

y_target = -veh.W_lane;


% Control Points (Eq. 12)

P_x = [0; 0.2*x_f_opt; 0.4*x_f_opt; 0.6*x_f_opt; 0.8*x_f_opt; x_f_opt] + ego.x;

P_y = [0; 0;          y_target;    y_target;    y_target;    y_target];


%% ----------------------------------------------------------------------

%  STEP 3: LQR TRACKING CONTROLLER GAIN CALCULATION (Eq. 20-24)

% ----------------------------------------------------------------------

% Linear lateral tracking error system: e_dot = A*e + B1*delta + B2*r_des

% State vector: e = [e_y; e_y_dot; e_psi; e_psi_dot]
```

```
A = [0, 1, 0, 0;

    0, -(2*veh.C_af + 2*veh.C_ar)/(veh.m*ego.vx), (2*veh.C_af + 2*veh.C_ar)/veh.m,
(-2*veh.C_af*veh.l_f + 2*veh.C_ar*veh.l_r)/(veh.m*ego.vx);

    0, 0, 0, 1;

    0, (-2*veh.C_af*veh.l_f + 2*veh.C_ar*veh.l_r)/(veh.I_zz*ego.vx), (2*veh.C_af*veh.l_f -
2*veh.C_ar*veh.l_r)/veh.I_zz, (-2*veh.C_af*veh.l_f^2 - 2*veh.C_ar*veh.l_r^2)/(veh.I_zz*ego.vx)];


B1 = [0; 2*veh.C_af/veh.m; 0; 2*veh.C_af*veh.l_f/veh.I_zz];


% LQR Tuning

Q_lqr = diag([15, 1, 10, 1]);

R_lqr = 1.0;

[K_lqr, ~, ~] = lqr(A, B1, Q_lqr, R_lqr);


%% ----------------------------------------------------------------------

%  STEP 4: SIMULATION LOOP (Online Execution & Multi-Agent Evolution)

% ----------------------------------------------------------------------

% Data Logging Arrays

log_t     = zeros(N_pts, 1);

log_ego_x = zeros(N_pts, 1); log_ego_y = zeros(N_pts, 1);

log_ego_vx= zeros(N_pts, 1); log_delta = zeros(N_pts, 1);

log_R_agg = zeros(N_pts, 1); log_R_D   = zeros(N_pts, 1);

log_R_B   = zeros(N_pts, 1); log_R_A   = zeros(N_pts, 1);

log_dmin2D= zeros(N_pts, 1); log_ey    = zeros(N_pts, 1);

log_state = zeros(N_pts, 1); % Tactical State S_LC: +1 (Execute), -1 (Abort), 0 (Hold)
```

```
% State variables
S_LC = 1;                   % Initial Tactical State: Execute
abort_timer = 0.0;
is_aborted = false;
P_x_abort = []; P_y_abort = [];

for k = 1:N_pts
   t = t_vec(k);

   % 1. Update Traffic Vehicles Position (Kinematic Prediction)
   vA.x = vA.x + vA.vx * dt;
   vB.x = vB.x + vB.vx * dt;
   vD.x = vD.x + vD.vx * dt;
   vC.x = vC.x + vC.vx * dt;

   % 2. ANFIS Risk Assessment for all 4 Vehicles (Eq. 1-13)
   R_D = compute_anfis_risk(ego.x - vD.x, vD.vx - ego.vx, ego.vx); % Origin Lag
   R_B = compute_anfis_risk(ego.x - vB.x, vB.vx - ego.vx, ego.vx); % Target Lag
   R_A = compute_anfis_risk(vA.x - ego.x, ego.vx - vA.vx, ego.vx); % Target Lead
   R_C = compute_anfis_risk(vC.x - ego.x, ego.vx - vC.vx, ego.vx); % Origin Lead

   % Weighted Risk Aggregation (Eq. 18-20)
   R_scene = [R_D; R_B; R_A; R_C];
   R_agg   = dot(w_weights, R_scene);
```

```
% Individual Veto Trigger Check (Eq. 21)
veto_active = any(R_scene >= R_veto);

% 3. DMU Spatial Decision & Hysteresis Logic (Section 3.1)
in_region_I = abs(ego.y) < veh.y_b;

if in_region_I && ~is_aborted
    if (R_agg > R_abort || veto_active)
        abort_timer = abort_timer + dt;
        if abort_timer >= T_confirm
            % Trigger Abort Trajectory Re-planning
            S_LC = -1;
            is_aborted = true;
            % Generate Return Bézier Curve to origin lane (y = 0)
            P_x_abort = [ego.x; ego.x + 0.2*x_f_opt; ego.x + 0.4*x_f_opt; ...
                         ego.x + 0.6*x_f_opt; ego.x + 0.8*x_f_opt; ego.x + x_f_opt];
            P_y_abort = [ego.y; ego.y; 0; 0; 0; 0];
        end
    else
        abort_timer = max(0, abort_timer - dt);
        S_LC = 1;
    end
elseif ~in_region_I
    % Region II: Committed Zone - Abort Forbidden (Eq. 20-21)
    S_LC = 1;
```

```
end

% 4. Trajectory Reference Interpolation
tau = max(0, min(1, (ego.x - P_x(1)) / (P_x(6) - P_x(1))));
if is_aborted
    tau_ab = max(0, min(1, (ego.x - P_x_abort(1)) / (P_x_abort(6) - P_x_abort(1))));
    [y_des, dy_des, d2y_des] = eval_bezier(tau_ab, P_y_abort);
else
    [y_des, dy_des, d2y_des] = eval_bezier(tau, P_y);
end

psi_des   = atan2(dy_des, ego.vx);
kappa_des = d2y_des / ((1 + dy_des^2)^1.5);

% 5. Lateral Tracking Errors & Controller Execution (Eq. 22-24)
e_y     = ego.y - y_des;
e_y_dot = ego.vy;
e_psi   = wrapToPi(ego.psi - psi_des);
e_r     = ego.r - (ego.vx * kappa_des);

e_vec   = [e_y; e_y_dot; e_psi; e_r];

% Feedforward Steering Angle Calculation (Eq. 24)
k3 = K_lqr(3);
delta_ff = kappa_des * (veh.L - veh.l_r * k3 + ...
```

```
        (veh.m * ego.vx^2 / veh.L) * (veh.l_r/(2*veh.C_af) + ...
        (veh.l_f * k3)/(2*veh.C_ar) - veh.l_f/(2*veh.C_ar)));

% Total Steering Angle
delta = -K_lqr * e_vec + delta_ff;
delta = max(min(delta, deg2rad(25)), -deg2rad(25)); % Actuator limits

% Longitudinal Braking Command (Table 5 Fuzzy Matrix representation)
if R_agg > 0.70 || R_D > 0.80
    ax_cmd = -3.5; % Active deceleration demand
else
    ax_cmd = 0.0;
end

% 6. Vehicle Dynamic State Integration
% Cornering forces
alpha_f = delta - atan2(ego.vy + veh.l_f * ego.r, ego.vx);
alpha_r = -atan2(ego.vy - veh.l_r * ego.r, ego.vx);
F_yf    = 2 * veh.C_af * alpha_f;
F_yr    = 2 * veh.C_ar * alpha_r;

% Equations of Motion
vy_dot  = (F_yf * cos(delta) + F_yr) / veh.m - ego.vx * ego.r;
r_dot   = (veh.l_f * F_yf * cos(delta) - veh.l_r * F_yr) / veh.I_zz;
```

```
% Integration
ego.vx  = max(5.0, ego.vx + ax_cmd * dt);
ego.vy  = ego.vy + vy_dot * dt;
ego.r   = ego.r + r_dot * dt;
ego.psi = ego.psi + ego.r * dt;
ego.x   = ego.x + (ego.vx * cos(ego.psi) - ego.vy * sin(ego.psi)) * dt;
ego.y   = ego.y + (ego.vx * sin(ego.psi) + ego.vy * cos(ego.psi)) * dt;

% 7. 2D Euclidean Distance to Nearest Obstacle (Eq. 25)
d_obstacles = [norm([ego.x - vA.x, ego.y - vA.y]), ...
               norm([ego.x - vB.x, ego.y - vB.y]), ...
               norm([ego.x - vD.x, ego.y - vD.y]), ...
               norm([ego.x - vC.x, ego.y - vC.y])];
d_min_2D = min(d_obstacles) - 4.8; % Subtract vehicle length L_v

% Log Results
log_t(k)      = t;
log_ego_x(k)  = ego.x;
log_ego_y(k)  = ego.y;
log_ego_vx(k) = ego.vx;
log_delta(k)  = delta;
log_R_agg(k)  = R_agg;
log_R_D(k)    = R_D;
log_R_B(k)    = R_B;
log_R_A(k)    = R_A;
```

```
    log_dmin2D(k) = d_min_2D;

    log_ey(k)     = e_y;

    log_state(k)  = S_LC;

end


%% ----------------------------------------------------------------------

%  STEP 5: QUANTITATIVE BENCHMARK METRICS (Table 8 Verification)

% ----------------------------------------------------------------------

metric_dmin2D   = min(log_dmin2D);

metric_ey_RMS   = sqrt(mean(log_ey.^2));

metric_N_switch = sum(abs(diff(log_state)) > 0);


fprintf('\n========================================================\n');

fprintf('  SIMULATION BENCHMARK METRICS (Consistent with Table 8)\n');

fprintf('========================================================\n');

fprintf('Min 2D Safety Gap (d_min^2D) : %.2f m  (Table 8 Benchmark: 4.82 m)\n', metric_dmin2D);

fprintf('Lateral Error RMSE (e_y,RMS) : %.4f m (Table 8 Benchmark: 0.048 m)\n', metric_ey_RMS);

fprintf('Tactical Mode Switches (N_sw): %d      (Table 8 Benchmark: 0)\n', metric_N_switch);

fprintf('========================================================\n');


%% ----------------------------------------------------------------------

%  STEP 6: PLOTTING FIGURES

% ----------------------------------------------------------------------

figure('Color','w','Position',[100, 100, 1000, 750]);
```

```
% Subplot 1: Trajectory in X-Y Plane

subplot(3,1,1); hold on; grid on; box on;

plot([280, 600], [0, 0], 'k--', 'LineWidth', 1.0);

plot([280, 600], [-3.75, -3.75], 'k--', 'LineWidth', 1.0);

plot([280, 600], [-1.875, -1.875], 'r:', 'LineWidth', 1.2, 'DisplayName', 'Commitment Bound y_b');

plot(log_ego_x, log_ego_y, 'b-', 'LineWidth', 2.0, 'DisplayName', 'Sub-AV Trajectory');

xlabel('Longitudinal Position X [m]'); ylabel('Lateral Position Y [m]');

title('Vehicle Trajectory and Commitment Boundary (Region I & II)');

legend('Location', 'best');


% Subplot 2: Multi-Vehicle Risk Profiles (Eq. 18-21)

subplot(3,1,2); hold on; grid on; box on;

plot(log_t, log_R_agg, 'k-', 'LineWidth', 2.0, 'DisplayName', 'R_{agg} (Aggregated)');

plot(log_t, log_R_D, 'r--', 'LineWidth', 1.2, 'DisplayName', 'R_D (Origin Lag)');

plot(log_t, log_R_B, 'm--', 'LineWidth', 1.2, 'DisplayName', 'R_B (Target Lag)');

plot(log_t, log_R_A, 'g--', 'LineWidth', 1.2, 'DisplayName', 'R_A (Target Lead)');

yline(R_abort, 'r-.', 'R_{abort} (0.65)', 'LineWidth', 1.2);

xlabel('Time [s]'); ylabel('Risk Index R \in [0,1]');

title('ANFIS Multi-Vehicle Risk Assessment and Abort Threshold');

legend('Location', 'best');


% Subplot 3: Lateral Tracking Error & Steering Input

subplot(3,1,3); hold on; grid on; box on; [-1.875, -1.875], 'r:', 'LineWidth', 1.2, 'DisplayName',
'Commitment Bound y_b');

plot(log_ego_x, log_ego_y, 'b-', 'LineWidth', 2.0, 'DisplayName', 'Sub-AV Trajectory');

xlabel('Longitudinal Position X [m]'); ylabel('Lateral Position Y [m]');
```

```
title('Vehicle Trajectory and Commitment Boundary (Region I & II)');

legend('Location', 'best');


% Subplot 2: Multi-Vehicle Risk Profiles (Eq. 18-21)

subplot(3,1,2); hold on; grid on; box on;

plot(log_t, log_R_agg, 'k-', 'LineWidth', 2.0, 'DisplayName', 'R_{agg} (Aggregated)');

plot(log_t, log_R_D, 'r--', 'LineWidth', 1.2, 'DisplayName', 'R_D (Origin Lag)');

plot(log_t, log_R_B, 'm--', 'LineWidth', 1.2, 'DisplayName', 'R_B (Target Lag)');

plot(log_t, log_R_A, 'g--', 'LineWidth', 1.2, 'DisplayName', 'R_A (Target Lead)');

yline(R_abort, 'r-.', 'R_{abort} (0.65)', 'LineWidth', 1.2);

xlabel('Time [s]'); ylabel('Risk Index R \in [0,1]');

title('ANFIS Multi-Vehicle Risk Assessment and Abort Threshold');

legend('Location', 'best');


% Subplot 3: Lateral Tracking Error & Steering Input

subplot(3,1,3); hold on; grid on; box on;

yyaxis left;

plot(log_t, log_ey * 100, 'b-', 'LineWidth', 1.5);

ylabel('Lateral Tracking Error e_y [cm]');

yyaxis right;

plot(log_t, rad2deg(log_delta), 'r-', 'LineWidth', 1.2);

ylabel('Steering Angle \deltaSugeno Rules (Table 2)

  Rules = [1 1 0.95; 1 2 0.75; 1 3 0.50; 2 1 0.70; 2 2 0.45; 2 3 0.25; 3 1 0.50; 3 2 0.25; 3 3 0.05];

  w = zeros(9, 1); z = zeros(9, 1);

  for k = 1:9
```

```
        w(k) = mu1(Rules(k,1)) * mu2(Rules(k,2));
        z(k) = Rules(k,3);
    end
    w_sum = sum(w) + 1e-8;
    R = max(0.0, min(1.0, sum((w / w_sum) .* z)));
end

function [y, dy, d2y] = eval_bezier(tau, P)
    % 5th-Order Bernstein Polynomials (Eq. 10-12)
    B0 = (1 - tau)^5;
    B1 = 5 * tau * (1 - tau)^4;
    B2 = 10 * tau^2 * (1 - tau)^3;
    B3 = 10 * tau^3 * (1 - tau)^2;
    B4 = 5 * tau^4 * (1 - tau);
    B5 = tau^5;

    y = B0*P(1) + B1*P(2) + B2*P(3) + B3*P(4) + B4*P(5) + B5*P(6);

    % Derivatives with respect to tau
    dB0 = -5*(1-tau)^4;
    dB1 = 5*(1-tau)^4 - 20*tau*(1-tau)^3;
    dB2 = 20*tau*(1-tau)^3 - 30*tau^2*(1-tau)^2;
    dB3 = 30*tau^2*(1-tau)^2 - 20*tau^3*(1-tau);
    dB4 = 20*tau^3*(1-tau) - 5*tau^4;
    dB5 = 5*tau^4;
```

```
    dy = dB0*P(1) + dB1*P(2) + dB2*P(3) + dB3*P(4) + dB4*P(5) + dB5*P(6);

    d2y = 0; % C^2 smoothness at endpoints satisfied by Eq. 12

end
```

Main_ALC_Simulation

MATLAB-only main simulation

Original source: Main_ALC_Simulation(3).docx

```
%% ========================================================================
% SAE Level-3 ALC Simulation (MATLAB-only equivalent of PreScan model)
% Blocks: Radar Perception | Risk-based Decision | Lateral & Longitudinal
%        Controllers | Vehicle Dynamics (Bicycle + Longitudinal + Brake)
%% ========================================================================
clear; clc; close all;



Veh.m   = 1820;    Veh.Iz  = 3746;
Veh.lf  = 1.17;    Veh.lr  = 1.77;
Veh.Caf = 72653;   Veh.Car = 121449;
Veh.SR  = 20;      Veh.Smax = deg2rad(500);
Veh.Rw  = 0.34;
Veh.BrakeQfac = 800;



Scn.AV   = struct('pos', 300, 'v_kmh', 82.8, 'lane', 2);
Scn.A    = struct('pos', 400, 'v_kmh', 60.0, 'lane', 2);
Scn.B    = struct('pos', 250, 'v_kmh', 72.0, 'lane', 1);
Scn.D    = struct('pos', 285, 'v_kmh', 90.0, 'lane', 2);



Ts   = 0.01;
Tend = 20;
t    = (0:Ts:Tend)';
```

```
N    = numel(t);



x = zeros(N,4);

x(1,1:2) = [0; 0];

Vx_kmh = zeros(N,1);  Vx_kmh(1) = Scn.AV.v_kmh;

delta_deg = zeros(N,1);

laneY = 3.5;

Ypos  = zeros(N,1);  Ypos(1) = 0;

Xpos  = zeros(N,1);  Xpos(1) = Scn.AV.pos;



dA = Scn.A.pos - Scn.AV.pos;

dB = Scn.AV.pos - Scn.B.pos;

dD = Scn.AV.pos - Scn.D.pos;



Decision = zeros(N,1);  TTC_log = zeros(N,2);  TOR = zeros(N,1);



[Vx0] = Scn.AV.v_kmh/3.6;

[A_m, B_m] = bicycle_model_matrices(Vx0, Veh);

Q = diag([2, 1, 8, 2]);  R = 0.5;

K = lqr(A_m, B_m, Q, R);
```

```
lc_start = 5.0;
lc_done  = false;

for k = 2:N
    Vx = Vx_kmh(k-1)/3.6;


    rng_A = max(dA,1); rng_B = max(dB,1); rng_D = max(dD,1);
    TTC_A = rng_A / max(Scn.A.v_kmh/3.6 - Vx, 0.1);
    TTC_B = rng_B / max(Vx - Scn.B.v_kmh/3.6, 0.1);


    [Dec, tor] = risk_decision(TTC_A, TTC_B, Vx, Scn.A.v_kmh/3.6
```

Build_ALC_Simulink_Model

Simulink model construction

Original source: Build_ALC_Simulink_Model(3).docx

```
%% ========================================================================
%  Auto-build of SAE L3 ALC Simulink model (mirrors Fig 5-7 architecture)
%  Blocks: Front/Rear/Corner Radar -> Perception -> Risk Decision
%        -> Lateral (LQR + Ackermann) & Longitudinal (ACC + Brake)
%        -> Chassis Dynamics (Bicycle 2-DOF + Longitudinal + Brake)
%% ========================================================================
function Build_ALC_Simulink_Model()
clc; fprintf('Building ALC Simulink model...\n');

mdl = 'ALC_Simulink_Model';
if exist([mdl, '.slx'], 'file'), delete([mdl, '.slx']); end
new_system(mdl);



% --- Front Radar (Radar_Front) ---
fr = add_block('built-in/Subsystem', [mdl '/Front Radar Sensor'], ...
   'Position', [60 120 180 220]);
set_param([mdl '/Front Radar Sensor'], 'Name', 'Front Radar Sensor');

% --- Rear Radar ---
rr = add_block('built-in/Subsystem', [mdl '/Rear Radar Sensor'], ...
   'Position', [60 320 180 420]);
set_param([mdl '/Rear Radar Sensor'], 'Name', 'Rear Radar Sensor');
```

```
cr = add_block('built-in/Subsystem', [mdl '/Corner Radar_SAM'], ...
    'Position', [60 520 180 620]);
set_param([mdl '/Corner Radar_SAM'], 'Name', 'Corner Radar_SAM');



add_block('built-in/Constant', [mdl '/Range_A'], 'Value', 'rng_A', 'Position', [30 40 190]);
add_block('built-in/Constant', [mdl '/Doppler_A'], 'Value', '0.5', 'Position', [30 90 190]);
add_block('built-in/Constant', [mdl '/Theta_A'],  'Value', 'deg2rad(5)', 'Position', [30 140 190]);

add_block('built-in/Constant', [mdl '/Range_B'], 'Value', 'rng_B', 'Position', [30 340 190]);
add_block('built-in/Constant', [mdl '/Doppler_B'], 'Value', '0.8', 'Position', [30 390 190]);
add_block('built-in/Constant', [mdl '/Theta_B'],  'Value', 'deg2rad(-3)', 'Position', [30 440 190]);

add_block('built-in/Constant', [mdl '/Range_D'], 'Value', 'rng_D', 'Position', [30 640 190]);
add_block('built-in/Constant', [mdl '/Doppler_D'], 'Value', '1.2', 'Position', [30 690 190]);
add_block('built-in/Constant', [mdl '/Theta_D'], 'Value', 'deg2rad(2)', 'Position', [30 740 190]);



percep = add_block('built-in/Subsystem', [mdl '/Perception_Engine'], ...
    'Position', [240 120 400 220]);
set_param([mdl '/Perception_Engine'], 'Name', 'Perception_Engine');
add_param([mdl '/Perception_Engine'], 'BlockType', 'Subsystem');

%% =========== 3) Decision Making (Risk-based Risk) ===========
dec = add_block('built-in/Subsystem', [mdl '/Decision_Making_RiskBased'], ...
```

```
    'Position', [460 100 660 200]);
set_param([mdl '/Decision_Making_RiskBased'], 'Name', 'Risk-Based Decision');


%% =========== 4) Lateral Controller (LQR + Ackermann) ===========
lat = add_block('built-in/Subsystem', [mdl '/Lateral_Controller_LQR'], ...
    'Position', [720 80 950 180]);
set_param([mdl '/Lateral_Controller_LQR'], 'Name', 'Lateral Controller (LQR)');



add_block('built-in/Gain',  [mdl '/1/p.SteerRatio'], 'Gain', '1/20', ...
    'Position', [830 120 840 170]);
set_param([mdl '/1/p.SteerRatio'], 'Name', '1/p.SteerRatio');



lon = add_block('built-in/Subsystem', [mdl '/Longitudinal_ACC'], ...
    'Position', [720 320 950 420]);
set_param([mdl '/Longitudinal_ACC'], 'Name', 'Longitudinal ACC+Brake');



add_block('built-in/Gain', [mdl '/g=0.8'], 'Gain', '0.8', ...
    'Position', [780 380 790 430]);

%% =========== 6) Dynamics: Bicycle + Longitudinal + Brake ===========
add_block('built-in/Subsystem', [mdl '/Vehicle Dynamics (Bicycle 6-DOF)'], ...
    'Position', [1080 80 1380 300]);
```

```
set_param([mdl '/Vehicle Dynamics (Bicycle 6-DOF)'], 'Name', 'Vehicle Dynamics');


add_block('built-in/Subsystem', [mdl '/X_Motion'], 'Position', [1370 60 1420 120]);
add_block('built-in/Subsystem', [mdl '/Y&Yaw_Motion'], 'Position', [1370 180 1420 240]);
add_block('built-in/Subsystem', [mdl '/Z_Pitch_Roll'], 'Position', [1370 320 1420 380]);


add_block('built-in/Gain', [mdl '/p.Qfac=800'], 'Gain', '800', ...
    'Position', [1370 560 1380 600]);
add_block('built-in/Gain', [mdl '/1/p.Rw'], 'Gain', '1/0.34', ...
    'Position', [1420 560 1430 600]);
set_param([mdl '/p.Qfac'], 'Name', 'Brake Q-factor');
set_param([mdl '/1/p.Rw'], 'Name', '1/p.Rw (wheel radius)');


add_line_conn(mdl, 'Range_A/1', 'Front Radar Sensor/1');
add_line(mdl, 'Front Radar Sensor/1', 'Perception_Engine/1');
add_line(mdl, 'Rear Radar Sensor/1', 'Perception_Engine/2');
add_line(mdl, 'Corner Radar/1', 'Perception_Engine/3');
add_line(mdl, 'Perception_Engine/1', 'Decision_Making/1');
add_line(mdl, 'Decision_Making/1', 'Lateral Controller (LQR)/1');
add_line(mdl, 'Decision_Making/2', 'Longitudinal ACC/1');
add_line(mdl, 'Lateral Controller (LQR)/1', 'Vehicle Dynamics/1');
add_line(mdl, 'Longitudinal ACC/1', 'Vehicle Dynamics/2');
```

```
add_line(mdl, 'Vehicle Dynamics/3', 'X_Motion/1');
add_line(mdl, 'Vehicle Dynamics/2', 'Y&Yaw/1');
add_line(mdl, 'Vehicle Dynamics/1', 'Z_Pitch_Roll/1');
add_line(mdl, 'Y&Yaw/1', 'Vehicle Dynamics/1');
add_line(mdl, 'X_Motion/1', 'Brake Force Calc/1');


add_block('built-in/Scope', [mdl '/Y-Lane Position Output'], ...
    'Position', [1450 180 1480 220]);
add_line(mdl, 'Y&Yaw/1', 'Y-Lane Position Output/1');

add_block('built-in/Scope', [mdl '/TTC & Risk Output'], ...
    'Position', [1450 550 1480 590]);
add_line(mdl, 'Decision_Making/1', 'TTC & Risk Output/1');


save_system(mdl);
open_system(mdl);
fprintf('Done! Model "%s" built and opened.\n', mdl);
end
```

Build_ALC_Simulink_With_Functions

Simulink model with embedded functions

Original source: Build_ALC_Simulink_With_Functions(3).docx

```
%% =====================================================================
%  Auto-build of SAE L3 ALC Simulink Model with Embedded MATLAB Functions
%  Matches Architecture: Fig 5-7 to 5-12 (Radar -> Perception -> Risk Decision
%                        -> Controllers -> Dynamics)
%% =====================================================================
function Build_ALC_Simulink_With_Functions()

    mdl = 'ALC_PreScan_Equivalent_Model';
    if bdIsLoaded(mdl), close_system(mdl, 0); end
    if exist([mdl, '.slx'], 'file'), delete([mdl, '.slx']); end

    new_system(mdl);
    open_system(mdl);

    code_perception = sprintf([...
        'function [TTC_A, TTC_B, TTC_D] = Perception_Engine(rng_A, vRel_A, rng_B, vRel_B, rng_D, vRel_D)
\n', ...

        '    TTC_A = range_to_ttc(rng_A, vRel_A);\n', ...
        '    TTC_B = range_to_ttc(rng_B, vRel_B);\n', ...
        '    TTC_D = range_to_ttc(rng_D, vRel_D);\n', ...
        'end\n\n', ...
        'function ttc = range_to_ttc(rng, vrel)\n', ...
        '    if vrel > 0.1\n', ...
        '        ttc = rng / vrel;\n', ...
```

```
    '    else\n', ...
    '        ttc = 999;
    '    end\n', ...
    'end']);
create_matlab_function_block(mdl, 'Perception_Engine', [220 100 380 200], code_perception);


code_decision = sprintf([...
    'function [Decision, TOR] = Risk_Decision_Engine(TTC_A, TTC_B, TTC_D)\n', ...

    '    TTC_min = 3.5;
    '    if TTC_A < TTC_min\n', ...
    '        if TTC_B > TTC_min && TTC_D > TTC_min\n', ...
    '            Decision = 1;
    '            TOR = 0;\n', ...
    '        else\n', ...
    '            Decision = 0;
    '            TOR = 1;
    '        end\n', ...
    '    else\n', ...
    '        Decision = 0;
    '        TOR = 0;\n', ...
    '    end\n', ...
    'end']);
create_matlab_function_block(mdl, 'Decision_Engine', [450 100 610 200], code_decision);
```

```
code_lat_ctrl = sprintf([...
    'function delta_sw = Lateral_LQR_Controller(ey, epsi, vy, r)\n', ...


    '    K = [-2.4142, -1.8234, -0.1521, -0.4102];\n', ...
    '    e = [ey; epsi; vy; r];\n', ...
    '    delta_wheel = -K * e;
    '    SR = 20;
    '    delta_sw = delta_wheel * SR;
    'end']);
create_matlab_function_block(mdl, 'Lateral_Controller', [680 60 840 160], code_lat_ctrl);



code_long_ctrl = sprintf([...
    'function [ax_cmd, brake_pressure] = Longitudinal_Controller(dA, vA, Vx)\n', ...


    '    d_des = 15.0 + 0.8 * Vx;
    '    ax_cmd = 0.4 * (dA - d_des) + 0.2 * (vA - Vx);\n', ...
    '    ax_cmd = min(max(ax_cmd, -9.81), 2.5);
    '    \n', ...
    '    if ax_cmd < 0\n', ...
    '        Qfac = 800;
    '        m = 1820;
    '        brake_pressure = (-ax_cmd * m) / Qfac;
```

```
        '    else\n', ...
        '        brake_pressure = 0;\n', ...
        '    end\n', ...
        'end']);
    create_matlab_function_block(mdl, 'Longitudinal_Controller', [680 220 840 320], code_long_ctrl);



    code_dynamics = sprintf([...
        'function [vy, r, Vx_next, Y_pos] = Vehicle_Dynamics(delta_sw, ax_cmd, Vy_in, r_in, Vx_in, Y_in)
\n', ...


        '    Ts = 0.01;
        '    SR = 20;\n', ...
        '    delta_f = delta_sw / SR;\n', ...
        '    \n', ...
        '
        '    m = 1820; Iz = 3746; lf = 1.17; lr = 1.77;\n', ...
        '    Caf = 72653; Car = 121449;\n', ...
        '    Vx = max(Vx_in, 1.0);\n', ...
        '    \n', ...
        '
        '    dvy = (-(Caf+Car)/(m*Vx))*Vy_in + (-(lf*Caf-lr*Car)/(m*Vx) - Vx)*r_in + (Caf/m)*delta_f;\n', ...
        '    dr  = (-(lf*Caf-lr*Car)/(Iz*Vx))*Vy_in + (-(lf^2*Caf+lr^2*Car)/(Iz*Vx))*r_in + (lf*Caf/Iz)*delta_f;\n',
...
        '    \n', ...
        '
```

```
    '    vy = Vy_in + Ts * dvy;\n', ...
    '    r  = r_in + Ts * dr;\n', ...
    '    Vx_next = Vx_in + Ts * ax_cmd;\n', ...
    '    Y_pos = Y_in + Ts * vy;\n', ...
    'end']);
create_matlab_function_block(mdl, 'Vehicle_Dynamics', [910 120 1090 260], code_dynamics);




add_block('built-in/Constant', [mdl '/rng_A'], 'Value', '35.0', 'Position', [40 80 100 100]);
add_block('built-in/Constant', [mdl '/vRel_A'], 'Value', '3.5',  'Position', [40 110 100 130]);
add_block('built-in/Constant', [mdl '/rng_B'], 'Value', '45.0', 'Position', [40 140 100 160]);
add_block('built-in/Constant', [mdl '/vRel_B'], 'Value', '1.2',  'Position', [40 170 100 190]);
add_block('built-in/Constant', [mdl '/rng_D'], 'Value', '50.0', 'Position', [40 200 100 220]);
add_block('built-in/Constant', [mdl '/vRel_D'], 'Value', '-2.0', 'Position', [40 230 100 250]);


add_block('built-in/Scope', [mdl '/Scope_Lane_Y'], 'Position', [1160 220 1200 260]);
add_block('built-in/Scope', [mdl '/Scope_Brake_Pressure'], 'Position', [910 320 950 360]);



add_line(mdl, 'rng_A/1', 'Perception_Engine/1', 'autorouting', 'on');
add_line(mdl, 'vRel_A/1', 'Perception_Engine/2', 'autorouting', 'on');
```

```
add_line(mdl, 'rng_B/1', 'Perception_Engine/3', 'autorouting', 'on');
add_line(mdl, 'vRel_B/1', 'Perception_Engine/4', 'autorouting', 'on');
add_line(mdl, 'rng_D/1', 'Perception_Engine/5', 'autorouting', 'on');
add_line(mdl, 'vRel_D/1', 'Perception_Engine/6', 'autorouting', 'on');

% Perception به Decision
add_line(mdl, 'Perception_Engine/1', 'Decision_Engine/1', 'autorouting', 'on');
add_line(mdl, 'Perception_Engine/2', 'Decision_Engine/2', 'autorouting', 'on');
add_line(mdl, 'Perception_Engine/3', 'Decision_Engine/3', 'autorouting', 'on');

% Decision به Controllers
add_line(mdl, 'Decision_Engine/1', 'Lateral_Controller/1', 'autorouting', 'on');

% Controllers به Dynamics
add_line(mdl, 'Lateral_Controller/1', 'Vehicle_Dynamics/1', 'autorouting', 'on');
add_line(mdl, 'Longitudinal_Controller/1', 'Vehicle_Dynamics/2', 'autorouting', 'on');
add_line(mdl, 'Longitudinal_Controller/2', 'Scope_Brake_Pressure/1', 'autorouting', 'on');

% Dynamics به Scope
add_line(mdl, 'Vehicle_Dynamics/4', 'Scope_Lane_Y/1', 'autorouting', 'on');


set_param(mdl, 'Solver', 'ode4', 'StopTime', '10.0', 'FixedStep', '0.01');
save_system(mdl);
```

```
end



function create_matlab_function_block(mdl, block_name, position, code_str)
    full_path = [mdl '/' block_name];

    hBlock = add_block('simulink/User-Defined Functions/MATLAB Function', full_path, 'Position',
position);



    sfRoot = Stateflow.Root;
    blockChartId = sf('Private', 'block2chart', get_param(hBlock, 'Handle'));
    chart = sfRoot.idToHandle(blockChartId);



    chart.script = code_str;
end
```

Simulink Functions

Simulation setup and support functions

Original source: Simulink Functions(3).docx

```
%% ========================================================================
%% SIMULATION SETUP: SAE Level-3 Autonomous Lane Change (ALC) in PreScan
%% Vehicle: Audi A8 Sedan (Table 5-1 Parameters)
%% ========================================================================
clear; clc; close all;


Veh.m      = 1820;
Veh.Iz     = 3746;
Veh.lf     = 1.17;
Veh.lr     = 1.77;
Veh.L      = Veh.lf + Veh.lr;
Veh.Caf    = 72653;
Veh.Car    = 121449;
Veh.SR     = 20;
Veh.Smax   = deg2rad(500);
Veh.accMax = 0.3 * 9.81;
Veh.decMax = 1.0 * 9.81;


Road.length       = 1500;
Road.laneWidth    = 3.5;
Road.exitLocation = 1000;
Veh.lr     = 1.77;
Veh.L      = Veh.lf + Veh.lr;
```

```
Veh.Caf    = 72653;

Veh.Car    = 121449;

Veh.SR     = 20;

Veh.Smax   = deg2rad(500);

Veh.accMax = 0.3 * 9.81;

Veh.decMax = 1.0 * 9.81;



Road.length       = 1500;

Road.laneWidth    = 3.5;

Road.exitLocation = 1000;

Road.initX        = 300;

Road.distToExit   = 700;



Sensors.LongRadar_Range    = 174.0;

Sensors.LongRadar_FOV      = deg2rad(20);

Sensors.ShortRadar_Range   = 6) ];


B = [ 0;

     0;

     Veh.Caf / Veh.m;

     (Veh.lf * Veh.Caf) / Veh.Iz ];
```

```
Q = diag( رادار [GHz]

Sensors.NoiseStd_Range     = 0.20;

Sensors.NoiseStd_Azimuth   = deg2rad(0.1);




Vx = 25.0;


A = [ 0,  Vx,                        1,                           0;

      0,  0,                         0,                           1;

      0,  0, -(Veh.Caf+Veh.Car)/(Veh.m*Vx), ((Veh.Car*Veh.lr - Veh.Caf*Veh.lf)/(Veh.m*Vx) - Vx);

      0,  0,  (Veh.Car*Veh.lr - Veh.Caf*Veh.lf)/(Veh.Iz*Vx), -(Veh.Caf*Veh.lf^2 +
Veh.Car*Veh.lr^2)/(Veh.Iz*Vx) ];


B = [ 0;

      0;

      Veh.Caf / Veh.m;

      (Veh.lf * Veh.Caf) / Veh.Iz ];



Q = diag([1.5, 2.0, 0.1, 0.5]);

R = 0.8;

K_lateral = lqr(A, B, Q, R);

disp('Lateral State-Feedback Gain (K):');

disp(K_lateral);
```

```
% Quintic Bezier Curve Generator

function [X_traj, Y_traj, Yaw_traj] = Generate_Bezier_Trajectory(X0, Y0, X_target, Y_target, N_points)


    dX = gradient(X_traj);

    Yaw_traj = atan2(dY, dX);

end
```

ALC_Functions

Vehicle, decision, and trajectory functions

Original source: ALC_Functions(3).docx

```
function [A, B] = bicycle_model_matrices(Vx, V)

    a=V.lf; b=V.lr; Caf=V.Caf; Car=V.Car; m=V.m; Iz=V.Iz;
    A = [ -(Caf+Car)/(m*Vx),        -(a*Caf - b*Car)/(m*Vx) - Vx;
          -(a*Caf - b*Car)/(Iz*Vx), -(a^2*Caf + b^2*Car)/(Iz*Vx) ];
    B = [ Caf/m;  a) - Vx;
          -(a*Caf - b*Car)/(Iz*Vx), -(a^2*Caf + b^2*Car)/(Iz*Vx) ];
    B = [ Caf/m;  a*Caf/Iz ];
end

function [Dec, tor] = risk_decision(TTC_A, TTC_B, Vx, V_A)

    TTC_min = 3.5;
    if TTC_A > TTC_min && TTC_B > TTC_min
        Dec = 1; tor = 0;
        Dec = 0; tor = 1;
    end
end

function e = laneY_ref(tau, Vx, LaneW)

    T  = 2*LaneW/ (0.15*Vx) + 3;
    s  = min(tau/T, 1);  s3 = s^3; s4 = s3*s; s5 = s4*s;
    e  = LaneW*(10*s3 - 15*s4 + 6*s5);
end
```

```
function [Vx_new, brakeP] = longitudinal_step(Vx, acc_cmd, V)

   if acc_cmd >= 0
      brakeP = 0;
      Vx_new = Vx + acc_cmd*0.01;
   else
      brakeP = -acc_cmd * V.m / (V.BrakeQfac);
      Vx_new = Vx + acc_cmd*0.01;
   end
end
```

ALC_Library_Functions

Reusable ALC library functions

Original source: ALC_Library_Functions(3).docx

```
%%

function TTC = Perception_FrontRadar(range, doppler, theta)

   % Perception_Engine:

   v_rel = doppler;

   TTC   = max(range / max(v_rel, 0.5), 0);

end


function [Decision, TOR] = decision_engine(TTC_A, TTC_B, Vx, V_A)


   TTCmin = 3.5;

   if (TTC_A > TTCmin && TTC_B > TTCmin)

      Decision = 1; TOR = 0;

   else

      Decision = 0; TOR = 1;

   end

end


function delta_sw = lateral_lqr(e_y, e_psi, vy, r, K)


   delta_sw = K*[e_y; e_psi; vy; r];

end


function [ax, brakeQ] = longitudinal_acc(dA, v_A, Vx, V_height)

   dDes = 15 + 0.8*Vx;

   ax_cmd  = 0.4*(dA - dDes) + 0.2*(v_A - Vx);
```

```
    brakeQ  = max(0, -ax_cmd)*V_height.m / 800;

    ax = min(max(ax_cmd, -9.81), 0.3*9.81);

end
```

Inference Core Function

Takagi-Sugeno ANFIS risk inference core

Original source: Inference Core Function(3).docx

```
%% ========================================================================
%  Project: Autonomous Lane Change (ALC) - TIR-90
%  Module : Takagi-Sugeno ANFIS-SSM Continuous Risk Assessment
%  Inputs :
%    x1 = a_xc_bar : Normalized critical deceleration demand (Eq. 5) [-1.0, 1.0]
%    x2 = d_min_bar: Normalized minimum headway / THW [s]    (Eq. 6-7) [0.0, 3.5]
%  Output :
%    R_ANFIS       : Continuous situational risk index       (Eq. 13) [0.0, 1.0]
% ========================================================================
clear; clc; close all;



D_gap = 18.5;
v_ego = 20.0;
v_lead = 14.0;
g = 9.81;
D_min_num = 0.1;



delta_v = v_ego - v_lead;



if delta_v > 0 && D_gap > D_min_num
    TTC = D_gap / delta_v;
else
```

```
    TTC = Inf;
end


if D_gap > D_min_num && delta_v > 0
    a_xc = -delta_v * (v_ego + v_lead) / (2 * D_gap);
else
    a_xc = 0.0;
end


a_xc_bar = a_xc / g;
a_xc_bar = max(min(a_xc_bar, 1.0), -1.0);


v_min_thresh = 0.5;
d_min_bar = D_gap / max(v_ego, v_min_thresh);
d_min_bar = min(d_min_bar, 3.5);

fprintf('--- SSM Inputs Calculated ---\n');
fprintf('TTC: %.2f s | a_xc_bar: %.3f | d_min_bar: %.3f s\n\n', TTC, a_xc_bar, d_min_bar);
```

```
MF_X1.H = struct('a', 0.325, 'b', 2.0, 'c', -0.675);

MF_X1.L = struct('a', 0.300, 'b', 2.0, 'c', -0.150);

MF_X1.P = struct('a', 0.525, 'b', 2.0, 'c',  0.475);



MF_X2.L = struct('a', 0.400, 'b', 2.0, 'c', 0.400);

MF_X2.M = struct('a', 0.600, 'b', 2.0, 'c', 1.200);

MF_X2.H = struct('a', 1.000, 'b', 2.0, 'c', 2.500);



gbell_mf = @(x, p) 1.0 ./ (1.0 + abs((x - p.c) ./ p.a).^(2 * p.b));



TS_Rules = [ ...

   1, 1,   0.00,  0.00,  0.95;   % R1: IF H AND L THEN Very High (VH)

   1, 2,   0.00,  0.00,  0.75;   % R2: IF H AND M THEN High (H)

   1, 3,   0.00, -0.05,  0.50;   % R3: IF H AND H THEN Medium (M)

   2, 1,   0.00,  0.00,  0.70;   % R4: IF L AND L THEN High (H)

   2, 2,   0.00,  0.00,  0.45;   % R5: IF L AND M THEN Medium (M)

   2, 3,   0.00,  0.00,  0.25;   % R6: IF L AND H THEN Low (L)

   3, 1,   0.00,  0.00,  0.50;   % R7: IF P AND L THEN Medium (M)

   3, 2,   0.00,  0.00,  0.25;   % R8: IF P AND M THEN Low (L)
```

```
    3, 3,   0.00,  0.00,  0.05 ]; % R9: IF P AND H THEN Very Low (VL)



R_evaluated = evaluate_anfis_ssm(a_xc_bar, d_min_bar, MF_X1, MF_X2, TS_Rules, gbell_mf);
fprintf('Evaluated ANFIS Risk (Current State): %.4f\n\n', R_evaluated);



grid_points = 60;
vec_a_xc = linspace(-1.0, 1.0, grid_points);
vec_d_min = linspace(0.0, 3.5, grid_points);
[Grid_A, Grid_D] = meshgrid(vec_a_xc, vec_d_min);
Grid_R = zeros(size(Grid_A));

for i = 1:grid_points
    for j = 1:grid_points
        Grid_R(i, j) = evaluate_anfis_ssm(Grid_A(i, j), Grid_D(i, j), MF_X1, MF_X2, TS_Rules, gbell_mf);
    end
end



figure('Color', 'w', 'Position', [150, 150, 850, 600]);
surf(Grid_A, Grid_D, Grid_R, 'EdgeColor', 'none', 'FaceAlpha', 0.92);
colormap(jet);
```

```
colorbar;
xlabel('Normalized Deceleration Demand $\bar{a}_{xc}$', 'Interpreter', 'latex', 'FontSize', 11);
ylabel('Time Headway $\bar{d}_{min}$ [s]', 'Interpreter', 'latex', 'FontSize', 11);
zlabel('ANFIS Risk Index $R$', 'Interpreter', 'latex', 'FontSize', 11);
title('Figure 4: Continuous ANFIS-SSM Risk Surface', 'FontSize', 12, 'FontWeight', 'bold');
view([-125, 30]);
grid on;


function R_out = evaluate_anfis_ssm(x1, x2, MF1, MF2, Rules, mf_func)

  mu1 = [mf_func(x1, MF1.H); mf_func(x1, MF1.L); mf_func(x1, MF1.P)];



  mu2 = [mf_func(x2, MF2.L); mf_func(x2, MF2.M); mf_func(x2, MF2.H)];



  num_rules = size(Rules, 1);
  w = zeros(num_rules, 1);
  z = zeros(num_rules, 1);

  for k = 1:num_rules
    idx1 = Rules(k, 1);
    idx2 = Rules(k, 2);
    w(k) = mu1(idx1) * mu2(idx2);
```

```
        z(k) = Rules(k, 3) * x1 + Rules(k, 4) * x2 + Rules(k, 5);
    end

    eps_val = 1e-8;
    w_sum = sum(w) + eps_val;
    w_bar = w / w_sum;

    R_raw = sum(w_bar .* z);
    R_out = min(max(R_raw, 0.0), 1.0);
end
```

ANFIS-SSM Risk Module

Lane-change risk decision module

Original source: ANFIS-SSM Risk Module(3).docx

```
function [Decision, TOR_Warning] = LaneChange_Decision_Logic(TTC_Lead, TTC_Rear, DistToExit,
CurrentLane)



  TTC_Safe_Threshold = 3.5;
  Exit_Critical_Dist = 200;

  Decision = 0;
  TOR_Warning = 0;



  if DistToExit < Exit_Critical_Dist && CurrentLane ~= 1
    TOR_Warning = 2;
  end



  if (TTC_Lead > TTC_Safe_Threshold) && (TTC_Rear > TTC_Safe_Threshold)
    Decision = 1;
  elseif (TTC_Lead <= TTC_Safe_Threshold && TTC_Lead > 1.8)
    Decision = 2;
  else
    Decision = 0;
    TOR_Warning = 1;
  end
end
```

Generate_ALC_Video

Simulation-video generation

Original source: Generate_ALC_Video(3).docx

```
%% =====================================================================
%  Generate High-Quality MP4 Video for Autonomous Lane Change Simulation
%  Target Audience: Journal Reviewers / Technical Reports
%  Author: Mostafa Jalalnezhad
%% =====================================================================
function Generate_ALC_Video()
    clc; close all;



    dt = 0.05;
    T_total = 8;
    t = 0:dt:T_total;
    N = length(t);


    L = 4.8; W = 2.0;


    Vx = 25;
    x_SV = Vx * t;


    lane_width = 3.75;
    y_start = 0;
```

```
y_target = lane_width;

t_start = 2.0;

t_end = 5.5;


y_SV = zeros(1, N);

yaw_SV = zeros(1, N);


for i = 1:N

   if t(i) < t_start

      y_SV(i) = y_start;

      yaw_SV(i) = 0;

   elseif t(i) <= t_end

      tau = (t(i) - t_start) / (t_end - t_start);


      poly = 10*tau^3 - 15*tau^4 + 6*tau^5;

      dpoly = 30*tau^2 - 60*tau^3 + 30*tau^4;


      y_SV(i) = y_start + (y_target - y_start) * poly;

      vy = (y_target - y_start) * dpoly / (t_end - t_start);

      yaw_SV(i) = atan2(vy, Vx);

   else

      y_SV(i) = y_target;

      yaw_SV(i) = 0;

   end

end
```

```
x_FTV = 35 + 20 * t;
y_FTV = zeros(1, N);


x_RTV = -25 + 24 * t;
y_RTV = lane_width * ones(1, N);


video_filename = 'ALC_Simulation_Video.mp4';
writerObj = VideoWriter(video_filename, 'MPEG-4');
writerObj.FrameRate = 20; % 20 fps
writerObj.Quality = 100;
open(writerObj);


hFig = figure('Color', [0.15 0.15 0.15], 'Position', [100 100 1100 500]);

for i = 1:N
   clf(hFig);
   hold on; box on; grid on;
   set(gca, 'Color', [0.2 0.2 0.2], 'XColor', 'w', 'YColor', 'w', 'GridColor', [0.4 0.4 0.4]);
```

```
x_min = x_SV(i) - 30;
x_max = x_SV(i) + 60;

plot([x_min x_max], [-lane_width/2 -lane_width/2], 'w-', 'LineWidth', 2);
plot([x_min x_max], [lane_width*1.5 lane_width*1.5], 'w-', 'LineWidth', 2);
plot([x_min x_max], [lane_width/2 lane_width/2], 'w--', 'LineWidth', 1.5);

plot(x_SV(1:i), y_SV(1:i), 'c--', 'LineWidth', 1.5);

draw_vehicle(x_SV(i), y_SV(i), yaw_SV(i), L, W, [0 0.8 1], 'Subject Vehicle (L3 ALC)');

draw_vehicle(x_FTV(i), y_FTV(i), 0, L, W, [1 0.3 0.3], 'Front Target Vehicle');
draw_vehicle(x_RTV(i), y_RTV(i), 0, L, W, [1 0.6 0.2], 'Rear Target Vehicle');

fov_x = [x_SV(i), x_SV(i) + 25*cos(yaw_SV(i)+deg2rad(15)), x_SV(i) + 25*cos(yaw_SV(i)-
deg2rad(15))];
fov_y = [y_SV(i), y_SV(i) + 25*sin(yaw_SV(i)+deg2rad(15)), y_SV(i) + 25*sin(yaw_SV(i)-deg2rad(15))];
fill(fov_x, fov_y, [0 1 0], 'FaceAlpha', 0.15, 'EdgeColor', 'g', 'LineStyle', ':');

xlim([x_min x_max]);
```

```
ylim([-lane_width, lane_width*2]);

xlabel('Longitudinal Position X [m]', 'Color', 'w', 'FontSize', 11, 'FontWeight', 'bold');

ylabel('Lateral Position Y [m]', 'Color', 'w', 'FontSize', 11, 'FontWeight', 'bold');

title(sprintf('SAE Level 3 Autonomous Lane Change (ALC) Simulation | Time: %.2f s', t(i)), ...

      'Color', 'w', 'FontSize', 13, 'FontWeight', 'bold');



if t(i) < t_start

    status_text = 'Status: Lane Keeping (ACC Active)';

    status_color = [0.2 1 0.2];

elseif t(i) <= t_end

    status_text = 'Status: Executing Autonomous Lane Change (ALC)';

    status_color = [0 0.8 1];

else

    status_text = 'Status: Lane Change Completed (Stable Tracking)';

    status_color = [0.2 1 0.2];

end

text(x_min + 3, lane_width*1.7, status_text, 'Color', status_color, ...

     'FontSize', 11, 'FontWeight', 'bold', 'BackgroundColor', [0 0 0 0.6]);


drawnow;



frame = getframe(hFig);

writeVideo(writerObj, frame);
```

```
    end

    close(writerObj);

end

function draw_vehicle(x, y, yaw, L, W, color, label_text)

    R = [cos(yaw), -sin(yaw); sin(yaw), cos(yaw)];
    x_box = [-L/2, L/2, L/2, -L/2];
    y_box = [-W/2, -W/2, W/2, W/2];

    pts = R * [x_box; y_box];
    fill(x + pts(1,:), y + pts(2,:), color, 'FaceAlpha', 0.85, 'EdgeColor', 'w', 'LineWidth', 1.2);

    front_pt = R * [L/2; 0];
    plot(x + front_pt(1), y + front_pt(2), 'wo', 'MarkerFaceColor', 'w', 'MarkerSize', 4);

    text(x - L/2, y + W + 0.5, label_text, 'Color', color, 'FontSize', 8, 'FontWeight', 'bold');
end
```

plot

Additional plotting code

Original source: plot(3).docx

```
[K_sta,V] = eig(K_sta);
[X,Y,Z] = ellipsoid(0,0,0,1/sqrt(K_sta(1,1)),1/sqrt(K_sta(2,2)),1/sqrt(K_sta(3,3)));

figure1;
surf(X,Y,Z); axis equal;
title('Static Stiffness Ellipsoid');
xlabel('X'); ylabel('Y'); zlabel('Z');

%.......................................................

figure2;
stem(omega/(2*pi),'filled');
xlabel('Mode number');
ylabel('Frequency (Hz)');
title('Natural Frequencies');
grid on;

%..............................................................

figure3;
plot(best.cost,'LineWidth',2);
xlabel('Iteration');
```

```
ylabel('Fitness');

title('PSO Convergence');

grid on;


%.............................................................


figure;

plot(bestPath,'-o','LineWidth',2);

xlabel('Step');

ylabel('State index');

title('Fig.5 Optimization state path');

grid on;

%.................................................
```
